# Sec-mediated secretion by *Coxiella burnetii*

**DOI:** 10.1186/1471-2180-13-222

**Published:** 2013-10-05

**Authors:** Christopher M Stead, Anders Omsland, Paul A Beare, Kelsi M Sandoz, Robert A Heinzen

**Affiliations:** 1 Coxiella Pathogenesis Section, Laboratory of Intracellular Parasites, Rocky Mountain Laboratories, National Institute of Allergy and Infectious Diseases, National Institutes of Health, Hamilton, MT 59840, USA; 2 Host-Parasite Interactions Section, Laboratory of Intracellular Parasites, Rocky Mountain Laboratories, National Institute of Allergy and Infectious Diseases, National Institutes of Health, Hamilton, MT 59840, USA

**Keywords:** *Coxiella*, Secretion, Signal sequence, Outer membrane vesicles, Pili, Q fever, Sec-mediated

## Abstract

**Background:**

*Coxiella burnetii* is a Gram-negative intracellular bacterial pathogen that replicates within a phagolysosome-like parasitophorous vacuole (PV) of macrophages. PV formation requires delivery of effector proteins directly into the host cell cytoplasm by a type IVB secretion system. However, additional secretion systems are likely responsible for modification of the PV lumen microenvironment that promote pathogen replication.

**Results:**

To assess the potential of *C. burnetii* to secrete proteins into the PV, we analyzed the protein content of modified acidified citrate cysteine medium for the presence of *C. burnetii* proteins following axenic (host cell-free) growth. Mass spectrometry generated a list of 105 *C. burnetii* proteins that could be secreted. Based on bioinformatic analysis, 55 proteins were selected for further study by expressing them in *C. burnetii* with a C-terminal 3xFLAG-tag. Secretion of 27 proteins by *C. burnetii* transformants was confirmed by immunoblotting culture supernatants. Tagged proteins expressed by *C. burnetii* transformants were also found in the soluble fraction of infected Vero cells, indicating secretion occurs *ex vivo*. All secreted proteins contained a signal sequence, and deletion of this sequence from selected proteins abolished secretion. These data indicate protein secretion initially requires translocation across the inner-membrane into the periplasm via the activity of the Sec translocase.

**Conclusions:**

*C. burnetii* secretes multiple proteins, *in vitro* and *ex vivo*, in a Sec-dependent manner. Possible roles for secreted proteins and secretion mechanisms are discussed.

## Background

*Coxiella burnetii* is a highly infectious Gram-negative intracellular bacterium that causes the zoonosis Q fever [1]. Central to *C. burnetii* pathogenesis is the ability to proliferate within a parasitophorous vacuole (PV) of macrophages that has characteristics of a large phagolysosome [2,3]. By unknown mechanisms, the pathogen can resist the degradative activities of the vacuole while exploiting the biochemical and biophysical properties of the PV to promote robust intracellular replication [4,5].

The *C. burnetii* PV is a unique cellular compartment that can occupy nearly the entire host cell cytoplasm [6]. *C. burnetii* protein synthesis is required for PV interactions with a subset of cellular vesicles that contribute material to the growing vacuole [7,8]. A collection of effector proteins secreted directly into the cytosol by a specialized Dot/Icm type IVB secretion system are considered largely responsible for modulation of host cell functions that promote PV formation [9-15], and *dot/icm* function is clearly necessary for productive infection [9,10,16].

It is reasonable to suspect that modification of the PV microenvironment by additional secretion systems is also important in *C. burnetii* host cell parasitism. Gram-negative bacteria can employ several secretion systems to translocate proteins into the extracellular milieu [17]. However, bioinformatic analysis of the *C. burnetii* genome reveals canonical components of only a type I secretion system with the presence of a *tolC* homolog [18,19]. Type I secretion is typically a one step process that transports proteins directly from the bacterial cytoplasm into the surrounding environment [20]. However, a small number of proteins, such as heat-stable enterotoxins I and II of *Escherichia coli*[21,22], and an ankyrin repeat protein of *Rickettsia typhi*[23], appear to access TolC via the periplasm after transport across the inner membrane by the Sec translocase. *C. burnetii* lacks typical constituents of a type II secretion system [24]. However, the organism encodes several genes involved in type IV pili (T4P) assembly, several of which are homologous to counterparts of type II secretion systems, indicating a common evolutionary origin and possibly a similar function [25]. Accumulating data indicates core T4P proteins can constitute a secretion system [26-30]. In *Francisella novicida*, a collection of T4P proteins form a secretion system that secretes at least 7 proteins [27]. In *Vibrio cholerae*, T4P secrete a soluble colonization factor required for optimal intestinal colonization of infant mice [30]. *Dichelobacter nodosus* secrete proteases in a T4P-dependent manner [29,31].

Like the well-studied type II secretion system of *Legionella pneumophila,* a close phylogenetic relative of *C. burnetii*[18], substrates secreted by T4P are biased towards N-terminal signal sequence-containing enzymes [27,32]. *C. burnetii* encodes several enzymes with predicted signal sequences, such as an acid phosphatase (CBU0335) that inhibits neutrophil NADPH oxidase function and superoxide anion production [33,34]. Along with PV detoxification, *C. burnetii* exoenzymes could presumably degrade macromolecules into simpler substrates that could then be transported by the organism’s numerous transporters [18]. Genome analysis indicates *C. burnetii* possesses a complete Sec translocase for translocation of signal sequence-containing proteins into the periplasm [18,19].

Another secretion mechanism employed by Gram-negative bacteria is release of outer membrane vesicles (OMVs). OMVs capture periplasmic components before the vesicle pinches off from the cell envelope. This 'packaging’ of proteins is thought to provide a protective environment for delivery of the contents. OMVs are implicated in a variety of functions including delivery of virulence factors, killing of competing bacteria, and suppression of host immune responses [35,36].

The discovery of host cell-free growth of *C. burnetii* in acidified citrate cysteine medium (ACCM) [37,38] allowed us to probe culture media for the presence of secreted proteins. Mass spectrometry generated a list of 105 *C. burnetii* proteins in ACCM culture supernatants. Immunoblotting of culture supernatants following growth of *C. burnetii* transformants expressing individual epitope-tagged versions of identified proteins confirmed secretion of 27 of these proteins. Secretion of epitope-tagged proteins also occurred during growth of *C. burnetii* in Vero host cells. An intact N-terminal signal sequence was required for secretion, indicating secreted proteins have a transient periplasmic location.

## Results

### *Coxiella burnetii* proteins are present in growth medium supernatant

**Figure 1:**
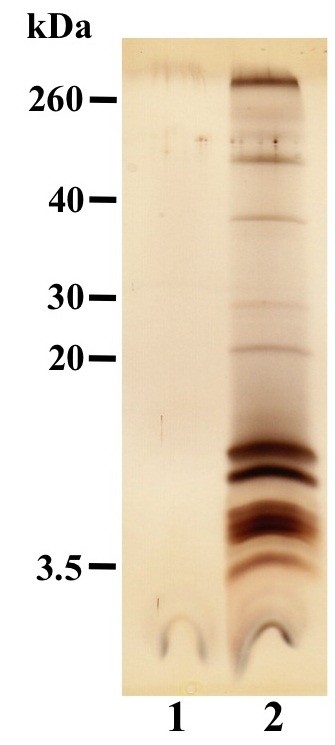
**Multiple *Coxiella burnetii *proteins are present in growth medium supernatant.***C. burnetii* was cultured in modified ACCM-2 for 7 days, then supernatant was collected and analyzed by SDS-PAGE and silver staining. Lane 1, uninoculated media; lane 2, *C. burnetii* growth media.

The Dot/Icm type IVB secretion system of *C. burnetii* has been extensively studied [9,10,39]. However, little is known about other secretion systems of *C. burnetii* that are presumably important for intracellular parasitism. To determine if *C. burnetii* secretes proteins during axenic growth, bacteria were cultivated in ACCM-2 without neopeptone to eliminate media proteins. Following 7 days of growth, supernatant was concentrated and analyzed by SDS-PAGE and silver staining (Figure 1). Many proteins were detected, with the majority having a molecular weight below 20 kDa. In a discovery experiment to generate a list of potentially secreted proteins to further investigate, SDS-PAGE was conducted again and proteins stained with Coomassie G-250 to allow analysis by microcapillary reverse-phase HPLC nano-electrospray tandem mass spectrometry (μLC/MS/MS). A list of 105 proteins was generated (Additional files 1 and 2) with functions assigned based on the annotated genome of the *C. burnetii* Nine Mile RSA493 reference strain [18]. Sixteen proteins were annotated as hypothetical exported proteins, which represents 36% of the total proteins with this annotation in the predicted *C. burnetii* proteome [18]. Twenty-nine proteins, such as translation initiation factor 1 (InfA) and ribosomal protein subunit L31P (RpmE), were predicted as cytoplasmic using the PSORTb v3.0.2 bacterial protein subcellular localization prediction program [40]. This result could be explained by a small amount of bacterial lysis releasing abundant cytoplasmic proteins that are then detected by highly sensitive mass spectrometry. The only Dot/Icm type IVB secretion system substrate detected was CBU0937 [39]. However, type IVB-dependent secretion of CBU0937 was demonstrated using *L. pneumophila* as a surrogate host, and the protein contains a predicted signal sequence, which are typically not associated with Dot/Icm type IVB effectors [41]. Thus, CBU0937 may represent a false positive type IVB effector. Nonetheless, the lack of identified *C. burnetii* Dot/Icm type IVB secretion system substrates in culture supernatants indicates secretion via this mechanism requires host cell-derived signals.

### Expression of epitope-tagged proteins by *C. burnetii* transformants confirms secretion

To confirm active secretion of proteins by *C. burnetii* into growth media, we generated 55 genetic transformants expressing individual proteins, under the control of an inducible TetA promoter, that contain a C-terminal 3xFLAG-tag (Additional file 2). Proteins identified by mass spectometry were selected for epitope-tagging based on predictions obtained using PSORTb, TMHMM [42], SignalP 3.0 [43], BLAST and PubMed bioinformatics tools. Each protein was first analyzed by a BLAST search to identify potential homologs. If a homolog was identified, PubMed searches were conducted to determine if the function and/or the cellular location of the homolog had been characterized. The predicted cellular location was also obtained using PSORTb, TMHMM and SignalP. Based on these analyses, proteins that were unlikely to be secreted, such as malate dehydrogenase, were eliminated from further study.

**Figure 2:**
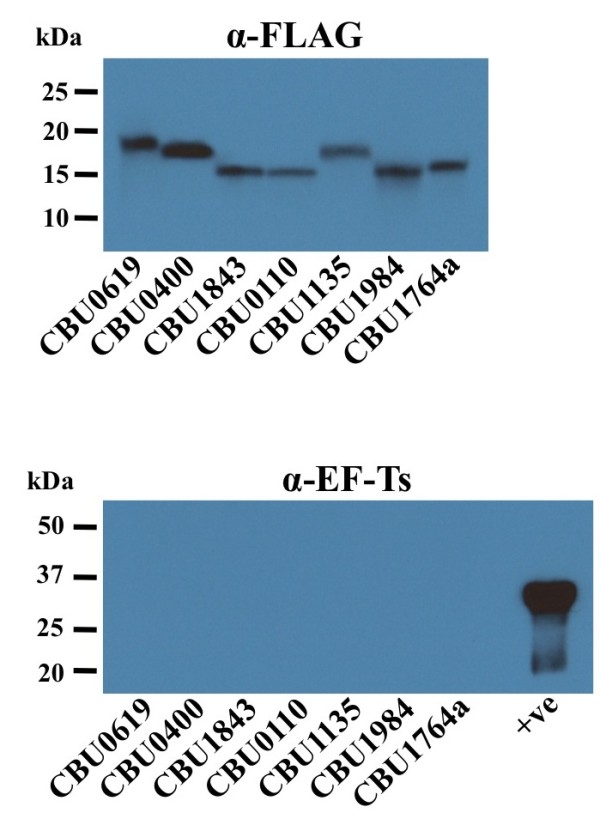
**Expression of FLAG-tagged secretion candidates by *C. burnetii *transformants confirms secretion and not cell lysis.***C. burnetii* transformed with plasmids encoding FLAG-tagged secretion candidates were cultured for 48 h, then expression of tagged protein induced by addition of aTc for 24 h. Supernatants were harvested, TCA precipitated and analyzed by immunoblotting using antibody directed against the FLAG-tag. Immunoblots were also probed with antibody directed against the cytosolic protein EF-Ts to control for bacterial lysis. Whole cell lysate of *C. burnetii* expressing FLAG-tagged CBU1764a was used as a positive control (+ve).

Expression of FLAG-tagged proteins by *C. burnetii* transformants was induced by addition of anhydrotetracycline (aTc) following 48 h of growth of individual transformants in ACCM-2. *C. burnetii* and culture supernatants were harvested 24 h later. Immunoblotting of culture supernatants with anti-FLAG antibody confirmed secretion of 27 of the 55 candidate proteins (Figure 2, Table 1 & Additional file 3). FLAG-tag positive bands were not due to cell lysis as bands were not observed following probing of individual supernatants with antibody directed against EF-Ts, an abundant cytoplasmic protein (Figure 2 & Additional file 3). To ensure negative secretion was not due to a lack of protein expression, bacterial pellets were also analyzed by immunoblotting using the anti-FLAG antibody. With the exception of CBU0089a, CBU1138, CBU1681, and CBU2027, expression of all tagged proteins was confirmed (Additional file 3).

**Table 1:** **Proteins identified in *C. burnetii *** **ACCM-2 culture supernatants by FLAG-tag assay**

**Protein**	**Annotation**	**kDa**
CBU0110	Hypothetical exported protein	13.0
CBU0378	Hypothetical membrane associated protein	15.0
CBU0400	Hypothetical protein	17.0
CBU0482	Arginine-binding protein (ArtI)	29.7
CBU0562a	Hypothetical protein	15.3
CBU0619	Hypothetical exported protein	17.4
CBU0630	FKBP-type peptidyl-prolyl cis-trans isomerase (FkpA)	25.5
CBU0731	Hypothetical exported protein	15.4
CBU0915	Enhanced entry protein EnhB (EnhB1)	19.4
CBU0942	Hypothetical exported protein	14.0
CBU1095	Hypothetical exported protein	17.9
CBU1135	Hypothetical exported protein	15.9
CBU1137	Enhanced entry protein EnhB (EnhB2)	20.9
CBU1173	Hypothetical protein	13.7
CBU1394	Enhanced entry protein EnhA (EnhA5)	19.4
CBU1404	Hypothetical exported protein	12.3
CBU1429a	Hypothetical protein	12.6
CBU1651	Hypothetical membrane associated protein	15.9
CBU1764a	Hypothetical protein	13.5
CBU1822	Superoxide dismutase [Cu-Zn] (SodC)	17.9
CBU1843	Hypothetical exported protein	14.7
CBU1869	Hypothetical exported protein	24.8
CBU1902	Peptidase, M16 family	52.0
CBU1910	Outer membrane protein (Com1)	27.6
CBU1930a	Hypothetical protein	10.4
CBU1984	Hypothetical exported protein	13.8
CBU2072	Hypothetical exported protein	18.4

All 27 secreted proteins contained a predicted signal peptide, with 19 annotated as hypothetical proteins (Table 1). This is not surprising given the unique host-pathogen relationship of *C. burnetii* and the fact that 40.3% of the open reading frames of the Nine Mile reference strain encode hypothetical proteins [18]. Secretion of proteins annotated as enhanced entry proteins (EnhB1, EnhB2 and EnhA5) was confirmed by the FLAG-tag assay. These proteins are homologous to *L. pneumophila* proteins originally thought to facilitate pathogen entry into host cells (EnhA, B & C) [44]. However, a more recent study of *L. pneumophila* EnhC demonstrates a role for this protein in peptidoglycan remodeling [45]. Secretion of Com1 and FkpA (Mip) was confirmed, both of which also have homologs in *L. pneumophila.* Little is known about their roles in *C. burnetii* pathogenesis, although Com1 is known to be outer membrane associated [46] and FkpA has peptidyl-prolyl *cis-trans* isomerase (PPIase) activity [47]. The three remaining secreted proteins with predicted functions were ArtI (CBU0482), an arginine-binding protein, SodC (CBU1822), a Cu-Zn superoxide dismutase, and a M16 family peptidase (CBU1902).

### *C. burnetii* secretes FLAG-tagged proteins during growth in host cells

**Figure 3:**
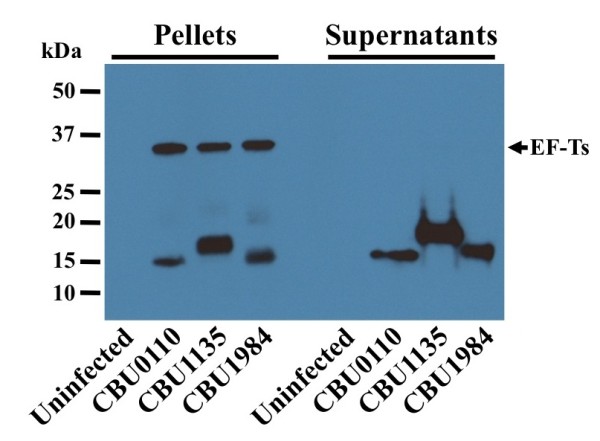
***C. burnetii *secretes proteins during growth in mammalian host cells.** Vero cells were infected for 5 days with *C. burnetii* transformants expressing the FLAG-tagged proteins CBU0110, CBU1135 or CBU1984, then protein expression was induced for 18 h. Host cells were lysed and lysates centrifuged to pellet intact bacteria and cell debris. Proteins present in the pellet and supernatant were separated by SDS-PAGE, transferred to nitrocellulose and analyzed by immunoblotting with antibodies directed against the FLAG-tag and EF-Ts. Uninfected Vero cells were employed as a negative control.

We next examined whether proteins secreted by *C. burnetii* during axenic growth were also secreted during growth in mammalian host cells. Vero cells were infected for 5 days with *C. burnetii* transformants expressing the FLAG-tagged secreted proteins CBU0110, CBU1135 or CBU1984. aTc was added to induce protein expression, then infected cells lysed 18 h later with 0.1% Triton X-100, which solubilizes host cell membranes, but not *C. burnetii*[13]. Cell lysates were centrifuged, then the pellets (containing *C. burnetii* and host cell debris) and supernatants were analyzed by immunoblotting using α-FLAG and α-EF-Ts antibodies (Figure 3). FLAG-tagged proteins were detected in the supernatant, indicating secretion occurs during host cell infection. FLAG-tagged proteins were also present in the bacterial pellet showing the rate of protein synthesis is greater than the rate of secretion. EF-Ts was only detected in the pellet, thereby eliminating bacterial lysis as a source of FLAG-tagged protein in supernatants.

### Secretion of FLAG-tagged proteins requires an intact signal sequence

**Figure 4:**
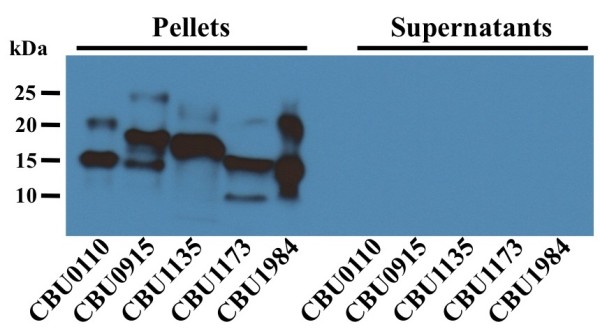
**Secretion requires an intact signal sequence.** Five secreted proteins (CBU0110, CBU0915, CBU1135, CBU1173 and CBU1984) without their respective signal sequence were expressed as described in Figure 2. Pellets and TCA precipitated supernatants were analyzed by immunoblotting using antibody directed against the FLAG-tag.

All verified secreted proteins contained a predicted N-terminal signal sequence. Signal sequences direct transport of proteins across the inner membrane via the Sec translocase [48]. To determine if transport to the periplasm was necessary for secretion, the secreted proteins CBU0110, CBU0915, CBU1135, CBU1173 and CBU1984 were expressed as before, but without their signal sequences. Immunoblotting for C-terminal FLAG-tags revealed that each of the five proteins was present in cell pellets, but not culture supernatants (Figure 4). Thus, a signal sequence, and therefore, a transient periplasmic location is necessary for secretion.

### Potential secretion mechanisms

**Figure 5:**
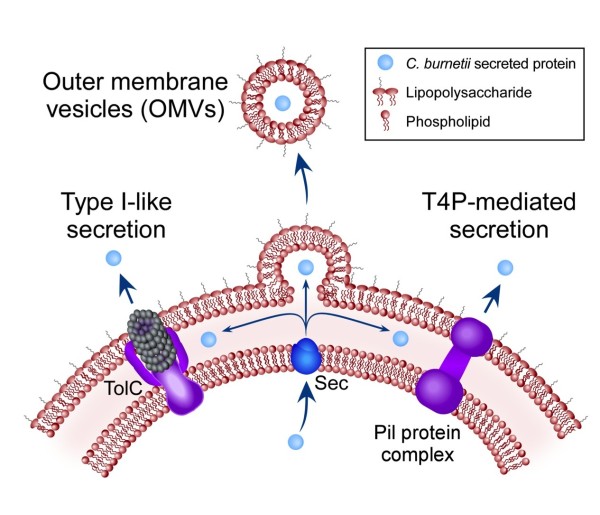
**Possible Sec-mediated secretion mechanisms of *C. burnetii.*** Genome analysis predicts a TolC homolog that could mediate type I-like secretion from the periplasm after proteins are transported across the inner membrane by the Sec translocase. *C. burnetii* also encodes a set of core T4P proteins. T4P are evolutionarily related to type II secretion machinery and have been shown to mediate secretion of several proteins by *F. novicida*. Sequestration of periplasmic or surface proteins by OMVs is a third option for release of proteins into media supernatants.

**Figure 6:**
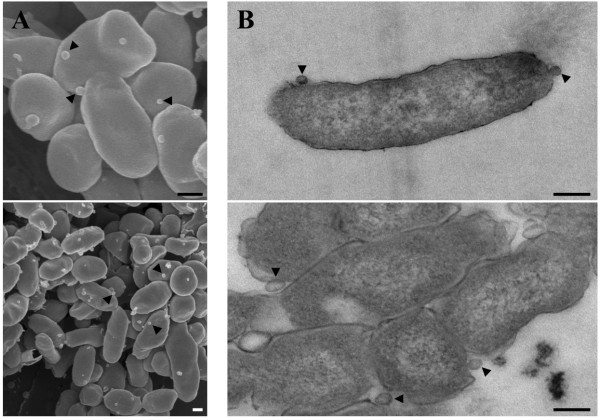
***C. burnetii *produces OMVs. (A)** High and low magnification scanning electron micrographs of *C. burnetii* within the PV of infected Vero cells. Bacteria show membrane blebbing and OMVs (arrowheads). **(B)** Transmission electron micrographs of *C. burnetii* cultured in ACCM-2 for 2 days (upper panel) and 6 days (lower panel) showing membrane blebbing and OMVs (arrowheads). Scale bars = 0.2 μm.

*C. burnetii* Sec-mediated secretion could occur by the mechanisms depicted in Figure 5. Type I-like secretion is predicted by the presence of a *tolC* homolog (CBU0056) in the *C. burnetii* genome. Genome analysis also makes T4P-mediated secretion conceivable as 13 T4P genes are present in the *C. burnetii* Nine Mile reference strain genome (Additional file 4). Eleven of these genes share homologs with the T4P genes of *F. novicida*, a bacterium that employs T4P-mediated secretion (Additional file 4). However, we did not detect pili on the surface of *C. burnetii* using a procedure that visualized pili on *F. tularensis* LVS [49] (Additional file 5). OMVs are produced by a large variety of microbes [50]. Figure 6 depicts what appear to be *C. burnetii* OMVs being produced by bacteria growing in media and within Vero cells, suggesting OMVs contribute to Sec-mediated secretion of proteins by *C. burnetii*.

## Discussion

The importance of protein secretion for bacterial survival and virulence is well documented. Therefore, it was not surprising to discover that *C. burnetii* secretes at least 27 proteins into growth media. This number is similar to the 25 proteins experimentally confirmed by the laboratory of N. P. Cianciotto as secreted by the type II secretion system of *L. pneumophila*, a close relative of *C. burnetii*[32,51]. Heterogeneity among genes encoding secreted proteins is observed between the Nine Mile strain genome used in this study, and the published genomes of the K (Q154), G (Q212), and Dugway (5J108-111) strains. Genes encoding CBU0400 and CBU0562a are missing in K and G, respectively, and four genes are truncated as follows: CBU0110 and CBU1135 (G), CBU1429a (G and K), and CBU1822 (Dugway). All code for hypothetical proteins except CBU1822, which encodes SodC.

Assigning functional roles to these proteins is difficult given the majority are annotated as hypothetical proteins. However, recently developed methods for deleting *C. burnetii* genes could prove useful in defining function [16]. Of the few secreted proteins with predicted functions, SodC, ArtI, and an M16 family peptidase encoded by CBU1902, are of particular interest when considering the phagolysosomal characteristics of the *C. burnetii* PV. SodC is an important virulence factor of intracellular bacteria that degrades superoxide anion generated by the macrophage oxidative burst, thereby lowering oxidative stress [52]. The Dugway isolate may compensate for the lack of SodC by producing a functional catalase, which the Nile Mile strain apparently lacks [53]. ArtI might compensate for *C. burnetii* arginine auxotrophy [18] by high affinity binding of arginine in what might be a nutrient-limited PV environment. CBU1902 shares homology with Zn metalloendopeptidases, including pitrilysin, an *E. coli* peptidase that is capable of cleaving numerous substrates [54]. Thus, CBU1902 could modify the PV environment by cleaving harmful acid hydrolases or degrading complex proteinaceous material into peptides/amino acids suitable for transport by *C. burnetii.* Proteins secreted by Sec-mediated processes are clearly important for host cell modifications that benefit pathogen replication [24]. For example, *Francisella* spp. secrete an acid phosphatase (AcpA), both *in vitro* and *ex vivo*, that has been shown in macrophages to dephosphorylate components of the NADPH oxidase system. This suppression of the oxidative burst promotes intracellular survival and subsequent replication of the pathogen [55,56]. Interestingly, a similar scenario is invoked for the acid phosphatase of *C. burnetii*[34], although this protein was not among the 105 detected in growth media.

Based on genomic and/or ultrastructural data, we propose three secretion mechanisms/protein complexes that may contribute to Sec-mediated secretion by *C. burnetii*. First, the presence of several T4P genes organized in predicted operons suggests secretion might occur via a cell envelope-spanning complex comprised of T4P proteins. However, we found no evidence of pili-like structures on the surface of *C. burnetii*. To our knowledge, all bacteria that employ T4P-mediated secretion also produce identifiable T4P [26,29,30]. Furthermore, virulent *C. burnetii* strains display notable polymorphisms in *pil* gene composition. Specifically, *pilN* of the Nine Mile strain, *pilC* of the K and G strains, and *pilQ* of the G and Dugway strains, are frameshifted and likely non-functional [18]. PilC and PilQ are necessary for secretion by *F. novicida*[27]. All strains also lack *pilP,* which is required for T4P production in several bacteria [57-60]. The incomplete and heterogeneous repertoire of *C. burnetii* T4P genes suggests the gene complement is undergoing genetic decay [18]. Second, secretion could occur by type I-like secretion. However, this process has been documented in relatively few bacteria and is usually responsible for secretion of a small number of proteins [20,23]. Thus, if type I-like secretion is employed by *C. burnetii,* it would likely be responsible for a small fraction of the secreted proteins. Third, and our favored hypothesis, is that the majority of proteins are secreted by OMVs. This idea is supported by EM showing obvious membrane blebbing and OMV production during growth of *C. burnetii* in media and within mammalian host cells.

The possibility that *C. burnetii* proteins are secreted by OMVs is intriguing given the harsh environmental conditions of the PV lumen. The PV displays properties of a phagolysosome, such as acidic pH and active hydrolases, that can quickly degrade *E. coli*[3]. Sequestration of proteins by OMVs could provide a protective environment for delivery of virulence factors to targets within the PV and potentially to cytoplasmic targets should OMV contents transit the PV membrane. OMVs can also act as decoys by sequestering antimicrobial peptides before they reach their intended bacterial targets [61]. In the context of *C. burnetii* infection, it is tempting to speculate that, in addition to sequestering antimicrobial peptides, OMVs might detoxify superoxide by the activity of encapsulated SodC.

## Conclusions

A list of 105 potentially secreted *C. burnetii* proteins was generated by mass spectrometry of culture supernatant. Twenty-seven of these proteins, from a pool of 55 candidate secreted proteins as determined bioinformatically, were confirmed to be secreted using *C. burnetii* transformants expressing FLAG-tagged versions and immunoblotting. Protein secretion was also detected *ex vivo*, suggesting that Sec-mediated secretion contributes to *C. burnetii* pathogenesis. All the secreted proteins had a signal sequence, which was verified as essential for secretion of 5 candidate proteins. Dependence on a signal sequence indicates that TolC, T4P or OMVs could mediate secretion.

## Methods

### *C. burnetii* and mammalian cell lines

*C. burnetii* Nine Mile phase II (RSA439, clone 4) was used in these studies [62]. For general bacterial culture, organisms were propagated microaerobically in ACCM-2 + 1% fetal bovine serum (FBS, Invitrogen) at 37°C [37]. *E. coli* TOP10 (Invitrogen) or Stellar™ (BD Clontech) cells were used for recombinant DNA procedures and cultivated in Luria-Bertani (LB) broth. *E. coli* transformants were selected on LB agar plates containing 10 μg/ml of chloramphenicol. African green monkey kidney (Vero) cells (CCL-81; ATCC) were cultured using RPMI 1640 medium (Invitrogen) containing 10% FBS (Invitrogen).

### SDS-PAGE and silver staining of *C. burnetii* culture supernatants

Two 40 ml *C. burnetii* cultures in ACCM-2 lacking neopeptone were grown in 125 ml Erlenmyer flasks for 7 days with shaking at 75 rpm. The bacteria were combined and pelleted by centrifugation for 5 min at 20,000 × *g*, then the supernatant was passed through a 0.22 μm syringe filter before being concentrated ~400-fold using a 3000 MWCO centrifugal filter (Millipore). The concentrated supernatant was separated by SDS-PAGE using a 16.5% gel and visualized by staining with the Silver Quest kit (Invitrogen).

### Microcapillary reverse-phase HPLC nano-electrospray tandem mass spectrometry (μLC/MS/MS)

Five 40 ml *C. burnetii* cultures in ACCM-2 lacking neopeptone were grown in 125 ml Erlenmyer flasks for 7 days with shaking at 75 rpm. The bacteria were combined and pelleted, then the supernatant passed through a 0.22 μm syringe filter before being concentrated ~500-fold using a 3000 MWCO centrifugal filter. The concentrated supernatant was separated by SDS-PAGE using a 16.5% gel and visualized by staining with Coomassie G-250-based SimplyBlue SafeStain (Invitrogen). The protein containing lane was cut into 10 equal sections that were washed twice with 50% acetonitrile, then stored at -20°C prior to shipping to the Harvard Mass Spectrometry and Proteomics Resource Laboratory, FAS Center for Systems Biology, Northwest Bldg Room B247, 52 Oxford St, Cambridge MA. Gel sections were subjected to tryptic digestion and the resulting peptides sequenced by tandem mass spectrometry. Peptides were analyzed by microcapillary reverse-phase HPLC, directly coupled to the nano-electrospray ionization source of an LTQ-Orbitrap XL mass spectrometer (LCMSMS). Using a custom version of Proteomics Browser Suite (PBS; ThermoFisher Scientific), MS/MS spectra were searched against the *C. burnetii* subset of the NCBInr protein database concatenated to sequences of common laboratory contaminants. Methionine was allowed a variable modification for methionine sulfoxide and cysteine a fixed modification of carboxyamidomethyl cysteine. Peptide-spectrum matches were accepted with PBS filter sets to attain an estimated false discovery rate of <1% using a decoy database strategy. Searches were performed with 2 missed cleavages, semi-tryptic, at 30 ppm mass tolerance, accepting only +/- 2.5 ppm. A minimum of 2 unique peptides were required to identify a protein.

### Construction of pJB-CAT-TetRA-3xFLAG

The TetRA promoter/operator fragment was PCR amplified from pMiniTn7T-CAT::TetRA-icmDJB [9] using Accuprime *Pfx* (Invitrogen) and the primers TetRA-pJB-F and TetRA-3xFLAG-R obtained from Integrated DNA Technologies (Additional file 6). pJB-CAT-P1169-3xFLAG [63] was digested with EcoRI and PstI (New England Biolabs) to remove the P1169 promoter that was replaced with the TetRA fragment using the In-Fusion PCR cloning system (BD Clontech).

### Construction of plasmids encoding C-terminal FLAG-tagged proteins and transformation of *C. burnetii*

Genes were PCR amplified with Accuprime *Pfx* and the primer sets listed in Additional file 6. SignalP 3.0 [43] was used to determine the location of signal sequences for the cloning of genes lacking this sequence. pJB-CAT-TetRA-3xFLAG was digested with PstI (New England Biolabs) followed by insertion of gene-encoding PCR products using the In-Fusion PCR cloning system (BD Clontech). *C. burnetii* was transformed with plasmid constructs as previously described [37].

### Immunoblotting of *C. burnetii* transformant culture supernatants

Transformed *C. burnetii* expressing C-terminal 3xFLAG-tagged proteins were cultivated in ACCM-2 + 1% FBS for 48 h, then expression of tagged proteins induced by addition of anhydrotetracycline (aTc, final concentration = 50 ng/ml). Cell pellets and growth medium were collected 24 h after induction. One milliliter of supernatant from each sample was concentrated by trichloroacetic acid (TCA) precipitation (17% final TCA concentration) prior to analysis by immunoblotting. Detection of proteins present in ACCM and/or the bacterial pellet was conducted by immunoblotting following separation of proteins by SDS-PAGE using a 4-20% gradient gel (Pierce). Nitrocellulose membranes were incubated with monoclonal antibodies directed against FLAG (Sigma) or elongation factor Ts (EF-Ts; a generous gift of James Samuel, Texas A&M University) [64]. Reacting proteins were detected using anti-mouse IgG secondary antibodies conjugated to horseradish peroxidase (Pierce) and chemiluminescence using ECL Pico or Femto reagent (Pierce).

### *Ex vivo* secretion assay

The assay was performed essentially as described by Pan *et al.*[13]. Briefly, Vero cells cultured in 6-well tissue culture plates were infected for 5 days with *C. burnetii* expressing 3xFLAG-tagged proteins under the control of a TetA promoter. Protein expression was then induced with aTc (final concentration = 400 ng/ml) for 18 h. Cells were lysed with 0.1% Triton X-100 plus protease inhibitor cocktail (Sigma) in 1× phosphate buffered saline (1.5 mM KH_2_PO_4_, 2.7 mM Na_2_HPO_4_-7H_2_O, 155 mM NaCl, [pH 7.2]). Lysates were centrifuged for 10 min at 16,000 × *g* and the supernatant passed through a 0.22 μM syringe filter before TCA precipitation. Pellet and supernatant samples were separated by SDS-PAGE, transferred to nitrocellulose and probed with anti-FLAG and anti-EF-Ts antibodies.

### Transmission electron microscopy (EM) of *C. burnetii* grown in ACCM-2

*C. burnetii* was grown in ACCM-2 for 2 or 6 days, then the cells were pelleted and fixed in 2.5% (vol/vol) glutaraldehyde with 0.05 M sucrose in 0.1 M sodium cacodylate buffer for 2 h. Cells were post fixed in 0.5% reduced osmium using a Pelco Biowave microwave (Ted Pella) at 250 W under a 15-in Hg vacuum (all other chemical steps retained these settings) for 2 min on/2 min off/2 min on. Next, tannic acid (1%) was added and samples microwaved, followed by addition of 1% uranyl acetate and microwaving. Samples were dehydrated in a graded ethanol series for 1 min under vacuum and infiltrated with 1:3, 1:1, and 3:1 (Epon/Araldite resin/ethanol), microwaved for 5 min on/5 min off/5 min on, then finally embedded in Epon/Araldite resin. Thin sections (80 nm) were cut using a Leica UC6 (Leica Microsystems) and sections stained with 1% uranyl acetate. Samples were viewed on a Hitachi H-7500 transmission electron microscope (Hitachi) at 80 kV, and digital images were acquired with a Hamamatsu XR-100 digital camera system (AMT).

### Scanning EM of *C. burnetii* infected Vero cells

Vero cells infected with *C. burnetii* for 48 h were fixed, postfixed, and dehydrated as described for transmission EM except that 1% reduced osmium was used for postfixation. Samples were then dried to the critical point in a Bal-Tec cpd 030 drier (Balzer). Cells were dry-fractured by very lightly applying a small piece of adhesive tape to the apical surface that was subsequently gently removed. Cells were coated with 75 Å of iridium in an IBS ion beam sputter (South Bay Technology). Samples were imaged on a Hitachi S-4500 scanning electron microscope (Hitachi).

### Transmission EM of negative stained *C. burnetii* and *F. tularensis* LVS

A fixation and staining protocol optimized for preservation and visualization of pili was employed. *F. tularensis* subsp. *holarctica* Live Vaccine Strain (LVS) from a frozen stock was streaked onto a modified Mueller-Hinton plate that was incubated for 48 h at 37°C, 7% CO_2_. Two milliliters of Chamberlain’s defined medium was inoculated with *F. tularensis* LVS at 0.1 OD/ml and grown ~16 h at 37°C, 200 rpm. The cells were pelleted, washed 2× with 1× PBS, then fixed with 4% paraformaldehyde (PFA). *C. burnetii* was cultured for 4 days in ACCM-2 + 1% FBS, the cells pelleted, washed 2× with 1× PBS, then fixed with 4% PFA. A 5 μl aliquot of fixed bacteria was allowed to settle on a formvar/carbon-coated grid for 5 min. Liquid was removed with filter paper and the samples washed with dH_2_O. Samples were stained with 2% ammonium molybdate for 2 min. Remaining stain was removed with filter paper. Samples were viewed on a Hitachi H-7500 transmission electron microscope (Hitachi) at 80 kV, and digital images were acquired with a Hamamatsu XR-100 digital camera system (AMT).

### Availability of supporting data

All supporting data are included as additional files.

## Competing interests

The authors declare they have no competing interests.

## Authors’ contributions

CMS designed and conducted experiments and drafted the manuscript. AO conceived the study and conducted experiments. PAB constructed the expression vector and assisted with cloning. KMS carried out EM experiments. RAH participated in study design and coordination and helped to draft the manuscript. All authors read and approved the final manuscript.

## Supplementary Material

Additional file 1 **Peptide fragments identified in *C. burnetii *** **ACCM culture supernatants by microcapillary HPLC, nano-ESI, MS/MS analysis.** Click here for file

Additional file 2 **List of *C. burnetii* potentially secreted proteins.** Click here for file

Additional file 3 **Expression of FLAG-tagged secretion candidates by *C. burnetii* transformants to confirm secretion.***C. burnetii* transformed with plasmids encoding FLAG-tagged secretion candidates were cultured for 48 h, then expression of tagged protein induced by addition of aTc for 24 h. Supernatants were harvested, TCA precipitated and analyzed by immunoblotting using antibody directed against the FLAG-tag. Supernatants of samples that were positive for secretion were then probed using antibody directed against EF-Ts to rule out cell lysis as a source of protein present in supernatants. Whole cell lysate of *C. burnetii* expressing FLAG-tagged CBU1764a was used as a positive control (+ve). To confirm that proteins not present in supernatants were expressed by *C. burnetii* transformants, lysates of bacterial pellets were probed with antibody directed against the FLAG-tag. Click here for file

Additional file 4 **Comparison of *F. novicida* and *C. burnetii pil* genes.** The *C. burnetii* genome contains 13 *pil* genes, 11 of which are also present in the *F. novicida* genome, a bacterium that employs T4P-mediated secretion. Click here for file

Additional file 5 ***C. burnetii* is not pilliated.** Transmission electron micrographs of negatively stained bacteria show pili on *F. tularensis* LVS (panel A) but not *C. burnetii* (panel B). Scale bars = 0.5 μm. Click here for file

Additional file 6 Primers used in this study. Click here for file
