# Supplementary material for: Sec-mediated secretion by Coxiella burnetii
Source: BMC Microbiol. 2013 Oct 5;13:222. doi: 10.1186/1471-2180-13-222 (PMC3882888; doi:10.1186/1471-2180-13-222)
Supplement: Additional file 1 — Peptide fragments identified in C. burnetii ACCM culture supernatants by microcapillary HPLC, nano-ESI, MS/MS analysis. [file 1471-2180-13-222-S1.pdf]

Sample: RML01TO10-GT
Sample ID: 32382

User: Omsland, A.

Db: cburnetii_frc (10/21/10)
Dir: RML01TO10-GT_CBRPT

Ion Current (Sum): 2.1e8 A B C D E
Ion Current (Avg): 3.1e6 B C E
# MS/MS Spectra: 2968 A D

A gi|206583768|gb|ACI15236.1| **MS/MS Spectra:** 336 **Sum TIC:** 4.1e7 **Avg TIC:** 1.2e5 **Cov:** 94.7% **Uniq:** 69
hypothetical protein CBU_0089a [Coxiella burnetii RSA 493]

(P1)	Sequence (count)	Reference (+addnl)	Σ TIC	Sf_{max}	Scans
(S)	ALLGSEYIIAK (2)	gi|206583768|gb|aci15236.1| +7	1.1e4	0.95	10864-11188
(S)	AQISPQK (1)	gi|206583768|gb|aci15236.1| +7	1.9e4	0.36	5107
(S)	AQISPQKQNK (2)	gi|206583768|gb|aci15236.1| +7	7.6e3	0.43	4081-4506
(N)	ATEGSFNFR (5)	gi|206583768|gb|aci15236.1| +7	1.3e5	0.96	8024-9194
(N)	CKSTGGALEVK (4)	gi|206583768|gb|aci15236.1| +7	2.5e4	0.80	5628-5849
(T)	EGSFNFR (1)	gi|206583768|gb|aci15236.1| +7	1.3e5	0.13	9230
(K)	EGYTQWR (8)	gi|206583768|gb|aci15236.1| +5	3.4e6	0.74	7128-8317
(K)	EGYTQWRLPN (1)	gi|206583768|gb|aci15236.1| +5	8.2e3	0.88	10499
(K)	EGYTQWRLPNCK (8)	gi|206583768|gb|aci15236.1| +5	1.3e5	0.92	8910-10048
(K)	FTVKGANIR (1)	gi|206583768|gb|aci15236.1| +7	1.6e4	0.63	6997
(K)	FTVKGANIRVYVVTITSK (1)	gi|206583768|gb|aci15236.1| +7	4.0e4	0.81	11608
(K)	GANIRVYVVTITSK (8)	gi|206583768|gb|aci15236.1| +7	1.3e5	0.97	9928-10997
(N)	GEPVSALLGSEYIIAK (8)	gi|206583768|gb|aci15236.1| +7	1.8e5	0.97	11603-12900
(N)	GGQLKTQSAQISPQK (2)	gi|206583768|gb|aci15236.1| +7	7.8e3	0.79	5759-5792
(N)	GGQLKTQSAQISPQKQNK (1)	gi|206583768|gb|aci15236.1| +7	2.3e3	0.19	5187
(G)	GQLKTQSAQISPQKQNK (1)	gi|206583768|gb|aci15236.1| +7	4.3e3	0.51	5351
(L)	GSEYIIAK (3)	gi|206583768|gb|aci15236.1| +7	2.7e4	0.61	7537-13572
(K)	GSYKQITCR (12)	gi|206583768|gb|aci15236.1| +7	1.0e6	0.89	4182-5900
(E)	GYTQWR (1)	gi|206583768|gb|aci15236.1| +5	1.1e4	0.24	7853
(C)	KSTGGALEVK (4)	gi|206583768|gb|aci15236.1| +7	3.5e4	0.64	3815-5651
(L)	KTQSAQISPQKQNK (1)	gi|206583768|gb|aci15236.1| +7	3.3e3	0.53	4153
(L)	LGSEYIIAK (2)	gi|206583768|gb|aci15236.1| +7	1.7e4	0.39	7915-8764
(A)	LLGSEYIIAK (1)	gi|206583768|gb|aci15236.1| +7	4.2e3	0.42	10469
(R)	LPNCKSTGGALEVK (2)	gi|206583768|gb|aci15236.1| +7	2.5e4	0.94	6647-7087
(R)	MQYTDSN (3)	gi|206583768|gb|aci15236.1| +7	1.5e4	0.75	5463-5505
(R)	MQYTDSNGGQLK (14)	gi|206583768|gb|aci15236.1| +7	4.1e6	0.97	5970-7066
(R)	MQYTDSNGGQLKTQ (2)	gi|206583768|gb|aci15236.1| +7	1.4e4	0.96	6296-6642
(R)	MQYTDSNGGQLKTQSAQISPQK (7)	gi|206583768|gb|aci15236.1| +7	4.0e5	0.97	6805-7579
(R)	MQYTDSNGGQLKTQSAQISPQKQNK (6)	gi|206583768|gb|aci15236.1| +7	4.0e5	0.98	6133-7070
(K)	NATEGSF (1)	gi|206583768|gb|aci15236.1| +7	8.2e3	0.20	6891
(K)	NATEGSFN (3)	gi|206583768|gb|aci15236.1| +7	4.2e4	0.61	5859-6099
(K)	NATEGSFNFR (27)	gi|206583768|gb|aci15236.1| +7	1.5e6	0.95	6744-10907
(K)	NATEGSFNFRM*QYTDSNGGQLK (6)	gi|206583768|gb|aci15236.1| +7	7.0e4	0.84	8507-9083
(Q)	NQNGEPVSALLGSEYIIAK (2)	gi|206583768|gb|aci15236.1| +7	1.4e4	0.91	12257-12605
(E)	PVSALLGSEYIIAK (5)	gi|206583768|gb|aci15236.1| +7	3.0e4	0.97	11553-12379
(N)	QNGEPVSALLGSEYIIAK (1)	gi|206583768|gb|aci15236.1| +7	4.7e3	0.89	12603
(K)	QNKFTVK (1)	gi|206583768|gb|aci15236.1| +7	1.7e4	0.25	5833
(A)	QNQNGEPVSALLGSEYIIAK (14)	gi|206583768|gb|aci15236.1| +7	8.8e5	0.97	11473-12947
(T)	QSAQISPQK (1)	gi|206583768|gb|aci15236.1| +7	7.6e3	0.25	4756
(T)	QSAQISPQKQNK (1)	gi|206583768|gb|aci15236.1| +7	3.7e3	0.14	4201
(M)	QYTDSNGGQLK (3)	gi|206583768|gb|aci15236.1| +7	1.5e4	0.82	5006-5523
(V)	SALLGSEYIIAK (1)	gi|206583768|gb|aci15236.1| +7	7.1e3	0.86	11187
(Q)	SAQISPQK (7)	gi|206583768|gb|aci15236.1| +7	1.1e5	0.88	4501-5190
(Q)	SAQISPQKQNK (14)	gi|206583768|gb|aci15236.1| +7	1.4e5	0.77	3279-4604
(G)	SEYIIAK (2)	gi|206583768|gb|aci15236.1| +7	1.7e4	0.77	6767-7508
(I)	SPQKQNK (1)	gi|206583768|gb|aci15236.1| +7	6.5e3	0.19	4715
(K)	STGGALEVK (13)	gi|206583768|gb|aci15236.1| +7	4.0e6	0.90	4799-6529
(K)	STGGALEVKVFA (1)	gi|206583768|gb|aci15236.1| +7	4.9e3	0.90	9151
(K)	STGGALEVKVFAK (12)	gi|206583768|gb|aci15236.1| +7	1.6e5	0.96	8707-9813
(G)	SYKQITCR (1)	gi|206583768|gb|aci15236.1| +7	4.2e3	0.84	5583
(A)	TEGSFNFR (11)	gi|206583768|gb|aci15236.1| +7	4.0e5	0.88	8057-9526
(S)	TGGALEVK (1)	gi|206583768|gb|aci15236.1| +7	10.0e3	0.17	6171
(K)	TQSAQISPQ (3)	gi|206583768|gb|aci15236.1| +7	1.9e4	0.60	4981-5505
(K)	TQSAQISPQK (17)	gi|206583768|gb|aci15236.1| +7	4.3e6	0.94	2104-5147
(K)	TQSAQISPQKQ (3)	gi|206583768|gb|aci15236.1| +7	1.5e4	0.90	4570-5055
(K)	TQSAQISPQKQN (7)	gi|206583768|gb|aci15236.1| +7	5.6e4	0.96	4474-5074
(K)	TQSAQISPQKQNK (18)	gi|206583768|gb|aci15236.1| +7	8.3e6	0.97	3250-4783
(K)	TQSAQISPQKQNKFTVK (10)	gi|206583768|gb|aci15236.1| +7	2.2e5	0.84	5056-6769
(K)	TQSAQISPQKQNKFTVKG (1)	gi|206583768|gb|aci15236.1| +7	2.4e4	0.15	6981
(K)	VFAKGSYK (1)	gi|206583768|gb|aci15236.1| +7	1.1e4	0.31	5725
(K)	VFAKGSYKQITCR (2)	gi|206583768|gb|aci15236.1| +7	2.2e4	0.93	6599-6606
(P)	VSALLGSEYIIAK (1)	gi|206583768|gb|aci15236.1| +7	3.3e3	0.30	11609
(Y)	VVTITSK (1)	gi|206583768|gb|aci15236.1| +7	7.5e3	0.28	8779
(R)	VYVVTITSK (19)	gi|206583768|gb|aci15236.1| +7	1.0e7	0.95	7891-9036
(R)	VYVVTITSKEGYTQWR (4)	gi|206583768|gb|aci15236.1| +5	5.2e4	0.97	9888-10869
(S)	YKQITCR (1)	gi|206583768|gb|aci15236.1| +7	3.4e3	0.53	5442
(Q)	YTDSNGGQLK (3)	gi|206583768|gb|aci15236.1| +7	2.0e4	0.67	4662-6593
(G)	YTQWR (1)	gi|206583768|gb|aci15236.1| +5	6.9e3	0.32	8270
(V)	YVVTITSK (3)	gi|206583768|gb|aci15236.1| +7	9.1e4	0.87	8391-9094

B gi|29541708|gb|AAO90647.1| **MS/MS Spectra:** 74 **Sum TIC:** 3.9e7 **Avg TIC:** 5.2e5 **Cov:** 68.2% **Uniq:** 25
hypothetical membrane associated protein [Coxiella burnetii RSA 493]

(P1)	**Sequence (count)**	**Reference (+addnl)**	**Σ TIC**	**Sf_{max}**	**Scans**
(D)	ATGGVAFLTITVDK (1)	gi|29541708|gb|aao90647.1| +13	3.8e3	0.62	11508
(N)	CAGTNVSVRPGSADK (3)	gi|29541708|gb|aao90647.1| +11	1.0e5	0.90	5684-5716
(A)	GKYTLVTPHTPHLQQGWSSANPQDVNR (5)	gi|29541708|gb|aao90647.1| +5	1.1e7	0.99	9185-9549
(D)	GQKPNHGATGM*VEIN (1)	gi|29541708|gb|aao90647.1| +13	9.2e3	0.54	6077
(K)	GQYVTWAD (2)	gi|29541708|gb|aao90647.1| +13	3.1e4	0.58	9106-10414
(K)	GQYVTWADDGQKPN (1)	gi|29541708|gb|aao90647.1| +13	1.8e4	0.96	8812
(K)	GQYVTWADDGQKPNHGATGM*VEIN (6)	gi|29541708|gb|aao90647.1| +13	1.7e7	0.83	8262-10448
(K)	GQYVTWADDGQKPNHGATGMVEIN (4)	gi|29541708|gb|aao90647.1| +13	2.3e6	0.91	10160-10460
(A)	GTNVSVRPGSADK (1)	gi|29541708|gb|aao90647.1| +11	1.5e4	0.58	5350
(G)	GVAFLTITVDK (1)	gi|29541708|gb|aao90647.1| +13	4.7e3	0.72	12611
(N)	HGATGMVEIN (1)	gi|29541708|gb|aao90647.1| +13	8.5e3	0.31	8232
(D)	KQSPGPSPISNDGIK (1)	gi|29541708|gb|aao90647.1| +13	2.3e3	0.88	5784
(H)	LQQGWSSANPQDVNR (1)	gi|29541708|gb|aao90647.1| +13	4.0e4	0.98	8034
(V)	NCAGTNVSVRPGSADK (1)	gi|29541708|gb|aao90647.1| +11	1.1e4	0.84	6170
(R)	PGSADKCTLEK (1)	gi|29541708|gb|aao90647.1| +13	3.1e4	0.95	4955
(K)	QSPGPSPISNDGIK (5)	gi|29541708|gb|aao90647.1| +13	2.0e6	0.50	6255-7924
(K)	QSPGPSPISNDGIKVN (1)	gi|29541708|gb|aao90647.1| +13	3.4e4	0.78	8749
(K)	QSPGPSPISNDGIKVNCAGTNVSVRPGSADK (2)	gi|29541708|gb|aao90647.1| +11	6.7e4	0.29	8623-8666
(I)	TVDKQSPGPSPISNDGIK (1)	gi|29541708|gb|aao90647.1| +13	5.5e3	0.58	6063
(R)	VDATGGVAFLTITVDK (4)	gi|29541708|gb|aao90647.1| +13	9.2e5	0.98	11903-13845
(R)	VDATGGVAFLTITVDKQSPGPSPISNDGIK (1)	gi|29541708|gb|aao90647.1| +13	7.5e3	0.20	12686
(K)	VNCAGTNVSVRPGSAD (2)	gi|29541708|gb|aao90647.1| +11	4.2e4	0.96	5227-5244
(K)	VNCAGTNVSVRPGSADK (8)	gi|29541708|gb|aao90647.1| +11	3.2e6	0.94	4604-6141
(K)	VNCAGTNVSVRPGSADKCTLEK (12)	gi|29541708|gb|aao90647.1| +11	1.0e6	0.95	5300-6923
(N)	VSVRPGSADK (1)	gi|29541708|gb|aao90647.1| +11	4.3e4	0.86	4950
(K)	YTLVTPHTPHLQQGWSSANPQDVNR (7)	gi|29541708|gb|aao90647.1| +5	1.2e6	0.98	8850-10250

C gi|29542539|gb|AAO91473.1| **MS/MS Spectra:** 136 **Sum TIC:** 2.9e7 **Avg TIC:** 2.1e5 **Cov:** 81.5% **Uniq:** 42
hypothetical exported protein [Coxiella burnetii RSA 493]

(P1)	**Sequence (count)**	**Reference (+addnl)**	**Σ TIC**	**Sf_{max}**	**Scans**
(N)	AINPQYQDCGK (2)	gi|29542539|gb|aao91473.1| +13	7.8e4	0.89	4724-6242
(D)	ALLTGCQHAPIHPK (1)	gi|29542539|gb|aao91473.1| +13	1.4e4	0.85	7578
(R)	AQGKTYEQICR (2)	gi|29542539|gb|aao91473.1| +13	1.2e5	0.96	4489-4495
(G)	CQHAPIHPK (1)	gi|29542539|gb|aao91473.1| +13	1.1e4	0.79	3027
(K)	CTPDAIK (3)	gi|29542539|gb|aao91473.1| +13	8.5e4	0.46	5444-5906
(K)	CTPDAIKK (4)	gi|29542539|gb|aao91473.1| +13	2.3e6	0.71	3304-4755
(K)	CTPDAIKKEIDA (1)	gi|29542539|gb|aao91473.1| +13	7.8e3	0.71	7045
(K)	CTPDAIKKEIDAM*RAQGK (2)	gi|29542539|gb|aao91473.1| +13	8.0e4	0.70	8008-8028
(T)	DALLTGCQHAPIHPK (1)	gi|29542539|gb|aao91473.1| +13	4.8e3	0.31	6598
(K)	EIDAM*R (5)	gi|29542539|gb|aao91473.1| +13	2.9e5	0.44	3327-6279
(K)	EIDAM*RAQGK (8)	gi|29542539|gb|aao91473.1| +13	2.2e6	0.90	3219-4623
(K)	EIDAMR (2)	gi|29542539|gb|aao91473.1| +13	2.1e6	0.11	5925-6292
(A)	FNNPFSQLVR (16)	gi|29542539|gb|aao91473.1| +13	5.1e5	0.96	9921-13340
(W)	HDNYSTCNKP (3)	gi|29542539|gb|aao91473.1| +13	6.5e4	0.94	3479-4933
(A)	INPQYQDCGK (1)	gi|29542539|gb|aao91473.1| +13	7.9e3	0.10	4393
(K)	KEIDAM*R (1)	gi|29542539|gb|aao91473.1| +13	5.1e4	0.75	4154
(K)	KEIDAM*RAQGK (2)	gi|29542539|gb|aao91473.1| +13	1.2e5	0.70	2960-2963
(G)	KTYEQICR (1)	gi|29542539|gb|aao91473.1| +13	1.0e4	0.74	5874
(R)	LEHSPVD (5)	gi|29542539|gb|aao91473.1| +13	3.8e5	0.82	4351-5896
(R)	LEHSPVDPTV (4)	gi|29542539|gb|aao91473.1| +13	1.4e5	0.89	6794-8158
(R)	LEHSPVDPTVC (1)	gi|29542539|gb|aao91473.1| +13	1.8e4	0.74	7784
(R)	LEHSPVDPTVCAED (2)	gi|29542539|gb|aao91473.1| +13	5.0e4	0.86	6623-8053
(R)	LEHSPVDPTVCAEDLQYWHD (1)	gi|29542539|gb|aao91473.1| +13	5.3e3	0.60	10468
(R)	LEHSPVDPTVCAEDLQYWHDNYST (1)	gi|29542539|gb|aao91473.1| +13	8.1e4	0.67	11752
(R)	LEHSPVDPTVCAEDLQYWHDNYSTCNKP (4)	gi|29542539|gb|aao91473.1| +13	1.5e7	0.97	10301-11300
(A)	LLTGCQHAPIHPK (2)	gi|29542539|gb|aao91473.1| +13	1.1e4	0.92	5292-5334
(L)	LTGCQHAPIHPK (1)	gi|29542539|gb|aao91473.1| +13	5.1e3	0.38	3806
(R)	NAINPQYQD (1)	gi|29542539|gb|aao91473.1| +13	2.3e5	0.40	7147
(R)	NAINPQYQDCGK (11)	gi|29542539|gb|aao91473.1| +13	4.7e5	0.93	4877-6541
(R)	NAINPQYQDCGKTTD (5)	gi|29542539|gb|aao91473.1| +13	5.4e4	0.96	5105-6485
(F)	NNPFSQLVR (1)	gi|29542539|gb|aao91473.1| +13	1.9e4	0.92	11446
(N)	NPFSQLVR (2)	gi|29542539|gb|aao91473.1| +13	1.1e4	0.78	10009-11201
(N)	PFSQLVR (4)	gi|29542539|gb|aao91473.1| +13	2.1e5	0.85	8079-13102
(D)	PGFCHCFTDALLTGCQHAPIHPK (5)	gi|29542539|gb|aao91473.1| +13	5.0e5	0.97	10354-11426
(N)	PQYQDCGK (3)	gi|29542539|gb|aao91473.1| +13	3.8e4	0.90	3378-6604
(D)	PTVCAEDLQYWHDNYSTCNKP (3)	gi|29542539|gb|aao91473.1| +13	1.6e5	0.97	9655-10836
(C)	TPDAIKK (3)	gi|29542539|gb|aao91473.1| +13	3.5e4	0.49	3302-4772
(K)	TTDPGFCHCFTDA (1)	gi|29542539|gb|aao91473.1| +13	6.1e3	0.79	8635
(K)	TTDPGFCHCFTDALLTGCQHAPIHPK (9)	gi|29542539|gb|aao91473.1| +13	3.8e6	0.95	10420-11777
(K)	TYEQICR (7)	gi|29542539|gb|aao91473.1| +13	3.0e5	0.92	4748-7240
(K)	TYEQICRLEHSPVD (1)	gi|29542539|gb|aao91473.1| +13	4.0e4	0.56	8218
(T)	VCAEDLQYWHDNYSTCNKP (1)	gi|29542539|gb|aao91473.1| +13	1.7e4	0.57	10359
(T)	YEQICR (2)	gi|29542539|gb|aao91473.1| +13	2.1e4	0.85	4768-6463

D gi|206583778|gb|AAO89674.2| **MS/MS Spectra:** 182 **Sum TIC:** 1.4e7 **Avg TIC:** 7.8e4 **Cov:** 55.9% **Uniq:** 27
hypothetical exported protein [Coxiella burnetii RSA 493]

(P1)	Sequence (count)	Reference (+addnl)	Σ TIC	Sf	Scans

				Sf_{max}	
(R)	CDTINPFPPHTFVPCK (27)	gi|206583778|gb|aao89674.2| +13	4.1e6	0.97	9788-11418
(R)	CDTINPFPPHTFVPCKNVDINEK (7)	gi|206583778|gb|aao89674.2| +11	2.2e5	0.97	9670-10815
(D)	GASFDVIK (1)	gi|206583778|gb|aao89674.2| +13	8.2e3	0.22	8282
(D)	GASFDVIKSENTIR (5)	gi|206583778|gb|aao89674.2| +13	6.1e4	0.93	8334-9219
(H)	GTTLCVVNQR (1)	gi|206583778|gb|aao89674.2| +13	5.1e3	0.70	6223
(R)	GVFAVDK (7)	gi|206583778|gb|aao89674.2| +11	8.4e4	0.69	7022-8030
(R)	GVFAVDKQHGTTLCVVN (1)	gi|206583778|gb|aao89674.2| +11	7.0e3	0.79	8922
(R)	GVFAVDKQHGTTLCVVNQR (22)	gi|206583778|gb|aao89674.2| +11	2.7e6	0.99	8068-9264
(D)	KQHGTTLCVVNQR (1)	gi|206583778|gb|aao89674.2| +11	6.6e3	0.89	5165
(K)	NVDINEK (17)	gi|206583778|gb|aao89674.2| +11	2.0e6	0.67	3830-5780
(K)	NVDINEKTY (3)	gi|206583778|gb|aao89674.2| +11	2.6e4	0.96	6269-7323
(K)	NVDINEKTYCLLTDGASFDVIK (2)	gi|206583778|gb|aao89674.2| +11	1.7e4	0.94	12227-12302
(K)	NVDINEKTYCLLTDGASFDVIKSENTIR (1)	gi|206583778|gb|aao89674.2| +11	3.2e3	0.55	11824
(K)	QHGTTLCVVNQR (9)	gi|206583778|gb|aao89674.2| +13	8.1e5	0.88	5523-7196
(R)	QLSVQCLYQK (14)	gi|206583778|gb|aao89674.2| +13	1.8e6	0.93	8821-9924
(R)	QLSVQCLYQKK (12)	gi|206583778|gb|aao89674.2| +13	8.1e5	0.88	8026-9031
(K)	SENTIR (4)	gi|206583778|gb|aao89674.2| +13	1.1e5	0.28	3066-3920
(K)	SENTIRCSNQR (1)	gi|206583778|gb|aao89674.2| +11	1.6e4	0.27	3169
(A)	SSNEAKYHFTR (4)	gi|206583778|gb|aao89674.2| +11	1.4e5	0.95	4964-6327
(A)	SSNEAKYHFTRCDTINPFPPHTFVPCK (1)	gi|206583778|gb|aao89674.2| +11	3.8e3	0.43	9523
(K)	TYCLLTD (1)	gi|206583778|gb|aao89674.2| +13	1.4e4	0.21	9240
(K)	TYCLLTDGASFDVIK (20)	gi|206583778|gb|aao89674.2| +13	9.6e5	0.99	11629-13817
(K)	TYCLLTDGASFDVIKSENTIR (5)	gi|206583778|gb|aao89674.2| +13	7.8e4	0.97	11567-12255
(N)	VDINEK (2)	gi|206583778|gb|aao89674.2| +11	3.4e4	0.46	3805-4071
(D)	VIKSENTIR (2)	gi|206583778|gb|aao89674.2| +13	1.7e4	0.78	3732-5211
(K)	YHFTR (10)	gi|206583778|gb|aao89674.2| +11	1.4e5	0.37	5392-6448
(K)	YHFTRCDTINPFPPHTFVPCK (2)	gi|206583778|gb|aao89674.2| +11	3.5e4	0.97	9843-9928

E gi|206584156|gb|ACI15326.1| **MS/MS Spectra:** 56 **Sum TIC:** 1.4e7 **Avg TIC:** 2.5e5 **Cov:** 75.8% **Uniq:** 16
hypothetical protein CBU_1847b [Coxiella burnetii RSA 493]

(P1)	**Sequence (count)**	**Reference (+addnl)**	**Σ TIC**	Sf_{max}	**Scans**
(S)	AAISNLCNPK (2)	gi|206584156|gb|aci15326.1| +7	2.4e4	0.90	5858-7277
(R)	ENYLVPNFK (2)	gi|206584156|gb|aci15326.1| +7	1.1e5	0.85	10003-11124
(N)	GIFAACSPTTK (2)	gi|206584156|gb|aci15326.1| +7	4.5e4	0.94	7690-8870
(K)	GWYGFLVGVNR (1)	gi|206584156|gb|aci15326.1| +7	1.2e4	0.96	13020
(R)	LRENYLVPNFK (9)	gi|206584156|gb|aci15326.1| +7	1.5e5	0.91	8769-10674
(K)	NDWPQTPSPHFK (5)	gi|206584156|gb|aci15326.1| +7	2.7e6	0.94	8643-9384
(K)	NDWPQTPSPHFKE (1)	gi|206584156|gb|aci15326.1| +7	1.6e5	0.92	9383
(N)	NGIFAACSPTTK (1)	gi|206584156|gb|aci15326.1| +7	3.4e4	0.85	8985
(S)	NNGIFAACSPTTK (3)	gi|206584156|gb|aci15326.1| +7	6.4e4	0.94	8188-9848
(W)	PQTPSPHFK (1)	gi|206584156|gb|aci15326.1| +7	1.9e4	0.77	9338
(P)	SAAISNLCNPK (2)	gi|206584156|gb|aci15326.1| +7	2.9e4	0.94	6020-7449
(R)	TFKTPSAAISNLCNPK (8)	gi|206584156|gb|aci15326.1| +7	1.3e6	0.95	8355-9650
(K)	TPSAAISNLCNPK (10)	gi|206584156|gb|aci15326.1| +7	9.1e6	0.98	7065-8409
(K)	VCGTNETYEELYLVTCDSDTR (1)	gi|206584156|gb|aci15326.1| +3	4.3e3	0.94	11714
(K)	VKGWYGFLVGVNR (7)	gi|206584156|gb|aci15326.1| +7	6.2e4	0.96	12334-13877
(R)	YAQKVCGTN (1)	gi|206584156|gb|aci15326.1| +7	6.0e3	0.91	3606

F gi|206584133|gb|ACI15321.1| **MS/MS Spectra:** 124 **Sum TIC:** 1.1e7 **Avg TIC:** 8.9e4 **Cov:** 76.3% **Uniq:** 27
hypothetical protein CBU_1764a [Coxiella burnetii RSA 493]

(P1)	**Sequence (count)**	**Reference (+addnl)**	**Σ TIC**	Sf_{max}	**Scans**
(K)	DVLISYSYD (1)	gi|206584133|gb|aci15321.1| +7	6.8e3	0.56	9933
(K)	DVLISYSYDVK (17)	gi|206584133|gb|aci15321.1| +7	9.3e5	0.97	9710-13838
(K)	DVLISYSYDVKNEK (8)	gi|206584133|gb|aci15321.1| +7	6.3e5	0.97	8724-9945
(Q)	FIPDKR (5)	gi|206584133|gb|aci15321.1| +15	4.0e6	0.56	4022-5737
(R)	FIPDKRHPEVK (14)	gi|206584133|gb|aci15321.1| +7	7.5e5	0.91	3968-6350
(K)	ISKPVCYR (8)	gi|206584133|gb|aci15321.1| +7	6.6e5	0.76	4227-5918
(K)	IVLSVDAK (18)	gi|206584133|gb|aci15321.1| +7	2.7e6	0.89	7420-8764
(K)	IVLSVDAKQD (1)	gi|206584133|gb|aci15321.1| +7	1.1e4	0.94	7290
(K)	IVLSVDAKQDFK (9)	gi|206584133|gb|aci15321.1| +1	1.7e5	0.95	8463-14985
(K)	KDVLISYSYDVK (2)	gi|206584133|gb|aci15321.1| +7	3.1e4	0.93	8832-9894
(K)	KDVLISYSYDVKNEK (1)	gi|206584133|gb|aci15321.1| +7	2.1e4	0.94	9077
(D)	KKDVLISYSYDVKNEK (1)	gi|206584133|gb|aci15321.1| +7	3.8e3	0.92	6818
(W)	NTLCSPLEIR (1)	gi|206584133|gb|aci15321.1| +7	5.5e3	0.80	9316
(C)	NWHHETSCNK (1)	gi|206584133|gb|aci15321.1| +7	1.1e4	0.22	4191
(S)	QATVICNWHHETSCNK (8)	gi|206584133|gb|aci15321.1| +7	1.5e5	0.87	6201-7701
(K)	QDFKTHCD (1)	gi|206584133|gb|aci15321.1| +1	1.0e4	0.78	3441
(K)	QDFKTHCDK (1)	gi|206584133|gb|aci15321.1| +1	9.7e3	0.25	3037
(K)	QDFKTHCDKK (5)	gi|206584133|gb|aci15321.1| +1	2.4e5	0.62	2821-4226
(K)	QDFKTHCDKKDVLISYSYDVKNEK (3)	gi|206584133|gb|aci15321.1| +1	1.1e5	0.89	7581-7691
(K)	RHPEVK (5)	gi|206584133|gb|aci15321.1| +7	6.0e4	0.85	2411-3084
(N)	THCDKKDVLISYSYDVK (3)	gi|206584133|gb|aci15321.1| +7	2.5e5	0.98	8080-9144
(N)	THCDKKDVLISYSYDVKNEK (3)	gi|206584133|gb|aci15321.1| +7	1.7e5	0.95	7486-8334
(N)	TLCSPLEIR (1)	gi|206584133|gb|aci15321.1| +7	6.5e3	0.70	9393
(N)	TQTGWNTLCSPLEIR (1)	gi|206584133|gb|aci15321.1| +7	3.8e4	0.97	12434
(R)	TVPSRICF (2)	gi|206584133|gb|aci15321.1| +7	1.0e4	0.31	8211-9250
(N)	WHHETSCNK (3)	gi|206584133|gb|aci15321.1| +7	7.8e4	0.93	2867-3998
(G)	WNTLCSPLEIR (1)	gi|206584133|gb|aci15321.1| +7	10.0e3	0.86	12175

G gi|206583990|gb|AAO90608.2| **MS/MS Spectra:** 126 **Sum TIC:** 1.0e7 **Avg TIC:** 7.9e4 **Cov:** 65.2% **Uniq:** 31
hypothetical exported protein [Coxiella burnetii RSA 493]

(P1)	Sequence (count)	Reference (+addnl)	Σ TIC	Sf_{max}	Scans
(V)	ATLNLSGK (1)	gi|206583990|gb|aao90608.2| +13	6.7e3	0.21	11045
(L)	DVEHITYSCK (1)	gi|206583990|gb|aao90608.2| +13	2.4e4	0.95	9058
(R)	EDYNLVLFGTH (1)	gi|206583990|gb|aao90608.2| +11	5.6e3	0.86	12050
(K)	GISPELEGTLSGFYVK (11)	gi|206583990|gb|aao90608.2| +3	5.6e5	0.98	11729-12861
(K)	GISPELEGTLSGFYVKNNTR (1)	gi|206583990|gb|aao90608.2| +3	6.9e3	0.40	11099
(E)	GTLSGFYVK (1)	gi|206583990|gb|aao90608.2| +13	5.5e3	0.29	10161
(G)	ISPELEGTLSGFYVK (1)	gi|206583990|gb|aao90608.2| +3	5.3e3	0.87	12519
(T)	LDVEHITYSCK (1)	gi|206583990|gb|aao90608.2| +13	1.6e4	0.96	9015
(K)	LEPGTSSDYK (5)	gi|206583990|gb|aao90608.2| +13	1.5e5	0.38	4543-5788
(K)	LEPGTSSDYKQ (1)	gi|206583990|gb|aao90608.2| +13	1.6e4	0.64	5743
(K)	LEPGTSSDYKQCEM*R (14)	gi|206583990|gb|aao90608.2| +13	9.0e5	0.93	4583-6755
(K)	LEPGTSSDYKQCEMR (9)	gi|206583990|gb|aao90608.2| +13	1.8e6	0.93	5949-6743
(N)	LSGKLEPGTSSDYK (1)	gi|206583990|gb|aao90608.2| +13	5.6e3	0.89	6817
(E)	PGTSSDYK (3)	gi|206583990|gb|aao90608.2| +13	2.4e4	0.73	5681-5739
(E)	PGTSSDYKQCEM*R (1)	gi|206583990|gb|aao90608.2| +13	9.9e3	0.70	5794
(D)	QEGGSCAYEGSAIIIVSK (1)	gi|206583990|gb|aao90608.2| +13	6.5e3	0.73	9089
(R)	SKTYGNQK (3)	gi|206583990|gb|aao90608.2| +13	1.6e5	0.66	3107-3443
(K)	TTKVFND (1)	gi|206583990|gb|aao90608.2| +13	5.4e3	0.14	5107
(K)	TTKVFNDAVATLNLSGK (8)	gi|206583990|gb|aao90608.2| +13	2.6e5	0.99	10364-11149
(K)	TTLDVEHITY (1)	gi|206583990|gb|aao90608.2| +13	2.0e4	0.96	10426
(K)	TTLDVEHITYS (2)	gi|206583990|gb|aao90608.2| +13	6.6e4	0.94	9098-9552
(K)	TTLDVEHITYSCK (14)	gi|206583990|gb|aao90608.2| +13	4.7e6	0.97	8073-9367
(K)	TTLDVEHITYSCKITLSKG (1)	gi|206583990|gb|aao90608.2| +13	7.5e3	0.65	10162
(K)	TYGNQK (5)	gi|206583990|gb|aao90608.2| +13	6.0e4	0.39	2052-3660
(K)	TYGNQKTTLDVEHITYSCK (10)	gi|206583990|gb|aao90608.2| +13	2.1e5	0.98	8197-8771
(K)	VFNDAVATLN (1)	gi|206583990|gb|aao90608.2| +13	5.3e3	0.19	9851
(K)	VFNDAVATLNLSGK (11)	gi|206583990|gb|aao90608.2| +13	5.8e5	0.98	9953-11359
(K)	VFNDAVATLNLSGKLEPGTSSDYKQCEM*R (1)	gi|206583990|gb|aao90608.2| +13	1.6e4	0.71	10985
(K)	VNGQELK (12)	gi|206583990|gb|aao90608.2| +13	3.1e5	0.68	4314-4930
(K)	VNGQELKTAVALPK (1)	gi|206583990|gb|aao90608.2| +13	8.5e3	0.93	8710
(A)	YEGSAIIIVSK (1)	gi|206583990|gb|aao90608.2| +13	5.4e3	0.86	9586
(D)	YKQCEM*R (1)	gi|206583990|gb|aao90608.2| +13	3.7e3	0.65	3947

H gi|206583885|gb|ACI15263.1| **MS/MS Spectra:** 75 **Sum TIC:** 8.9e6 **Avg TIC:** 1.2e5 **Cov:** 80.7% **Uniq:** 23
hypothetical protein CBU_0562a [Coxiella burnetii RSA 493]

(P1)	Sequence (count)	Reference (+addnl)	Σ TIC	Sf_{max}	Scans
(K)	AQSIFIR (4)	gi|206583885|gb|aci15263.1| +7	3.9e5	0.81	8546-9629
(K)	ATFCGNPEVNPIQAK (11)	gi|206583885|gb|aci15263.1| +7	7.4e5	0.96	7739-9388
(N)	DGASPLTLKPTAELAK (1)	gi|206583885|gb|aci15263.1| +5	2.1e4	0.88	9817
(K)	FSASGIYCTCR (6)	gi|206583885|gb|aci15263.1| +7	3.4e5	0.97	7812-8951
(K)	FSASGIYCTCRED (1)	gi|206583885|gb|aci15263.1| +7	5.4e3	0.89	7762
(K)	FSASGIYCTCREDR (8)	gi|206583885|gb|aci15263.1| +7	1.2e6	0.71	7291-8482
(K)	FSASGIYCTCREDRK (4)	gi|206583885|gb|aci15263.1| +7	1.4e5	0.77	6850-8082
(D)	GASPLTLKPTAELAK (2)	gi|206583885|gb|aci15263.1| +5	3.5e4	0.96	9210-9534
(R)	GDLYM*YYSNSQK (1)	gi|206583885|gb|aci15263.1| +7	1.5e4	0.95	8082
(R)	GDLYMYYSNSQK (2)	gi|206583885|gb|aci15263.1| +7	1.7e4	0.88	9778-10599
(C)	GNPEVNPIQAK (1)	gi|206583885|gb|aci15263.1| +7	6.2e4	0.93	7255
(K)	GSSNITCGSHYDR (4)	gi|206583885|gb|aci15263.1| +7	6.9e5	0.94	5542-5772
(K)	GSSNITCGSHYDRGDLYM*YYSNSQK (2)	gi|206583885|gb|aci15263.1| +7	1.2e5	0.85	7970-9156
(K)	GSSNITCGSHYDRGDLYMYYSNSQK (1)	gi|206583885|gb|aci15263.1| +7	1.9e5	0.90	9745
(N)	ISNDGASPLTLKPTAELAK (1)	gi|206583885|gb|aci15263.1| +5	4.5e4	0.92	9908
(N)	ITCGSHYDR (1)	gi|206583885|gb|aci15263.1| +7	1.0e4	0.43	4999
(R)	KAQSIFIR (12)	gi|206583885|gb|aci15263.1| +7	2.6e5	0.89	7077-8742
(A)	KATFCGNPEVNPIQAK (1)	gi|206583885|gb|aci15263.1| +7	1.5e4	0.92	7225
(Y)	MYYSNSQK (1)	gi|206583885|gb|aci15263.1| +7	2.9e4	0.60	5908
(N)	PEVNPIQAK (1)	gi|206583885|gb|aci15263.1| +7	3.8e4	0.92	7071
(K)	PGVEGISWNK (1)	gi|206583885|gb|aci15263.1| +7	1.2e4	0.96	9866
(F)	SASGIYCTCR (1)	gi|206583885|gb|aci15263.1| +7	2.6e4	0.86	6772
(I)	TCGSHYDR (1)	gi|206583885|gb|aci15263.1| +7	3.0e4	0.12	5778
(K)	VGIGLDVGEHSPDNWCQKPGVEGISWNK (1)	gi|206583885|gb|aci15263.1| +5	4.3e4	0.97	11653
(A)	YVFNISNDGASPLTLKPTAELAK (6)	gi|206583885|gb|aci15263.1| +5	4.4e6	0.97	11761-12361

I uc|1346343|sp|P04264|K2C1_HUMAN **MS/MS Spectra:** 144 **Sum TIC:** 3.8e6 **Avg TIC:** 2.7e4 **Cov:** 58.5% **Uniq:** 52
CON Keratin, type ii cytoskeletal 1 (cytokeratin 1) (K1) (CK 1) (67 KD cytokeratin) (hair alpha protein) gi|186772 (M98776) keratin 1 [Homo sapiens]

(P1)	Sequence (count)	Reference (+addnl)	Σ TIC	Sf_{max}	Scans
(K)	AEAESLYQSK (3)	uc|1346343|sp|p04264|k2c1_hum +3	3.8e4	0.95	6452-6656
(K)	AEAESLYQSKYEELQITAGR (2)	uc|1346343|sp|p04264|k2c1_hum +3	3.3e4	0.98	10038-10044
(K)	DVDGAYM*TK (9)	uc|1346343|sp|p04264|k2c1_hum +3	1.0e5	0.92	4649-6740
(K)	DVDGAYM*TKVDLQAK (3)	uc|1346343|sp|p04264|k2c1_hum +3	3.6e4	0.70	7375-7860
(K)	DVDGAYMTK (3)	uc|1346343|sp|p04264|k2c1_hum +3	3.3e4	0.93	6723-6784
(K)	DVDGAYMTKVDLQAK (1)	uc|1346343|sp|p04264|k2c1_hum +3	1.5e4	0.69	9157
(R)	DYQELM*NTK (2)	uc|1346343|sp|p04264|k2c1_hum +2	1.6e4	0.92	5861-6490
(Y)	EELQITAGR (1)	uc|1346343|sp|p04264|k2c1_hum +4	1.1e4	0.87	7672
(E)	EREQIK (7)	uc|1346343|sp|p04264|k2c1_hum +22	1.1e5	0.82	2531-3407
(K)	FASFIDKVR (3)	uc|1346343|sp|p04264|k2c1_hum +28	2.1e4	0.95	9115-10370

(R)	FSSCGGGGGSFGAGGGFGSR (1)	uc|1346343|sp|p04264|k2c1_hum +1	1.5e4	0.98	8874
(R)	FVSTTYSGVTR (1)	uc|1346343|sp|p04264|k2c1_hum +2	1.2e4	0.82	7626
(R)	GENALKDAK (4)	uc|1346343|sp|p04264|k2c1_hum +3	3.9e4	0.81	4101-4586
(R)	GGGGGGYGSGGSSYGSGGGSYGSGGGGGGGR (4)	uc|1346343|sp|p04264|k2c1_hum +2	6.1e4	0.97	4253-6216
(S)	GGGSSGGSIGGR (1)	uc|1346343|sp|p04264|k2c1_hum +1	9.1e3	0.81	4198
(R)	GSGGGSSGGSIGGR (6)	uc|1346343|sp|p04264|k2c1_hum +1	2.9e5	0.96	3164-4273
(R)	GSGGGSSGGSIGGRG (1)	uc|1346343|sp|p04264|k2c1_hum +1	6.7e3	0.90	4450
(R)	GSGGGSSGGSIGGRGSSSGGVK (1)	uc|1346343|sp|p04264|k2c1_hum +1	3.4e3	0.57	3514
(Y)	GSGGGSYGSGGGGGGGR (1)	uc|1346343|sp|p04264|k2c1_hum +2	6.4e3	0.79	4114
(G)	GSSGGSIGGR (1)	uc|1346343|sp|p04264|k2c1_hum +1	8.9e3	0.73	4126
(R)	GSYGSGGSSYGSGGGSYGSGGGGGGHGSYGSGSSSGGYR (1)	uc|1346343|sp|p04264|k2c1_hum +1	1.1e4	0.83	6733
(K)	IEISELNR (1)	uc|1346343|sp|p04264|k2c1_hum +5	4.0e3	0.23	8652
(K)	KDVDGAYM*TK (2)	uc|1346343|sp|p04264|k2c1_hum +3	2.3e4	0.59	4837-4846
(N)	KYEDEINKR (5)	uc|1346343|sp|p04264|k2c1_hum +24	2.0e4	0.86	4878-5391
(R)	LDSELKNM*QDM*VEDYR (1)	uc|1346343|sp|p04264|k2c1_hum +3	7.1e3	0.17	7828
(L)	LEGEESR (1)	uc|1346343|sp|p04264|k2c1_hum +15	4.4e3	0.14	4193
(R)	LLRDYQELMNTK (1)	uc|1346343|sp|p04264|k2c1_hum +2	6.8e3	0.81	9860
(K)	LNDLEDALQQAK (1)	uc|1346343|sp|p04264|k2c1_hum +2	7.9e3	0.98	10149
(R)	LRSEIDNVKK (4)	uc|1346343|sp|p04264|k2c1_hum +3	2.1e4	0.72	3667-5322
(K)	NKYEDEINKR (6)	uc|1346343|sp|p04264|k2c1_hum +19	2.0e5	0.90	3993-6167
(K)	NM*QDM*VEDYR (3)	uc|1346343|sp|p04264|k2c1_hum +3	4.4e4	0.97	6329-7934
(K)	NM*QDMVEDYRNKYEDEINKR (1)	uc|1346343|sp|p04264|k2c1_hum +3	8.4e3	0.15	9724
(K)	NMQDM*VEDYR (2)	uc|1346343|sp|p04264|k2c1_hum +3	4.5e4	0.96	7115-7927
(K)	NMQDMVEDYR (2)	uc|1346343|sp|p04264|k2c1_hum +3	2.4e4	0.96	9489-9558
(L)	NVEIDPEIQK (3)	uc|1346343|sp|p04264|k2c1_hum +4	8.9e4	0.92	8259-8276
(L)	NVEIDPEIQKVK (3)	uc|1346343|sp|p04264|k2c1_hum +4	3.2e4	0.86	6782-8411
(K)	QISNLQQSISDAEQR (2)	uc|1346343|sp|p04264|k2c1_hum +3	2.7e4	0.96	8603-9365
(R)	RVDQLKSDQSR (3)	uc|1346343|sp|p04264|k2c1_hum +2	3.8e4	0.88	4227-4627
(K)	SDQSRLDSELK (1)	uc|1346343|sp|p04264|k2c1_hum +3	1.2e4	0.41	6782
(R)	SEIDNVKK (1)	uc|1346343|sp|p04264|k2c1_hum +3	6.8e3	0.15	4555
(R)	SGGGFSSGSAGIINYQRR (1)	uc|1346343|sp|p04264|k2c1_hum +1	1.4e4	0.68	8844
(G)	SGGGSYGSGGGGGGGR (1)	uc|1346343|sp|p04264|k2c1_hum +2	6.5e3	0.34	4092
(K)	SISISVAR (4)	uc|1346343|sp|p04264|k2c1_hum +1	6.9e4	0.80	7668-8219
(K)	SKAEAESLYQSK (4)	uc|1346343|sp|p04264|k2c1_hum +3	1.4e5	0.97	4178-6367
(K)	SKAEAESLYQSKYEELQITAGR (2)	uc|1346343|sp|p04264|k2c1_hum +3	8.7e4	0.98	9696-9721
(R)	SLDLDSIIAEVK (1)	uc|1346343|sp|p04264|k2c1_hum +1	1.8e4	0.98	12776
(K)	SLNNQFASFIDK (2)	uc|1346343|sp|p04264|k2c1_hum +3	1.8e4	0.98	11497-11519
(K)	SLNNQFASFIDKVR (2)	uc|1346343|sp|p04264|k2c1_hum +3	8.7e4	0.94	9921-12295
(R)	SLVNLGGSK (5)	uc|1346343|sp|p04264|k2c1_hum +1	2.2e5	0.91	8408-8560
(K)	SREREQIK (1)	uc|1346343|sp|p04264|k2c1_hum +3	1.6e4	0.52	3620
(R)	TLLEGEESR (4)	uc|1346343|sp|p04264|k2c1_hum +3	9.4e4	0.89	7132-7311
(R)	TNAENEFVTIK (4)	uc|1346343|sp|p04264|k2c1_hum +3	5.7e4	0.95	8396-9203
(R)	TNAENEFVTIKK (6)	uc|1346343|sp|p04264|k2c1_hum +3	1.4e6	0.97	6094-8134
(R)	VDQLKSDQSR (2)	uc|1346343|sp|p04264|k2c1_hum +3	9.6e3	0.79	4085-4487
(K)	WELLQQVDTSTR (1)	uc|1346343|sp|p04264|k2c1_hum +3	3.3e4	0.98	11336
(K)	YEELQITAGR (5)	uc|1346343|sp|p04264|k2c1_hum +4	3.2e4	0.97	6602-8790

J gi|206583866|gb|ACI15260.1| **MS/MS Spectra:** 42 **Sum TIC:** 3.8e6 **Avg TIC:** 9.1e4 **Cov:** 44.9% **Uniq:** 15
hypothetical protein CBU_0516a [Coxiella burnetii RSA 493]

(P1)	**Sequence (count)**	**Reference (+addnl)**	**Σ TIC**	**Sf_{max}**	**Scans**
(A)	ADAQAITK (6)	gi|206583866|gb|aci15260.1| +7	2.3e5	0.92	3380-4796
(D)	EKTGVDGVEPYYR (1)	gi|206583866|gb|aci15260.1| +7	2.7e3	0.77	6986
(K)	FKIHSYNTSNSCDEK (3)	gi|206583866|gb|aci15260.1| +7	1.8e5	0.98	5521-7029
(K)	FKIHSYNTSNSCDEKTGVDGVEPYYR (1)	gi|206583866|gb|aci15260.1| +7	1.5e5	0.96	8969
(K)	GKYHITYPK (4)	gi|206583866|gb|aci15260.1| +7	6.0e5	0.94	6037-7421
(Y)	HITYPK (1)	gi|206583866|gb|aci15260.1| +7	8.8e3	0.84	7966
(K)	IHSYNTSN (1)	gi|206583866|gb|aci15260.1| +7	1.0e4	0.11	3502
(K)	IHSYNTSNSCD (1)	gi|206583866|gb|aci15260.1| +7	6.6e3	0.93	3898
(K)	IHSYNTSNSCDEK (2)	gi|206583866|gb|aci15260.1| +7	1.9e4	0.96	3536-3541
(K)	IHSYNTSNSCDEKTGVDGVEPYYR (2)	gi|206583866|gb|aci15260.1| +7	6.7e4	0.97	6902-7276
(S)	NSCDEK (1)	gi|206583866|gb|aci15260.1| +7	5.5e3	0.36	3555
(K)	TGVDGVEPYYR (8)	gi|206583866|gb|aci15260.1| +7	9.2e5	0.96	7130-8989
(K)	TGVDGVEPYYRVCGSLKTYCK (1)	gi|206583866|gb|aci15260.1| +7	3.5e3	0.14	8987
(R)	VCGSLKTYCK (1)	gi|206583866|gb|aci15260.1| +7	7.7e3	0.78	4519
(K)	YHITYPK (9)	gi|206583866|gb|aci15260.1| +7	1.6e6	0.93	6202-7988

K gi|29541709|gb|AAO90648.1| **MS/MS Spectra:** 40 **Sum TIC:** 3.6e6 **Avg TIC:** 9.1e4 **Cov:** 66.0% **Uniq:** 11
hypothetical exported protein [Coxiella burnetii RSA 493]

(P1)	**Sequence (count)**	**Reference (+addnl)**	**Σ TIC**	**Sf_{max}**	**Scans**
(L)	DSGATPLLVLHSK (1)	gi|29541709|gb|aao90648.1| +11	1.5e4	0.50	11985
(R)	FYEAIWSAGSGR (6)	gi|29541709|gb|aao90648.1| +11	5.7e5	0.98	10382-11525
(K)	LQLDSGATPLLVLHSK (8)	gi|29541709|gb|aao90648.1| +11	1.3e6	0.97	10896-11954
(K)	MPLAFTCPGEPAR (2)	gi|29541709|gb|aao90648.1| +11	2.8e4	0.98	10321-10598
(K)	QLGNPEPLPGPNNYWTNK (5)	gi|29541709|gb|aao90648.1| +9	5.6e5	0.95	9859-11019
(R)	QTVSIED (1)	gi|29541709|gb|aao90648.1| +11	1.4e5	0.70	7354
(R)	QTVSIEDCPFGS (6)	gi|29541709|gb|aao90648.1| +11	2.1e5	0.96	9363-10609
(K)	TNEISYQVNNR (6)	gi|29541709|gb|aao90648.1| +5	4.6e5	0.97	6444-7540
(W)	VVLTTDNPIIR (1)	gi|29541709|gb|aao90648.1| +11	2.2e4	0.94	10373
(R)	YKLQLDSGATPLLVLH (1)	gi|29541709|gb|aao90648.1| +11	8.2e3	0.96	12856
(R)	YKLQLDSGATPLLVLHSK (3)	gi|29541709|gb|aao90648.1| +11	3.4e5	0.99	10994-12148

L	gi|29541744|gb|AAO90683.1|	**MS/MS Spectra:** 44	**Sum TIC:** 3.6e6	**Avg TIC:** 8.1e4	**Cov:** 61.5%	**Uniq:** 20

hypothetical protein CBU_1173 [Coxiella burnetii RSA 493]

(P1)	**Sequence (count)**	**Reference (+addnl)**	**Σ TIC**	**Sf$_{max}$**	**Scans**
(G)	ASAPFYGGVNQPK (1)	gi|29541744|gb|aao90683.1| +5	2.8e4	0.64	8655
(L)	FSDVNQGELK (1)	gi|29541744|gb|aao90683.1| +5	3.4e3	0.83	5663
(L)	FSDVNQGELKK (1)	gi|29541744|gb|aao90683.1| +5	5.1e3	0.87	4992
(P)	GASAPFYGGVNQPK (1)	gi|29541744|gb|aao90683.1| +5	2.8e4	0.15	7497
(D)	GAYWSYFAPGK (1)	gi|29541744|gb|aao90683.1| +11	4.6e3	0.77	11225
(K)	GGWQVYK (6)	gi|29541744|gb|aao90683.1| +5	7.0e5	0.84	7914-9032
(A)	LFSDVNQGELK (1)	gi|29541744|gb|aao90683.1| +5	8.2e3	0.80	7275
(D)	NPGASAPFYGGVNQPK (1)	gi|29541744|gb|aao90683.1| +5	3.7e4	0.88	8864
(N)	PGASAPFYGGVNQPK (2)	gi|29541744|gb|aao90683.1| +5	9.3e4	0.96	7622-8838
(D)	PTVECLAM*GEK (3)	gi|29541744|gb|aao90683.1| +5	8.9e4	0.96	6519-8463
(K)	QCTIQIGSDN (1)	gi|29541744|gb|aao90683.1| +5	2.4e4	0.80	8071
(K)	QCTIQIGSDNPGASAPFYGGVNQPK (7)	gi|29541744|gb|aao90683.1| +5	1.8e6	0.97	9415-11384
(K)	SDPTVECLA (1)	gi|29541744|gb|aao90683.1| +11	4.0e3	0.26	6805
(K)	SDPTVECLAM*GEK (3)	gi|29541744|gb|aao90683.1| +5	1.8e5	0.81	8349-8466
(K)	SDPTVECLAMGEK (3)	gi|29541744|gb|aao90683.1| +5	1.6e5	0.85	8372-9584
(D)	VNQGELK (1)	gi|29541744|gb|aao90683.1| +5	4.3e3	0.25	3532
(D)	VNQGELKK (1)	gi|29541744|gb|aao90683.1| +5	4.9e3	0.61	3303
(K)	VYQSLDALFSD (1)	gi|29541744|gb|aao90683.1| +5	3.1e3	0.57	12456
(K)	VYQSLDALFSDVNQGELK (1)	gi|29541744|gb|aao90683.1| +5	7.5e4	0.98	13780
(K)	VYQSLDALFSDVNQGELKK (5)	gi|29541744|gb|aao90683.1| +5	2.5e5	0.96	11436-13186
(F)	YGGVNQPK (2)	gi|29541744|gb|aao90683.1| +5	2.6e4	0.93	5356-5366

M	gi|29542629|gb|AAO91563.1|	**MS/MS Spectra:** 74	**Sum TIC:** 3.3e6	**Avg TIC:** 4.4e4	**Cov:** 49.3%	**Uniq:** 13

hypothetical protein CBU_2079 [Coxiella burnetii RSA 493]

(P1)	**Sequence (count)**	**Reference (+addnl)**	**Σ TIC**	**Sf$_{max}$**	**Scans**
(R)	EVVCSYK (14)	gi|29542629|gb|aao91563.1| +14	4.0e5	0.82	4561-6052
(K)	ISGGHGCG (4)	gi|29542629|gb|aao91563.1| +14	4.6e4	0.69	2790-3674
(K)	ITAHLDQASGF (1)	gi|29542629|gb|aao91563.1| +14	8.8e3	0.85	8289
(K)	ITAHLDQASGFCLNVAGKR (4)	gi|29542629|gb|aao91563.1| +14	1.7e5	0.98	8995-14986
(K)	KISGGHGCG (12)	gi|29542629|gb|aao91563.1| +14	9.8e4	0.83	1467-3624
(K)	REVVCSYK (2)	gi|29542629|gb|aao91563.1| +14	3.2e4	0.82	5478-5701
(K)	SGSHPNGTITIDSTGR (7)	gi|29542629|gb|aao91563.1| +14	1.1e6	0.97	5584-7139
(K)	SGSHPNGTITIDSTGRYCIYNNTK (1)	gi|29542629|gb|aao91563.1| +14	1.1e4	0.22	7964
(K)	SGSHPNGTITIDSTGRYCIYNNTKK (1)	gi|29542629|gb|aao91563.1| +14	7.0e4	0.72	7623
(S)	SYQLIIK (12)	gi|29542629|gb|aao91563.1| +14	7.6e5	0.88	8998-10208
(E)	VVCSYK (1)	gi|29542629|gb|aao91563.1| +14	3.4e3	0.33	4404
(R)	YCIYNNTK (6)	gi|29542629|gb|aao91563.1| +14	3.5e5	0.91	6128-7571
(R)	YCIYNNTKK (9)	gi|29542629|gb|aao91563.1| +14	2.5e5	0.79	5352-6862

N	gi|29540920|gb|AAO89864.1|	**MS/MS Spectra:** 74	**Sum TIC:** 3.2e6	**Avg TIC:** 4.3e4	**Cov:** 59.7%	**Uniq:** 40

outer membrane protein [Coxiella burnetii RSA 493]

(P1)	**Sequence (count)**	**Reference (+addnl)**	**Σ TIC**	**Sf$_{max}$**	**Scans**
(N)	AGLPTNKIPATFGSR (1)	gi|29540920|gb|aao89864.1| +11	2.1e4	0.89	7742
(R)	ANAGLPTNK (2)	gi|29540920|gb|aao89864.1| +11	1.4e4	0.31	4053-4117
(R)	ANAGLPTNKIPATFG (2)	gi|29540920|gb|aao89864.1| +11	1.8e4	0.81	8467-8513
(R)	ANAGLPTNKIPATFGSR (10)	gi|29540920|gb|aao89864.1| +11	1.9e6	0.96	7147-9306
(D)	ATTVVGLAKQAR (1)	gi|29540920|gb|aao89864.1| +3	4.5e3	0.68	5973
(V)	AYTYNRANAGLPTNK (1)	gi|29540920|gb|aao89864.1| +11	2.4e3	0.11	5806
(V)	AYTYNRANAGLPTNKIPATFGSR (2)	gi|29540920|gb|aao89864.1| +11	2.6e4	0.91	7898-7937
(K)	DATTVVGLAK (6)	gi|29540920|gb|aao89864.1| +11	6.8e5	0.89	6532-7145
(K)	DATTVVGLAKQAR (2)	gi|29540920|gb|aao89864.1| +3	6.4e4	0.96	6743-6764
(K)	DATTVVGLAKQARL (1)	gi|29540920|gb|aao89864.1| +3	4.7e3	0.69	9141
(R)	FVAPVTH (2)	gi|29540920|gb|aao89864.1| +11	1.3e4	0.16	5579-5804
(A)	GLPTNKIPATFGSR (2)	gi|29540920|gb|aao89864.1| +11	3.4e4	0.85	7604-7626
(R)	GNGGFTFGADLG (2)	gi|29540920|gb|aao89864.1| +11	1.5e4	0.75	10106-10110
(K)	IPATFGSR (3)	gi|29540920|gb|aao89864.1| +11	2.1e4	0.80	6052-7271
(R)	KDATTVVGLAK (4)	gi|29540920|gb|aao89864.1| +11	1.7e4	0.72	5854-6714
(R)	KDATTVVGLAKQAR (1)	gi|29540920|gb|aao89864.1| +3	6.2e3	0.95	5945
(G)	KLGVAYTYNR (1)	gi|29540920|gb|aao89864.1| +11	4.8e3	0.12	6709
(G)	LAAGTYTVK (1)	gi|29540920|gb|aao89864.1| +11	3.2e3	0.16	5620
(K)	LGVAYTYNR (1)	gi|29540920|gb|aao89864.1| +11	5.5e3	0.94	6582
(G)	LLGSSSR (1)	gi|29540920|gb|aao89864.1| +11	1.3e4	0.19	3973
(R)	NASSKRFVAPVTH (1)	gi|29540920|gb|aao89864.1| +11	4.4e3	0.56	5187
(T)	NKIPATFGSR (1)	gi|29540920|gb|aao89864.1| +11	1.6e4	0.13	5918
(Y)	NRANAGLPTNK (3)	gi|29540920|gb|aao89864.1| +11	1.9e4	0.65	3704-3983
(Y)	NRANAGLPTNKIPATFGSR (4)	gi|29540920|gb|aao89864.1| +11	7.9e4	0.94	7243-8356
(K)	RFVAPVTH (1)	gi|29540920|gb|aao89864.1| +11	7.6e3	0.73	5448
(A)	RLLGSSSR (2)	gi|29540920|gb|aao89864.1| +3	1.1e4	0.58	3661-4268
(K)	SGMAYAALK (1)	gi|29540920|gb|aao89864.1| +11	5.8e3	0.55	6677
(R)	SRFWNPLF (1)	gi|29540920|gb|aao89864.1| +11	2.2e4	0.35	12902
(R)	SRFWNPLFA (1)	gi|29540920|gb|aao89864.1| +11	5.3e3	0.42	12654
(Y)	TYNRANAGLPTNKIPATFGSR (2)	gi|29540920|gb|aao89864.1| +11	1.3e4	0.89	7508-7520
(N)	TYVFGK (1)	gi|29540920|gb|aao89864.1| +11	4.3e3	0.79	6656
(G)	VAYTYNR (1)	gi|29540920|gb|aao89864.1| +11	6.1e3	0.16	4983
(A)	VEGGWFYLPK (1)	gi|29540920|gb|aao89864.1| +11	4.5e3	0.88	11168

(K)	VKFTTTGVVNG (1)	gi|29540920|gb|aao89864.1| +11	7.6e3	0.47	7066
(K)	VKFTTTGVVNGLA (2)	gi|29540920|gb|aao89864.1| +11	3.2e4	0.95	8624-8641
(S)	VNAQYTFVPGYR (1)	gi|29540920|gb|aao89864.1| +11	6.4e3	0.82	8570
(T)	YNRANAGLPTNK (1)	gi|29540920|gb|aao89864.1| +11	9.2e3	0.22	4434
(T)	YNRANAGLPTNKIPATFGSR (1)	gi|29540920|gb|aao89864.1| +11	2.5e4	0.81	7494
(A)	YTYNR (1)	gi|29540920|gb|aao89864.1| +11	1.6e4	0.24	3934
(A)	YTYNRANAGLPTNKIPATFGSR (1)	gi|29540920|gb|aao89864.1| +11	6.3e3	0.14	7777

O uc|547749|sp|P13645|K1CJ_HUMAN **MS/MS Spectra:** 93 **Sum TIC:** 2.0e6 **Avg TIC:** 2.1e4 **Cov:** 48.2% **Uniq:** 41
CON Keratin, type i cytoskeletal 10 (cytokeratin 10) (K10) (CK 10)

(P1)	**Sequence (count)**	**Reference (+addnl)**	**Σ TIC**	**Sf_{max}**	**Scans**
(R)	AETECQNTEYQQLLDIK (1)	uc|547749|sp|p13645|k1cj_huma +4	4.6e3	0.96	10977
(R)	ALEESNYELEGK (6)	uc|547749|sp|p13645|k1cj_huma +4	8.2e4	0.98	7432-8498
(K)	DAEAWFNEK (2)	uc|547749|sp|p13645|k1cj_huma +4	2.6e4	0.94	10134-10137
(K)	ELTTEIDNNIEQISSYK (1)	uc|547749|sp|p13645|k1cj_huma +5	8.1e3	0.95	11592
(L)	GGGFSSGGFSGGSFSR (1)	uc|547749|sp|p13645|k1cj_huma +3	5.2e3	0.92	9423
(K)	GSLGGGFSSGGFSGGSFSR (3)	uc|547749|sp|p13645|k1cj_huma +3	5.9e4	0.98	8049-10277
(K)	HGNSHQGEPR (2)	uc|547749|sp|p13645|k1cj_huma +3	5.0e3	0.93	1523-3252
(K)	IKEWYEK (1)	uc|547749|sp|p13645|k1cj_huma +3	1.6e4	0.86	8240
(K)	IKEWYEKHGNSHQGEPR (1)	uc|547749|sp|p13645|k1cj_huma +3	1.0e4	0.85	6196
(K)	IRLENEIQTYR (2)	uc|547749|sp|p13645|k1cj_huma +5	2.7e4	0.96	8870-8877
(R)	ISSSKGSLGGGFSSGGFSGGSFSR (1)	uc|547749|sp|p13645|k1cj_huma +3	3.7e3	0.67	9789
(R)	LAADDFR (3)	uc|547749|sp|p13645|k1cj_huma +24	3.3e4	0.62	7415-8019
(R)	LASYLDKVR (4)	uc|547749|sp|p13645|k1cj_huma +16	3.7e4	0.93	7764-8608
(R)	LENEIQTYR (3)	uc|547749|sp|p13645|k1cj_huma +5	2.0e4	0.92	6799-7895
(R)	LKYENEVALR (6)	uc|547749|sp|p13645|k1cj_huma +5	1.6e5	0.94	7222-8071
(K)	NHEEEM*KDLR (3)	uc|547749|sp|p13645|k1cj_huma +4	2.6e4	0.90	3975-4358
(K)	NHEEEMK (1)	uc|547749|sp|p13645|k1cj_huma +7	6.5e3	0.47	3466
(A)	NILLQIDNAR (1)	uc|547749|sp|p13645|k1cj_huma +7	7.1e3	0.87	10766
(R)	NVSTGDVN (1)	uc|547749|sp|p13645|k1cj_huma +5	7.3e3	0.51	4955
(R)	NVSTGDVNVEM* (1)	uc|547749|sp|p13645|k1cj_huma +5	3.0e3	0.28	6922
(R)	NVSTGDVNVEM*NAAPGVDLTQLLNNM*R (1)	uc|547749|sp|p13645|k1cj_huma +4	1.0e4	0.49	12107
(R)	QSVEADINGLRR (2)	uc|547749|sp|p13645|k1cj_huma +7	2.5e4	0.12	7732-8545
(K)	SEITELR (1)	uc|547749|sp|p13645|k1cj_huma +5	3.9e4	0.73	7485
(K)	SEITELRR (2)	uc|547749|sp|p13645|k1cj_huma +5	1.9e4	0.49	6088-6784
(R)	SGGGGGGGGCGGGGGV (1)	uc|547749|sp|p13645|k1cj_huma +2	7.3e3	0.14	5413
(R)	SGGGGGGGGCGGGGGVSSLR (2)	uc|547749|sp|p13645|k1cj_huma +2	1.4e4	0.97	6064-6077
(N)	SHQGEPR (1)	uc|547749|sp|p13645|k1cj_huma +3	7.4e3	0.77	3304
(K)	SKELTTEIDNNIEQISSYK (3)	uc|547749|sp|p13645|k1cj_huma +5	3.8e4	0.95	11249-11261
(R)	SLLEGEGSSGGGGR (4)	uc|547749|sp|p13645|k1cj_huma +3	1.2e5	0.97	6360-7055
(R)	SQYEQLAEQNR (2)	uc|547749|sp|p13645|k1cj_huma +4	1.6e4	0.97	6302-6978
(R)	SQYEQLAEQNRK (5)	uc|547749|sp|p13645|k1cj_huma +4	3.6e5	0.96	6086-6495
(I)	SSSKGSLGGGFSSGGFSGGSFSR (1)	uc|547749|sp|p13645|k1cj_huma +3	1.2e4	0.94	9634
(K)	SSSSGSVGESSSK (1)	uc|547749|sp|p13645|k1cj_huma +7	2.2e4	0.85	2038
(K)	SSSSGSVGESSSKG (1)	uc|547749|sp|p13645|k1cj_huma +7	5.1e3	0.88	3858
(K)	SSSSGSVGESSSKGP (6)	uc|547749|sp|p13645|k1cj_huma +7	2.5e5	0.96	4170-4747
(K)	SSSSGSVGESSSKGPR (1)	uc|547749|sp|p13645|k1cj_huma +7	6.8e3	0.84	4275
(T)	TDNANILLQIDNAR (1)	uc|547749|sp|p13645|k1cj_huma +5	4.3e3	0.91	11208
(R)	VLDELTLTK (3)	uc|547749|sp|p13645|k1cj_huma +4	3.1e4	0.90	9088-9878
(K)	VTM*QNLNDR (4)	uc|547749|sp|p13645|k1cj_huma +10	3.1e5	0.95	4638-6942
(K)	VTM*QNLNDRLASYLDK (1)	uc|547749|sp|p13645|k1cj_huma +9	8.1e3	0.22	10444
(K)	VTMQNLNDR (2)	uc|547749|sp|p13645|k1cj_huma +10	6.0e4	0.95	6987-7285
(K)	YENEVALR (4)	uc|547749|sp|p13645|k1cj_huma +5	7.6e4	0.94	6585-7283

P gi|29541233|gb|AAO90176.1| **MS/MS Spectra:** 51 **Sum TIC:** 2.0e6 **Avg TIC:** 3.8e4 **Cov:** 66.7% **Uniq:** 12
hypothetical protein CBU_0632 [Coxiella burnetii RSA 493]

(P1)	**Sequence (count)**	**Reference (+addnl)**	**Σ TIC**	**Sf_{max}**	**Scans**
(R)	DEEKEHAAM*VLEWIR (1)	gi|29541233|gb|aao90176.1| +13	3.4e3	0.80	11052
(K)	EHAAM*VLEWIR (1)	gi|29541233|gb|aao90176.1| +13	3.9e3	0.25	11029
(D)	ELSDESR (1)	gi|29541233|gb|aao90176.1| +13	5.0e3	0.59	4419
(R)	EYLFTDKPIVD (1)	gi|29541233|gb|aao90176.1| +13	5.6e3	0.90	10826
(R)	EYLFTDKPIVDHHSGE (15)	gi|29541233|gb|aao90176.1| +13	4.2e5	0.93	8863-9776
(R)	HDPVFDKELR (7)	gi|29541233|gb|aao90176.1| +13	4.0e5	0.95	6862-7761
(-)	M*TSENYHEPIDELSDESREMHRAI (1)	gi|29541233|gb|aao90176.1| +13	1.2e4	0.16	8432
(E)	PIDELSDESR (1)	gi|29541233|gb|aao90176.1| +13	4.0e3	0.94	7032
(R)	RHDPVFDK (1)	gi|29541233|gb|aao90176.1| +13	1.1e4	0.89	5703
(R)	RHDPVFDKELR (11)	gi|29541233|gb|aao90176.1| +13	3.2e5	0.97	6475-7622
(M)	TSENYHEPIDELSDESR (7)	gi|29541233|gb|aao90176.1| +13	6.7e5	0.97	7857-9042
(M)	TSENYHEPIDELSDESREM*HR (3)	gi|29541233|gb|aao90176.1| +13	1.0e5	0.92	7726-8340
(M)	TSENYHEPIDELSDESREMHR (1)	gi|29541233|gb|aao90176.1| +13	5.5e3	0.32	8659

Q uc|P35527|K1C9_HUMAN **MS/MS Spectra:** 104 **Sum TIC:** 1.6e6 **Avg TIC:** 1.5e4 **Cov:** 48.3% **Uniq:** 40
CON Keratin, type I cytoskeletal 9 OS=Homo sapiens GN=KRT9 PE=1 SV=3

(P1)	**Sequence (count)**	**Reference (+addnl)**	**Σ TIC**	**Sf_{max}**	**Scans**
(K)	EVTQLR (1)	uc|p35527|k1c9_human +2	9.3e3	0.21	5557
(R)	FSSSGGGGGGGR (6)	uc|p35527|k1c9_human	1.5e5	0.95	3694-4115
(R)	FSSSSGYGGGSSR (2)	uc|p35527|k1c9_human +2	1.9e4	0.96	3752-5101
(S)	GGGGGGGLGSGGSIR (6)	uc|p35527|k1c9_human +2	3.4e4	0.90	4085-6279
(G)	GGGGLGSGGSIR (1)	uc|p35527|k1c9_human +2	6.1e3	0.21	4074

(S)	GGNYGGGSGSGGGSGGGYGGGSGSR (1)	uc|p35527|k1c9_human +2	1.6e4	0.17	11043
(G)	GGSGGGYGGGSGSR (1)	uc|p35527|k1c9_human +4	7.0e3	0.84	4084
(R)	GGSGGSHGGGSGFGGE (3)	uc|p35527|k1c9_human +2	2.3e4	0.78	5409-5420
(R)	GGSGGSHGGGSGFGGESGG (1)	uc|p35527|k1c9_human +2	3.3e3	0.55	5314
(R)	GGSGGSHGGGSGFGGESGGSYGGGEEASGSGGGYGGGSGK (2)	uc|p35527|k1c9_human +2	5.2e3	0.92	4671-7019
(R)	GGSGGSYGGGGSGGGYGGGSGSR (8)	uc|p35527|k1c9_human +2	7.1e4	0.97	4059-6311
(R)	GGSGGSYGR (3)	uc|p35527|k1c9_human +2	8.5e4	0.53	3507-4002
(R)	HGVQELEIELQSQLSK (1)	uc|p35527|k1c9_human +2	8.4e3	0.98	11936
(R)	HGVQELEIELQSQLSKK (3)	uc|p35527|k1c9_human +2	1.7e4	0.96	10650-11467
(K)	IGLGGRGGSGGSYGR (2)	uc|p35527|k1c9_human +2	2.1e4	0.63	6646-6655
(R)	IKFEM*EQNLR (5)	uc|p35527|k1c9_human +3	9.4e4	0.86	7434-7812
(K)	IQDWYDKK (1)	uc|p35527|k1c9_human +3	2.5e3	0.22	6974
(K)	IQDWYDKKGPAAIQK (1)	uc|p35527|k1c9_human +3	6.3e3	0.50	8189
(K)	KGPAAIQK (2)	uc|p35527|k1c9_human +3	1.3e4	0.86	3923-4240
(R)	LASYLDK (3)	uc|p35527|k1c9_human +20	8.6e4	0.74	7415-7789
(R)	MTLDDFR (4)	uc|p35527|k1c9_human +3	3.2e4	0.82	9389-10121
(R)	QVLDNLTM*EK (1)	uc|p35527|k1c9_human +3	2.4e4	0.78	8380
(S)	RSGGGGGGGLGSGGSIR (4)	uc|p35527|k1c9_human +1	3.4e4	0.91	3769-5735
(R)	SGGGGGGGLGSGGSIR (8)	uc|p35527|k1c9_human +2	2.1e5	0.98	4051-6287
(R)	SGGGGGGGLGSGGSIRSS (1)	uc|p35527|k1c9_human +1	3.7e3	0.72	4702
(R)	SGGGGGGGLGSGGSIRSSY (1)	uc|p35527|k1c9_human +1	9.6e3	0.91	6028
(R)	SGGGGGGGLGSGGSIRSSYSR (2)	uc|p35527|k1c9_human +2	3.8e4	0.40	5505-5518
(K)	SLEDTKNR (2)	uc|p35527|k1c9_human +2	2.0e4	0.66	4157-4250
(S)	SLGGGFGGGSR (1)	uc|p35527|k1c9_human +2	4.7e3	0.30	6673
(Y)	SRFSSSGGGGGGGR (2)	uc|p35527|k1c9_human	1.5e4	0.65	4380-4432
(L)	SRSGGGGGGGLGSGGSIR (6)	uc|p35527|k1c9_human +1	8.1e4	0.97	4594-5850
(V)	SSSGQEVQSSAK (1)	uc|p35527|k1c9_human +3	4.7e3	0.33	4011
(F)	SSSSGYGGGSSR (1)	uc|p35527|k1c9_human +2	7.4e3	0.86	4056
(K)	STM*QELNSR (2)	uc|p35527|k1c9_human +2	1.9e4	0.90	5759-6348
(K)	STMQELNSR (4)	uc|p35527|k1c9_human +2	2.7e5	0.94	5735-6378
(K)	TLLDIDNTR (2)	uc|p35527|k1c9_human +3	4.3e4	0.92	9514-9586
(K)	TLLDIDNTRM*TLDDFR (1)	uc|p35527|k1c9_human +3	4.8e3	0.53	11683
(K)	TLNDM*R (1)	uc|p35527|k1c9_human +3	7.4e3	0.44	4178
(K)	TLNDM*RQEYEQLIAK (2)	uc|p35527|k1c9_human +3	3.3e4	0.30	8782-9502
(K)	TLNDMRQEYEQLIAK (4)	uc|p35527|k1c9_human +3	3.8e4	0.94	11485-11606
(K)	VQALEEANNDLENK (1)	uc|p35527|k1c9_human +3	1.2e4	0.98	8040

R gi|206584179|gb|ACI15331.1| **MS/MS Spectra:** 18 **Sum TIC:** 1.5e6 **Avg TIC:** 8.1e4 **Cov:** 72.9% **Uniq:** 8
hypothetical protein CBU_1930a [Coxiella burnetii RSA 493]

(P1)	Sequence (count)	Reference (+addnl)	Σ TIC	Sf_{max}	Scans
(A)	ACSPFGPGTSSCYNGDTVCGAEK (1)	gi|206584179|gb|aci15331.1| +7	2.8e4	0.96	9122
(R)	EYICGGPSPTYK (5)	gi|206584179|gb|aci15331.1| +7	1.1e6	0.94	6578-7986
(K)	FKDCQIGDPADELFCSK (2)	gi|206584179|gb|aci15331.1| +7	1.8e5	0.98	10368-11404
(K)	FKDCQIGDPADELFCSKR (3)	gi|206584179|gb|aci15331.1| +7	7.9e4	0.98	9824-10904
(K)	NNIDCFECM*VPQS (2)	gi|206584179|gb|aci15331.1| +7	6.0e4	0.92	9731-9756
(K)	NNIDCFECMVPQS (1)	gi|206584179|gb|aci15331.1| +7	3.6e3	0.56	10449
(D)	PADELFCSKR (1)	gi|206584179|gb|aci15331.1| +7	7.4e3	0.52	7271
(K)	QNGKNNIDCFECM*VPQS (1)	gi|206584179|gb|aci15331.1| +7	6.1e3	0.71	7859
(K)	REYICGGPSPTYK (2)	gi|206584179|gb|aci15331.1| +7	4.0e4	0.95	6266-7617

S gi|120575184|gb|EAX31808.1| **MS/MS Spectra:** 27 **Sum TIC:** 1.4e6 **Avg TIC:** 5.2e4 **Cov:** 79.2% **Uniq:** 12
hypothetical protein A35_B0145 [Coxiella burnetii 'MSU Goat Q177']

(P1)	Sequence (count)	Reference (+addnl)	Σ TIC	Sf_{max}	Scans
(G)	DDLRCENGGTVTGVTHK (2)	gi|120575184|gb|eax31808.1| +1	2.7e5	0.97	6473-6485
(K)	DKGDYIWNND (1)	gi|120575184|gb|eax31808.1| +1	1.4e4	0.92	10056
(K)	DKGDYIWNNDGTLTCCKPNQTGSLCP (2)	gi|120575184|gb|eax31808.1| +1	6.5e5	0.96	10889-10952
(R)	EGTHWYTSINIATCK (1)	gi|120575184|gb|eax31808.1| +1	1.1e5	0.96	10859
(K)	ETTM*ACCCSR (1)	gi|120575184|gb|eax31808.1| +1	1.7e4	0.93	4140
(K)	ETTMACCCSR (4)	gi|120575184|gb|eax31808.1| +1	7.4e4	0.90	3873-5304
(N)	GGTVTGVTHK (1)	gi|120575184|gb|eax31808.1| +1	1.4e4	0.19	4641
(K)	GQTTNCCPHGPYSR (5)	gi|120575184|gb|eax31808.1| +1	5.2e4	0.74	4116-5564
(N)	PPEFNFCPK (1)	gi|120575184|gb|eax31808.1| +1	1.8e4	0.71	10628
(R)	SCAPCSTK (6)	gi|120575184|gb|eax31808.1| +1	1.5e5	0.89	3578-4033
(R)	SCAPCSTKETTM*ACCCSR (1)	gi|120575184|gb|eax31808.1| +1	1.4e4	0.80	5199
(Q)	TTNCCPHGPYSR (1)	gi|120575184|gb|eax31808.1| +1	8.4e3	0.86	5720
(T)	WETNPPEFNFCPK (1)	gi|120575184|gb|eax31808.1| +1	3.5e3	0.52	10712

T gi|29541220|gb|AAO90163.1| **MS/MS Spectra:** 29 **Sum TIC:** 1.1e6 **Avg TIC:** 3.7e4 **Cov:** 42.0% **Uniq:** 12
hypothetical exported protein [Coxiella burnetii RSA 493]

(P1)	Sequence (count)	Reference (+addnl)	Σ TIC	Sf_{max}	Scans
(A)	DTSLLQGAIDYSPNK (3)	gi|29541220|gb|aao90163.1| +1	3.2e5	0.97	11069-11408
(A)	DTSLLQGAIDYSPNKISK (2)	gi|29541220|gb|aao90163.1| +1	2.1e5	0.97	10959-11070
(K)	FDWLQGYDGSQPCGSCTGSPSVR (3)	gi|29541220|gb|aao90163.1| +13	6.9e4	0.98	11633-12090
(D)	GSQPCGSCTGSPSVR (3)	gi|29541220|gb|aao90163.1| +13	3.9e4	0.91	5235-5526
(K)	ISKFDWLQGYDGSQPCGSCTGSPSVR (2)	gi|29541220|gb|aao90163.1| +13	2.1e4	0.95	11229-11328
(K)	KVTYFK (2)	gi|29541220|gb|aao90163.1| +9	3.4e4	0.76	6978-7211
(K)	LPLVGQYQYDEGNK (2)	gi|29541220|gb|aao90163.1| +7	2.3e4	0.97	10086-10328
(K)	LPLVGQYQYDEGNKGTLK (4)	gi|29541220|gb|aao90163.1| +7	1.5e5	0.98	9882-10271
(K)	LPLVGQYQYDEGNKGTLKK (1)	gi|29541220|gb|aao90163.1| +7	2.3e4	0.98	9187

(K) SCKLPLVGQYQYDEGNK (1)	gi|29541220|gb|aao90163.1| +7	7.8e3	0.97	9935
(K) SCKLPLVGQYQYDEGNKGTLK (3)	gi|29541220|gb|aao90163.1| +7	1.2e5	0.93	9674-9954
(K) SCKLPLVGQYQYDEGNKGTLKK (3)	gi|29541220|gb|aao90163.1| +7	4.8e4	0.83	9063-9293

U gi|29541965|gb|AAO90903.1| **MS/MS Spectra:** 39 **Sum TIC:** 9.8e5 **Avg TIC:** 2.5e4 **Cov:** 42.7% **Uniq:** 10
hypothetical exported protein [Coxiella burnetii RSA 493]

(P1) **Sequence (count)**	**Reference (+addnl)**	**Σ TIC**	**Sf_{max}**	**Scans**
(K) GPFCSYF (2)	gi|29541965|gb|aao90903.1| +9	2.3e4	0.74	11900-11930
(R) LNCLYPVIK (4)	gi|29541965|gb|aao90903.1| +9	6.0e4	0.92	9743-10945
(R) LNCLYPVIKK (2)	gi|29541965|gb|aao90903.1| +9	4.8e4	0.91	8472-9211
(C) LYPVIKK (1)	gi|29541965|gb|aao90903.1| +9	1.5e4	0.64	6024
(K) NYISWAAGK (5)	gi|29541965|gb|aao90903.1| +9	2.6e5	0.93	9195-10172
(K) QKNYISWAAGK (1)	gi|29541965|gb|aao90903.1| +9	3.2e4	0.80	8478
(R) QSSNDCAR (2)	gi|29541965|gb|aao90903.1| +9	8.7e3	0.35	1459-2623
(K) YADDDLTGR (14)	gi|29541965|gb|aao90903.1| +9	2.1e5	0.96	4807-7012
(K) YADDDLTGRYR (5)	gi|29541965|gb|aao90903.1| +9	2.9e5	0.76	6129-7638
(R) YRLNCLYPVIK (3)	gi|29541965|gb|aao90903.1| +9	3.6e4	0.80	10213-10393

V uc|254622|bbs|112352 **MS/MS Spectra:** 60 **Sum TIC:** 8.9e5 **Avg TIC:** 1.5e4 **Cov:** 50.5% **Uniq:** 37
CON keratin 2, CK 2 [human, epidermis, Peptide, 645 aa] [Homo sapiens]

(P1) **Sequence (count)**	**Reference (+addnl)**	**Σ TIC**	**Sf_{max}**	**Scans**
(V) AGGGGGFGAAGGFGGR (1)	uc|254622|bbs|112352 +1	9.4e3	0.94	8598
(K) AQEREQIK (3)	uc|254622|bbs|112352 +5	1.8e4	0.81	3733-4064
(K) AQYEEIAQR (4)	uc|254622|bbs|112352 +12	5.2e4	0.95	6048-6798
(K) DVDNAYM*IK (1)	uc|254622|bbs|112352 +1	9.1e3	0.92	7469
(R) DYQELM*NVK (1)	uc|254622|bbs|112352 +5	8.3e3	0.93	7557
(R) GFSSGSAVVSGGSR (3)	uc|254622|bbs|112352 +1	5.4e4	0.98	6545-6931
(R) GGSGGGGSISGGGYGSGGGSGGR (1)	uc|254622|bbs|112352 +1	2.7e4	0.94	5659
(K) GGSISGGGYGSGGGK (2)	uc|254622|bbs|112352 +1	1.3e4	0.93	5224-5453
(K) GSSGEAFG (1)	uc|254622|bbs|112352 +1	7.5e3	0.16	6381
(R) GSSSGGGYSSGSSSYGSGGR (1)	uc|254622|bbs|112352 +1	3.1e4	0.98	5421
(R) HGGGGGGFGGGGFGSR (4)	uc|254622|bbs|112352 +1	7.3e4	0.98	6797-7513
(V) KVDPEIQNVK (1)	uc|254622|bbs|112352 +1	8.7e3	0.70	6224
(K) LLEGEECR (1)	uc|254622|bbs|112352 +15	9.9e3	0.79	6236
(R) LLRDYQELM*NVK (1)	uc|254622|bbs|112352 +5	1.9e4	0.80	8903
(R) LQGEIAHVK (1)	uc|254622|bbs|112352 +1	8.1e3	0.32	6053
(R) LQGEIAHVKK (1)	uc|254622|bbs|112352 +1	4.8e3	0.36	5406
(R) NKLNDLEEALQQAK (1)	uc|254622|bbs|112352 +1	4.2e3	0.89	11148
(R) NLDLDSIIAEVK (1)	uc|254622|bbs|112352 +14	6.3e3	0.65	11367
(L) NVKVDPEIQNVK (2)	uc|254622|bbs|112352 +1	9.3e3	0.68	6890-7317
(K) NVQDAIADAEQR (2)	uc|254622|bbs|112352 +1	2.8e4	0.97	8684-8693
(R) RSTSSFSCLSR (1)	uc|254622|bbs|112352 +1	8.2e3	0.45	7299
(R) SLVGLGGTK (1)	uc|254622|bbs|112352 +1	6.2e3	0.31	8369
(F) SSGSAVVSGGSR (1)	uc|254622|bbs|112352 +1	4.5e3	0.90	4696
(T) SSTISSNVASK (2)	uc|254622|bbs|112352 +1	1.4e4	0.52	4058-5361
(R) STSSFSCLSR (3)	uc|254622|bbs|112352 +1	1.6e4	0.92	7081-7846
(I) SVAGGGGGFGAAGGFGGR (1)	uc|254622|bbs|112352 +1	1.2e4	0.61	9001
(R) TAAENDFVTLK (1)	uc|254622|bbs|112352 +1	2.7e4	0.93	9336
(R) TAAENDFVTLKK (4)	uc|254622|bbs|112352 +1	8.9e4	0.94	8093-8317
(K) TLNNKFASFIDKVR (1)	uc|254622|bbs|112352 +22	1.3e4	0.95	10938
(R) TSQNSELNNM*QDLVEDYK (1)	uc|254622|bbs|112352 +1	5.1e3	0.94	8871
(R) TSQNSELNNM*QDLVEDYKK (1)	uc|254622|bbs|112352 +1	1.4e4	0.88	8103
(R) TSQNSELNNMQDLVEDYKK (1)	uc|254622|bbs|112352 +1	4.1e4	0.92	10741
(S) VAGGGGGFGAAGGFGGR (1)	uc|254622|bbs|112352 +1	8.6e3	0.82	8831
(K) VDPEIQNVK (2)	uc|254622|bbs|112352 +1	1.4e4	0.26	6083-6744
(N) VKVDPEIQNVK (2)	uc|254622|bbs|112352 +1	1.7e4	0.41	7197-7258
(R) YGSGGGSKGGSISGGGYGSGGGK (1)	uc|254622|bbs|112352 +1	8.7e3	0.98	5657
(R) YGSGGGSKGGSISGGGYGSGGGKHSSGGGSRGGSSS (1)	uc|254622|bbs|112352 +1	1.3e5	0.20	11508
(R) YLDGLTAER (2)	uc|254622|bbs|112352 +1	5.6e4	0.95	8886-8975

W gi|206583821|gb|AAO89868.2| **MS/MS Spectra:** 76 **Sum TIC:** 8.8e5 **Avg TIC:** 1.2e4 **Cov:** 65.1% **Uniq:** 44
outer membrane porin P1 [Coxiella burnetii RSA 493]

(P1) **Sequence (count)**	**Reference (+addnl)**	**Σ TIC**	**Sf_{max}**	**Scans**
(T) AIGVSGTVQNPK (1)	gi|206583821|gb|aao89868.2| +9	7.5e3	0.91	5309
(T) AIGVSGTVQNPKR (1)	gi|206583821|gb|aao89868.2| +9	4.6e3	0.88	5004
(D) APLVGSNYSYR (1)	gi|206583821|gb|aao89868.2| +7	5.0e3	0.79	6758
(K) AQYQYDNVR (8)	gi|206583821|gb|aao89868.2| +17	1.2e5	0.94	5096-7055
(M) DAPLVGSNYSYR (3)	gi|206583821|gb|aao89868.2| +7	6.8e4	0.95	6521-6997
(M) DAPLVGSNYSYRTR (2)	gi|206583821|gb|aao89868.2| +6	3.8e4	0.23	6601-6629
(Y) DYALYR (1)	gi|206583821|gb|aao89868.2| +17	6.3e3	0.47	7289
(F) HIGLGFGYK (1)	gi|206583821|gb|aao89868.2| +17	6.1e3	0.53	9339
(R) HYFM*N (1)	gi|206583821|gb|aao89868.2| +17	3.7e3	0.55	5080
(R) HYFMNNV (1)	gi|206583821|gb|aao89868.2| +17	6.8e3	0.76	7842
(T) LFGPGPVAVSM*K (1)	gi|206583821|gb|aao89868.2| +14	1.3e4	0.90	7789
(R) LGSHLTAM* (2)	gi|206583821|gb|aao89868.2| +16	1.5e4	0.53	3954-4662
(R) LGSHLTAM*L (1)	gi|206583821|gb|aao89868.2| +16	8.9e3	0.15	7501
(R) LGSHLTAM*LL (1)	gi|206583821|gb|aao89868.2| +16	3.8e3	0.19	9409
(R) LGSHLTAM*LLA (1)	gi|206583821|gb|aao89868.2| +16	5.3e3	0.11	9995
(R) LGSHLTAM*LLAGIK (1)	gi|206583821|gb|aao89868.2| +16	4.4e3	0.59	9950

(R)	LGSHLTAML (1)	gi|206583821|gb|aao89868.2| +16	1.1e4	0.41	9298
(R)	LGSHLTAMLL (1)	gi|206583821|gb|aao89868.2| +16	4.9e3	0.14	11307
(R)	LGSHLTAMLLA (1)	gi|206583821|gb|aao89868.2| +16	6.0e3	0.29	10913
(R)	LNGGIAGIGWR (1)	gi|206583821|gb|aao89868.2| +17	3.4e4	0.91	9383
(R)	LNGGIAGIGWRH (1)	gi|206583821|gb|aao89868.2| +17	7.6e3	0.84	8355
(A)	SAEGTAIGVSGTVQNPK (1)	gi|206583821|gb|aao89868.2| +7	2.5e3	0.88	5934
(S)	SATASAEGTAIGVSGTVQNPK (3)	gi|206583821|gb|aao89868.2| +7	3.1e4	0.98	6890-8201
(A)	SDWWIAGVK (4)	gi|206583821|gb|aao89868.2| +17	2.3e4	0.92	10596-12684
(R)	SKSNSVTLSSATASAEGTAIGV (1)	gi|206583821|gb|aao89868.2| +7	3.7e3	0.48	8268
(K)	SNSVTLSSATASAEGTAIGV (1)	gi|206583821|gb|aao89868.2| +7	1.0e4	0.97	9903
(G)	SNYSYR (1)	gi|206583821|gb|aao89868.2| +9	2.1e3	0.18	3641
(G)	SNYSYRTR (1)	gi|206583821|gb|aao89868.2| +8	4.5e3	0.14	4130
(R)	SVHIM*DAPLVGSN (1)	gi|206583821|gb|aao89868.2| +7	4.4e3	0.15	7219
(R)	SVHIM*DAPLVGSNYS (2)	gi|206583821|gb|aao89868.2| +7	9.5e3	0.32	7861-8771
(R)	SVHIM*DAPLVGSNYSYR (3)	gi|206583821|gb|aao89868.2| +7	4.4e4	0.97	7858-7897
(L)	SYDYALYR (2)	gi|206583821|gb|aao89868.2| +17	1.1e4	0.82	8268-8294
(K)	SYTYDQV (1)	gi|206583821|gb|aao89868.2| +15	6.3e3	0.55	6472
(K)	SYTYDQVGTVT (1)	gi|206583821|gb|aao89868.2| +15	3.6e3	0.72	6944
(K)	SYTYDQVGTVTV (4)	gi|206583821|gb|aao89868.2| +15	1.6e5	0.97	8445-10735
(K)	SYTYDQVGTVTVTT (1)	gi|206583821|gb|aao89868.2| +13	2.0e4	0.94	10089
(T)	TLFGPGPVAVSM*K (1)	gi|206583821|gb|aao89868.2| +14	6.8e3	0.72	8455
(T)	TLFGPGPVAVSMK (1)	gi|206583821|gb|aao89868.2| +14	4.9e3	0.41	9703
(R)	TRLGSHLTA (1)	gi|206583821|gb|aao89868.2| +14	1.1e4	0.16	5218
(R)	TRLGSHLTAM* (1)	gi|206583821|gb|aao89868.2| +14	4.4e3	0.58	5018
(R)	TRLGSHLTAM*L (2)	gi|206583821|gb|aao89868.2| +14	2.3e4	0.82	7285-7366
(R)	TRLGSHLTAML (1)	gi|206583821|gb|aao89868.2| +14	1.2e4	0.54	9007
(K)	TTLFGPGPVA (2)	gi|206583821|gb|aao89868.2| +15	1.2e4	0.28	9642-9668
(K)	TTLFGPGPVAV (1)	gi|206583821|gb|aao89868.2| +15	3.0e3	0.38	10787
(K)	TTLFGPGPVAVSM*K (1)	gi|206583821|gb|aao89868.2| +14	6.6e3	0.87	8594
(K)	TTLFGPGPVAVSMK (2)	gi|206583821|gb|aao89868.2| +14	2.0e4	0.93	9840-10808
(L)	VGSNYSYR (1)	gi|206583821|gb|aao89868.2| +9	8.1e3	0.52	4780
(K)	VNEANAVYLEA (1)	gi|206583821|gb|aao89868.2| +17	1.0e4	0.53	10182
(A)	VYLEAGYSTVWGK (1)	gi|206583821|gb|aao89868.2| +17	4.9e3	0.95	10524
(Q)	YQYDNVR (1)	gi|206583821|gb|aao89868.2| +17	3.4e4	0.42	6784

X gi|29542467|gb|AAO91401.1| **MS/MS Spectra:** 38 **Sum TIC:** 6.5e5 **Avg TIC:** 1.7e4 **Cov:** 44.0% **Uniq:** 16
outer membrane protein [Coxiella burnetii RSA 493]

(P1)	**Sequence (count)**	**Reference (+addnl)**	**Σ TIC**	**Sf_{max}**	**Scans**
(K)	AM*NSVIQAIVK (4)	gi|29542467|gb|aao91401.1| +39	4.6e4	0.96	8366-9796
(K)	AM*NSVIQAIVKQNK (1)	gi|29542467|gb|aao91401.1| +39	9.9e3	0.45	7877
(K)	AMNSVIQAIVK (2)	gi|29542467|gb|aao91401.1| +39	3.3e4	0.96	10038-11315
(K)	AMNSVIQAIVKQNK (1)	gi|29542467|gb|aao91401.1| +39	7.2e3	0.71	9394
(A)	APSQFSFSPQQVK (3)	gi|29542467|gb|aao91401.1| +22	2.4e4	0.97	8940-9315
(K)	DM*DNPAIQK (4)	gi|29542467|gb|aao91401.1| +42	5.7e4	0.91	5002-6151
(K)	DMDNPAIQK (1)	gi|29542467|gb|aao91401.1| +42	7.8e3	0.90	6001
(V)	EFFDYQCGHCK (1)	gi|29542467|gb|aao91401.1| +44	4.4e3	0.65	7504
(K)	EIDRVEK (3)	gi|29542467|gb|aao91401.1| +35	2.6e5	0.83	4525-4560
(K)	FGFIPGATSQQNLQK (3)	gi|29542467|gb|aao91401.1| +39	6.8e4	0.97	10294-10900
(K)	KDM*DNPAIQK (3)	gi|29542467|gb|aao91401.1| +42	1.8e4	0.89	4523-4587
(K)	KLFNDPASPVAGN (1)	gi|29542467|gb|aao91401.1| +38	6.4e3	0.81	8516
(K)	KTEAQQEEHAQQAIK (2)	gi|29542467|gb|aao91401.1| +41	2.3e4	0.97	5012-5043
(K)	KTEAQQEEHAQQAIKENAK (2)	gi|29542467|gb|aao91401.1| +41	3.4e4	0.91	5535-5543
(P)	SQFSFSPQQVK (1)	gi|29542467|gb|aao91401.1| +22	7.1e3	0.64	9182
(K)	TEAQQEEHAQQAIK (1)	gi|29542467|gb|aao91401.1| +41	4.7e3	0.93	5447
(K)	TEAQQEEHAQQAIKENAK (1)	gi|29542467|gb|aao91401.1| +41	4.2e3	0.71	6084
(K)	VGLNVAQLK (2)	gi|29542467|gb|aao91401.1| +42	1.5e4	0.90	8996-9528
(K)	VGLNVAQLKK (2)	gi|29542467|gb|aao91401.1| +42	2.0e4	0.93	7894-8365

Y gi|206584120|gb|AAO91214.2| **MS/MS Spectra:** 23 **Sum TIC:** 6.3e5 **Avg TIC:** 2.7e4 **Cov:** 73.3% **Uniq:** 8
10 kDa chaperonin GROES [Coxiella burnetii RSA 493]

(P1)	**Sequence (count)**	**Reference (+addnl)**	**Σ TIC**	**Sf_{max}**	**Scans**
(S)	AGGIVIPDSAAEKPSR (1)	gi|206584120|gb|aao91214.2| +17	3.1e3	0.11	6896
(R)	GEVISVGPGKPLDNGEVR (1)	gi|206584120|gb|aao91214.2| +17	3.1e4	0.83	8909
(K)	IRPLHDR (2)	gi|206584120|gb|aao91214.2| +17	9.8e3	0.29	4796-4960
(K)	LAGDEYIVM*REDDIM*GVIEK (1)	gi|206584120|gb|aao91214.2| +17	8.4e4	0.96	11495
(R)	RLEEER (6)	gi|206584120|gb|aao91214.2| +17	8.0e4	0.87	4044-4222
(R)	TSAGGIVIPDSAAEKPSR (3)	gi|206584120|gb|aao91214.2| +17	1.8e4	0.87	8015-8472
(K)	VGDQILFGK (7)	gi|206584120|gb|aao91214.2| +17	3.4e5	0.97	9814-10606
(K)	YAGTEVK (2)	gi|206584120|gb|aao91214.2| +17	5.5e4	0.63	4815-5082

Z gi|206584103|gb|AAO91139.2| **MS/MS Spectra:** 28 **Sum TIC:** 5.9e5 **Avg TIC:** 2.1e4 **Cov:** 36.1% **Uniq:** 18
DotD [Coxiella burnetii RSA 493]

(P1)	**Sequence (count)**	**Reference (+addnl)**	**Σ TIC**	**Sf_{max}**	**Scans**
(K)	ANITLYPSR (1)	gi|206584103|gb|aao91139.2| +13	7.9e3	0.88	7876
(K)	ANITLYPSRR (3)	gi|206584103|gb|aao91139.2| +13	2.8e4	0.28	6667-7238
(V)	EKKANITLYPSR (1)	gi|206584103|gb|aao91139.2| +13	3.4e3	0.12	6137
(A)	INSTDVPLAEILR (2)	gi|206584103|gb|aao91139.2| +13	8.7e3	0.84	11270-12254
(K)	KANITLYPSR (1)	gi|206584103|gb|aao91139.2| +13	1.0e4	0.91	7061
(K)	KANITLYPSRR (1)	gi|206584103|gb|aao91139.2| +13	1.8e4	0.63	5994

(R)	KIASASHYR (1)	gi|206584103|gb|aao91139.2| +13	3.4e3	0.84	3482
(K)	KPAIPVL (2)	gi|206584103|gb|aao91139.2| +13	4.9e4	0.75	8888-8947
(K)	KPAIPVLVA (3)	gi|206584103|gb|aao91139.2| +13	2.7e4	0.76	9166-9350
(K)	KPAIPVLVAI (1)	gi|206584103|gb|aao91139.2| +13	7.5e3	0.53	11630
(R)	LRVLGKKPAIPVLV (1)	gi|206584103|gb|aao91139.2| +13	3.3e3	0.69	9719
(R)	NATYQVEK (1)	gi|206584103|gb|aao91139.2| +13	2.4e3	0.11	4227
(K)	PAIPVLVA (1)	gi|206584103|gb|aao91139.2| +13	5.0e3	0.27	10407
(R)	RIIELR (1)	gi|206584103|gb|aao91139.2| +13	9.6e3	0.62	6285
(A)	TYQVEK (3)	gi|206584103|gb|aao91139.2| +13	1.3e4	0.74	3856-3874
(R)	VLGKKPAIPVL (2)	gi|206584103|gb|aao91139.2| +13	2.7e4	0.90	8660-8765
(R)	VLGKKPAIPVLVA (2)	gi|206584103|gb|aao91139.2| +13	3.7e5	0.90	9084-9085
(R)	VLGKKPAIPVLVAI (1)	gi|206584103|gb|aao91139.2| +13	4.3e3	0.81	11186

a gi|206583792|gb|AAO89740.2| **MS/MS Spectra:** 41 **Sum TIC:** 5.6e5 **Avg TIC:** 1.4e4 **Cov:** 42.7% **Uniq:** 32
peptidoglycan-specific endopeptidase, M23 family [Coxiella burnetii RSA 493]

(P1)	**Sequence (count)**	**Reference (+addnl)**	**Σ TIC**	**Sf_{max}**	**Scans**
(A)	ALKYPLSSAK (4)	gi|206583792|gb|aao89740.2| +13	1.5e5	0.90	6431-6492
(A)	ALKYPLSSAKTLIIKR (1)	gi|206583792|gb|aao89740.2| +13	5.8e3	0.11	8096
(A)	ARFGTPVK (1)	gi|206583792|gb|aao89740.2| +13	2.1e4	0.52	5092
(R)	DGGYGRTVK (1)	gi|206583792|gb|aao89740.2| +13	5.0e3	0.18	3567
(K)	DLVQLAK (1)	gi|206583792|gb|aao89740.2| +13	7.3e3	0.70	6982
(R)	ENNRFISQIDQKPITTA (1)	gi|206583792|gb|aao89740.2| +13	8.5e3	0.52	7829
(R)	FGTPVKSIGEGR (1)	gi|206583792|gb|aao89740.2| +13	5.1e3	0.21	6081
(V)	FIGRDGGYGR (1)	gi|206583792|gb|aao89740.2| +13	5.5e3	0.57	5329
(R)	FISQIDQKPITTA (1)	gi|206583792|gb|aao89740.2| +13	2.8e4	0.80	8211
(R)	FLHAPLH (2)	gi|206583792|gb|aao89740.2| +7	1.1e4	0.41	6536-6615
(R)	FTYHRLDPILH (2)	gi|206583792|gb|aao89740.2| +13	2.0e4	0.62	8126-8143
(R)	HQWVHKGQIIG (2)	gi|206583792|gb|aao89740.2| +13	8.6e4	0.90	6482-6559
(K)	INQKDLVQLAKK (1)	gi|206583792|gb|aao89740.2| +3	3.7e3	0.58	6783
(K)	IRPHLGVDFA (1)	gi|206583792|gb|aao89740.2| +13	4.2e3	0.24	8501
(K)	ISYGHHY (1)	gi|206583792|gb|aao89740.2| +13	2.1e4	0.82	4713
(I)	KKGDTLAAIFNR (1)	gi|206583792|gb|aao89740.2| +13	3.6e3	0.90	8293
(R)	LDPILH (1)	gi|206583792|gb|aao89740.2| +13	5.0e3	0.16	6752
(R)	LKINQKDL (1)	gi|206583792|gb|aao89740.2| +13	8.0e3	0.73	6187
(R)	LKINQKDLVQLAK (1)	gi|206583792|gb|aao89740.2| +13	6.2e3	0.84	7763
(T)	LNEPHSITIVK (1)	gi|206583792|gb|aao89740.2| +13	8.9e3	0.87	6583
(A)	LYGHLSR (3)	gi|206583792|gb|aao89740.2| +13	5.1e4	0.83	5000-5055
(A)	LYGHLSRFAK (1)	gi|206583792|gb|aao89740.2| +13	7.9e3	0.69	6426
(K)	NIDNSNSDWKK (1)	gi|206583792|gb|aao89740.2| +13	4.2e3	0.80	4457
(V)	QLAKKYK (1)	gi|206583792|gb|aao89740.2| +3	5.5e3	0.22	8586
(K)	SIGEGRVVF (1)	gi|206583792|gb|aao89740.2| +13	4.4e3	0.10	8490
(K)	SIGEGRVVFIGR (1)	gi|206583792|gb|aao89740.2| +13	1.5e4	0.66	8048
(S)	SRFTYHR (1)	gi|206583792|gb|aao89740.2| +13	6.8e3	0.18	4684
(K)	TLIIKRENNR (2)	gi|206583792|gb|aao89740.2| +13	2.0e4	0.60	4678-4710
(R)	TVKISYGHHYLA (1)	gi|206583792|gb|aao89740.2| +13	8.4e3	0.22	6786
(R)	VVFIGRDGGYGR (1)	gi|206583792|gb|aao89740.2| +13	8.2e3	0.58	7092
(R)	YTYPIAHTA (1)	gi|206583792|gb|aao89740.2| +13	7.2e3	0.42	6443
(A)	YYTPDGRGIEAR (1)	gi|206583792|gb|aao89740.2| +13	4.2e3	0.21	5947

b gi|161762213|gb|ABX77855.1| **MS/MS Spectra:** 29 **Sum TIC:** 5.0e5 **Avg TIC:** 1.7e4 **Cov:** 73.3% **Uniq:** 13
hypothetical protein COXBURSA331_A2077 [Coxiella burnetii RSA 331]

(P1)	**Sequence (count)**	**Reference (+addnl)**	**Σ TIC**	**Sf_{max}**	**Scans**
(R)	AKWSHLGPGQVCCQD (1)	gi|161762213|gb|abx77855.1| +1	7.2e3	0.94	7125
(R)	AKWSHLGPGQVCCQDPK (4)	gi|161762213|gb|abx77855.1| +1	1.1e5	0.95	6602-6738
(R)	AKWSHLGPGQVCCQDPKVPSIQCR (1)	gi|161762213|gb|abx77855.1| +1	6.6e3	0.71	7925
(G)	CAMESNSGK (1)	gi|161762213|gb|abx77855.1| +1	8.4e3	0.27	3854
(K)	CLCLLGTSFTLGPNCK (2)	gi|161762213|gb|abx77855.1| +1	3.3e4	0.98	12276-12320
(G)	EICPTGCAM*ESNSGK (5)	gi|161762213|gb|abx77855.1| +1	5.0e4	0.93	1827-5559
(G)	EICPTGCAMESNSGK (2)	gi|161762213|gb|abx77855.1| +1	6.4e4	0.93	5539-5547
(K)	EVICGK (1)	gi|161762213|gb|abx77855.1| +1	1.2e4	0.60	3508
(K)	HYYYCSR (2)	gi|161762213|gb|abx77855.1| +1	1.6e4	0.92	4638-5301
(V)	PSIQCR (1)	gi|161762213|gb|abx77855.1| +1	1.0e4	0.56	4893
(K)	TSQDLKEVICGK (4)	gi|161762213|gb|abx77855.1| +1	1.5e5	0.93	7514-8555
(K)	TSQDLKEVICGKHYYYCSR (1)	gi|161762213|gb|abx77855.1| +1	5.6e3	0.75	8278
(K)	WSHLGPGQVCCQD (2)	gi|161762213|gb|abx77855.1| +1	2.6e4	0.96	7278-7332
(K)	WSHLGPGQVCCQDPK (2)	gi|161762213|gb|abx77855.1| +1	1.3e4	0.79	6725-6752

c gi|29542266|gb|AAO91203.1| **MS/MS Spectra:** 21 **Sum TIC:** 4.4e5 **Avg TIC:** 2.1e4 **Cov:** 42.0% **Uniq:** 10
superoxide dismutase [Coxiella burnetii RSA 493]

(P1)	**Sequence (count)**	**Reference (+addnl)**	**Σ TIC**	**Sf_{max}**	**Scans**
(M)	AFELPDLPYK (3)	gi|29542266|gb|aao91203.1| +16	7.8e4	0.93	11649-11972
(R)	AYVNKLNK (1)	gi|29542266|gb|aao91203.1| +32	1.7e4	0.77	5488
(K)	DNNGKLEVLSTVNAR (2)	gi|29542266|gb|aao91203.1| +16	6.6e4	0.97	8611-8649
(K)	EPLEEIIR (1)	gi|29542266|gb|aao91203.1| +31	4.0e3	0.11	9905
(K)	LEVLSTVNAR (1)	gi|29542266|gb|aao91203.1| +16	8.6e3	0.96	8631
(K)	LIEGTPFEK (1)	gi|29542266|gb|aao91203.1| +32	1.6e4	0.56	8764
(K)	LIEGTPFEKEPLEEIIR (4)	gi|29542266|gb|aao91203.1| +31	7.7e4	0.95	11781-12626
(K)	LIEGTPFEKEPLEEIIRK (4)	gi|29542266|gb|aao91203.1| +31	4.8e4	0.85	10974-11789
(K)	LNALEPHISQETLEYHHGK (3)	gi|29542266|gb|aao91203.1| +28	1.2e5	0.90	8421-8488

(P1)	Sequence (count)	Reference (+addnl)	Σ TIC	Sf_{max}	Scans
(D)	PSGELASAIDK (1)	gi|29542266|gb|aao91203.1| +16	5.6e3	0.79	6681

d uc|P13647|K2C5_HUMAN **MS/MS Spectra:** 39 **Sum TIC:** 3.6e5 **Avg TIC:** 9.3e3 **Cov:** 32.4% **Uniq:** 20
CON Keratin, type II cytoskeletal 5 OS=Homo sapiens GN=KRT5 PE=1 SV=3

(P1)	Sequence (count)	Reference (+addnl)	Σ TIC	Sf_{max}	Scans
(K)	AQYEEIANR (3)	uc|p13647|k2c5_human +3	2.7e4	0.95	5991-6674
(R)	FLEQQNK (1)	uc|p13647|k2c5_human +39	5.0e3	0.66	5356
(R)	FLEQQNKVLDTK (1)	uc|p13647|k2c5_human +7	6.8e3	0.88	7943
(R)	GLGVGFGSGGGSSSSVK (1)	uc|p13647|k2c5_human +3	9.3e3	0.93	8815
(R)	GRLDSELR (2)	uc|p13647|k2c5_human +12	1.5e4	0.76	5958-6631
(R)	HGDDLRNTK (2)	uc|p13647|k2c5_human +16	1.3e4	0.83	3832-3861
(R)	ISISTSGGSFR (3)	uc|p13647|k2c5_human +1	4.3e4	0.94	7884-8705
(R)	NTKHEISEM*NR (1)	uc|p13647|k2c5_human +2	4.6e3	0.57	4102
(R)	NTKHEISEMNR (1)	uc|p13647|k2c5_human +2	8.1e3	0.72	5283
(R)	QLDSIVGER (3)	uc|p13647|k2c5_human +10	3.7e4	0.59	7388-8246
(R)	SGGGGGGGFGR (3)	uc|p13647|k2c5_human +1	3.1e4	0.91	5040-5299
(R)	SLYNLGGSK (1)	uc|p13647|k2c5_human +2	6.8e3	0.11	8560
(R)	SLYNLGGSKR (1)	uc|p13647|k2c5_human +2	1.1e4	0.48	7772
(R)	SRTEAESWYQTK (1)	uc|p13647|k2c5_human +3	3.8e3	0.82	7748
(R)	TEEREQIK (1)	uc|p13647|k2c5_human +5	5.3e3	0.29	3906
(R)	TSFTSVSR (2)	uc|p13647|k2c5_human +1	1.5e4	0.73	6657-6689
(R)	TTAENEFVM*LKK (1)	uc|p13647|k2c5_human +1	1.4e4	0.71	8441
(K)	VDALM*DEINFM*K (2)	uc|p13647|k2c5_human +3	1.1e4	0.93	9329-10040
(R)	VSLAGACGVGGYGSR (2)	uc|p13647|k2c5_human +2	2.1e4	0.97	8437-8518
(K)	WTLLQEQGTK (2)	uc|p13647|k2c5_human +11	2.8e4	0.93	10239-10794
(K)	YEELQQTAGR (5)	uc|p13647|k2c5_human +3	4.8e4	0.96	5728-6679

e gi|206584148|gb|AAO91315.2| **MS/MS Spectra:** 20 **Sum TIC:** 3.4e5 **Avg TIC:** 1.7e4 **Cov:** 56.6% **Uniq:** 13
superoxide dismutase (Cu-Zn) [Coxiella burnetii RSA 493]

(P1)	Sequence (count)	Reference (+addnl)	Σ TIC	Sf_{max}	Scans
(T)	DTQYGLLIKPNLR (1)	gi|206584148|gb|aao91315.2| +11	1.6e4	0.93	9010
(A)	ETVAVNM*YR (3)	gi|206584148|gb|aao91315.2| +11	1.7e4	0.62	5342-6490
(A)	ETVAVNMYR (1)	gi|206584148|gb|aao91315.2| +11	1.1e4	0.85	7215
(K)	GEAAGGHLDPANTGK (2)	gi|206584148|gb|aao91315.2| +11	1.5e4	0.75	4204-4218
(K)	HLGPYNPNG (1)	gi|206584148|gb|aao91315.2| +11	9.7e3	0.25	7081
(R)	IACGIVK (1)	gi|206584148|gb|aao91315.2| +11	7.0e3	0.21	6818
(R)	IHGFHIHVNPDCSNK (1)	gi|206584148|gb|aao91315.2| +3	9.4e3	0.58	8003
(R)	IHGFHIHVNPDCSNKGEAAGGHLDPANTGK (1)	gi|206584148|gb|aao91315.2| +3	7.9e4	0.97	8082
(G)	LLIKPNLR (2)	gi|206584148|gb|aao91315.2| +11	1.7e4	0.48	7245-7407
(T)	LTDTQYGLLIKPNLR (1)	gi|206584148|gb|aao91315.2| +11	5.9e3	0.67	9269
(K)	NGTATLPILAPR (1)	gi|206584148|gb|aao91315.2| +11	4.9e3	0.79	10016
(K)	NLIGHSLM*IHAGGDNYSDHPK (2)	gi|206584148|gb|aao91315.2| +11	4.4e4	0.96	8684-8794
(K)	NLIGHSLM*IHAGGDNYSDHPKELGGGGAR (1)	gi|206584148|gb|aao91315.2| +9	9.4e4	0.94	8822
(K)	NLIGHSLMIHAGGDNYSDHPKELGGGGAR (1)	gi|206584148|gb|aao91315.2| +9	6.6e3	0.24	9311
(Q)	YGLLIKPNLR (1)	gi|206584148|gb|aao91315.2| +11	5.3e3	0.58	8839

f gi|29541760|gb|AAO90699.1| **MS/MS Spectra:** 25 **Sum TIC:** 3.2e5 **Avg TIC:** 1.3e4 **Cov:** 55.5% **Uniq:** 11
outer-membrane lipoproteins carrier protein [Coxiella burnetii RSA 493]

(P1)	Sequence (count)	Reference (+addnl)	Σ TIC	Sf_{max}	Scans
(K)	FNQITFDGQDR (2)	gi|29541760|gb|aao90699.1| +19	8.1e4	0.95	8682-8734
(R)	FRWETDSPTK (2)	gi|29541760|gb|aao90699.1| +19	5.9e4	0.92	8100-8153
(R)	GIDVVKQ (5)	gi|29541760|gb|aao90699.1| +19	3.8e4	0.80	5892-6323
(K)	QLTEM*TVLNNLDER (1)	gi|29541760|gb|aao90699.1| +19	1.2e4	0.97	10012
(K)	QLTEMTVLNNLDER (1)	gi|29541760|gb|aao90699.1| +19	8.6e3	0.97	11144
(D)	RVIQQSHGR (1)	gi|29541760|gb|aao90699.1| +19	2.9e3	0.34	3843
(R)	SIFQFSQIK (2)	gi|29541760|gb|aao90699.1| +19	8.4e3	0.88	11423-11743
(K)	SLNFNWIR (1)	gi|29541760|gb|aao90699.1| +19	3.6e3	0.78	12277
(K)	TLWVYDVDLSQATQQPLAQK (1)	gi|29541760|gb|aao90699.1| +19	2.3e3	0.97	12380
(K)	TNINPASLLSGSVK (5)	gi|29541760|gb|aao90699.1| +19	5.3e4	0.96	11027-11705
(K)	VNAPLSSTLFEFKPSR (3)	gi|29541760|gb|aao90699.1| +19	4.9e4	0.97	11562-11636
(R)	WETDSPTK (1)	gi|29541760|gb|aao90699.1| +19	6.1e3	0.44	5903

g gi|29542211|gb|AAO91148.1| **MS/MS Spectra:** 28 **Sum TIC:** 3.0e5 **Avg TIC:** 1.1e4 **Cov:** 37.0% **Uniq:** 19
IcmX [Coxiella burnetii RSA 493]

(P1)	Sequence (count)	Reference (+addnl)	Σ TIC	Sf_{max}	Scans
(K)	AIKEVGLEVQALAIASKK (1)	gi|29542211|gb|aao91148.1| +13	7.4e3	0.86	10999
(T)	ASPAVVAR (2)	gi|29542211|gb|aao91148.1| +13	2.9e4	0.66	3948-5513
(A)	DIDPM*SGVIK (1)	gi|29542211|gb|aao91148.1| +13	2.2e4	0.71	7702
(V)	ENYIANQR (1)	gi|29542211|gb|aao91148.1| +13	7.7e3	0.74	4632
(R)	EQVLILA (1)	gi|29542211|gb|aao91148.1| +13	1.4e4	0.38	10919
(N)	ERVPVKGLGAK (1)	gi|29542211|gb|aao91148.1| +11	7.8e3	0.72	4943
(K)	GYASPLQV (1)	gi|29542211|gb|aao91148.1| +13	7.0e3	0.64	8422
(K)	GYASPLQVENYIANQR (3)	gi|29542211|gb|aao91148.1| +13	5.8e4	0.98	11322-11629
(R)	INSPDWFK (1)	gi|29542211|gb|aao91148.1| +13	7.7e3	0.10	10842
(K)	LIDQIGK (3)	gi|29542211|gb|aao91148.1| +13	4.0e4	0.55	7329-7386
(N)	LNKLSAEK (1)	gi|29542211|gb|aao91148.1| +13	5.1e3	0.72	3808
(L)	NERVPVKGLGAK (1)	gi|29542211|gb|aao91148.1| +11	4.5e3	0.75	5084
(R)	NHLDNER (2)	gi|29542211|gb|aao91148.1| +13	2.0e4	0.79	3711-3819

(K)	NSEM*ELQTNTAADLNK (2)	gi|29542211|gb|aao91148.1| +13	2.3e4	0.98	7264-7477
(K)	SFLNNPAYQK (1)	gi|29542211|gb|aao91148.1| +9	6.9e3	0.43	7351
(K)	TASPAVVAR (2)	gi|29542211|gb|aao91148.1| +13	1.3e4	0.53	4319-5489
(K)	VGM*PDDPHLPKGYASPLQV (1)	gi|29542211|gb|aao91148.1| +13	5.4e3	0.77	8558
(K)	VGMPDDPHLPK (2)	gi|29542211|gb|aao91148.1| +13	2.1e4	0.77	7279-8092
(N)	YIANQR (1)	gi|29542211|gb|aao91148.1| +13	5.6e3	0.52	3434

h gi|29542078|gb|AAO91016.1| **MS/MS Spectra:** 27 **Sum TIC:** 2.9e5 **Avg TIC:** 1.1e4 **Cov:** 21.7% **Uniq:** 4
protein translocase subunit [Coxiella burnetii RSA 493]

(P1)	**Sequence (count)**	**Reference (+addnl)**	**Σ TIC**	**Sf_{max}**	**Scans**
(K)	DLSFEAPR (5)	gi|29542078|gb|aao91016.1| +18	3.1e4	0.85	8309-9160
(R)	EAITDM*VVR (8)	gi|29542078|gb|aao91016.1| +18	1.1e5	0.91	6807-7772
(R)	EAITDMVVR (3)	gi|29542078|gb|aao91016.1| +18	2.1e4	0.90	9289-9763
(R)	LYIKDLSFEAPR (3)	gi|29542078|gb|aao91016.1| +18	1.5e4	0.96	10501-11053
(R)	TNTPDNGPEFAIQR (8)	gi|29542078|gb|aao91016.1| +15	1.1e5	0.96	7878-8714

i uc|125080|sp|P02533|K1CN_HUMAN **MS/MS Spectra:** 24 **Sum TIC:** 2.9e5 **Avg TIC:** 1.2e4 **Cov:** 29.9% **Uniq:** 16
CON Keratin, type i cytoskeletal 14 (cytokeratin 14) (K14) (CK 14) gi|2144816|pir||KRHUE keratin, 50K type I cytoskeletal - human gi|386848 (J00124) keratin [Homo sapiens]

(P1)	**Sequence (count)**	**Reference (+addnl)**	**Σ TIC**	**Sf_{max}**	**Scans**
(R)	ALEEANADLEVK (1)	uc|125080|sp|p02533|k1cn_huma +9	5.2e4	0.96	8117
(K)	DAEEWFFTK (2)	uc|125080|sp|p02533|k1cn_huma	1.1e4	0.90	12307-12898
(R)	EVATNSELVQSGK (3)	uc|125080|sp|p02533|k1cn_huma +2	5.4e4	0.96	5887-6820
(R)	GQVGGDVNVEM*DAAPGVDLSR (1)	uc|125080|sp|p02533|k1cn_huma	4.7e4	0.97	9048
(R)	ILNEMRDQYEK (2)	uc|125080|sp|p02533|k1cn_huma +2	1.8e4	0.79	7577-7607
(K)	IRDWYQR (1)	uc|125080|sp|p02533|k1cn_huma +5	2.2e4	0.78	8046
(R)	LEQEIATYR (1)	uc|125080|sp|p02533|k1cn_huma +17	7.9e3	0.95	7623
(R)	LLEGEDAHLSSSQFSSGSQSSR (1)	uc|125080|sp|p02533|k1cn_huma +1	5.7e3	0.97	8423
(R)	RLLEGEDAHLSSSQFSSGSQSSR (1)	uc|125080|sp|p02533|k1cn_huma +1	3.1e3	0.13	8127
(S)	SVLAGGSCR (1)	uc|125080|sp|p02533|k1cn_huma +1	4.1e3	0.43	5620
(R)	TEELNR (1)	uc|125080|sp|p02533|k1cn_huma +4	9.5e3	0.34	4545
(K)	TEELNREVATNSELVQSGK (1)	uc|125080|sp|p02533|k1cn_huma +2	4.9e3	0.92	8154
(R)	TKYETELNLR (1)	uc|125080|sp|p02533|k1cn_huma	8.5e3	0.70	8208
(K)	TRLEQEIATYR (1)	uc|125080|sp|p02533|k1cn_huma +15	8.8e3	0.81	8947
(K)	TRLEQEIATYRR (1)	uc|125080|sp|p02533|k1cn_huma +12	6.4e3	0.73	7571
(R)	VLDELTLAR (5)	uc|125080|sp|p02533|k1cn_huma +10	2.8e4	0.94	9364-10133

j gi|206584030|gb|ACI15284.1| **MS/MS Spectra:** 8 **Sum TIC:** 2.8e5 **Avg TIC:** 3.5e4 **Cov:** 83.3% **Uniq:** 4
ScvA [Coxiella burnetii RSA 493]

(P1)	**Sequence (count)**	**Reference (+addnl)**	**Σ TIC**	**Sf_{max}**	**Scans**
(R)	GKDQRPQRPGASNPR (4)	gi|206584030|gb|aci15284.1| +9	1.6e5	0.66	2974-3311
(-)	MERQNVQQQR (2)	gi|206584030|gb|aci15284.1| +9	1.1e5	0.93	3117-3120
(R)	QNVQQQR (1)	gi|206584030|gb|aci15284.1| +9	6.9e3	0.41	2808
(E)	RQNVQQQR (1)	gi|206584030|gb|aci15284.1| +9	3.9e3	0.90	1499

k gi|29541523|gb|AAO90463.1| **MS/MS Spectra:** 32 **Sum TIC:** 2.6e5 **Avg TIC:** 8.0e3 **Cov:** 48.7% **Uniq:** 23
OmpA-like transmembrane domain protein [Coxiella burnetii RSA 493]

(P1)	**Sequence (count)**	**Reference (+addnl)**	**Σ TIC**	**Sf_{max}**	**Scans**
(K)	AGGGYQFNK (2)	gi|29541523|gb|aao90463.1| +11	9.5e3	0.90	5313-5354
(K)	AGGGYQFNKYFALEG (1)	gi|29541523|gb|aao90463.1| +11	5.1e3	0.80	10551
(K)	AIIPLKKGYNVFAK (1)	gi|29541523|gb|aao90463.1| +13	1.6e4	0.86	8797
(T)	EIYAAGK (1)	gi|29541523|gb|aao90463.1| +13	3.0e3	0.34	4159
(P)	EKSNTEIYAAGK (3)	gi|29541523|gb|aao90463.1| +11	2.0e4	0.69	4305-4555
(A)	GKAIIPLKK (1)	gi|29541523|gb|aao90463.1| +13	6.2e3	0.25	5554
(L)	GSIDDGGIIYK (2)	gi|29541523|gb|aao90463.1| +11	1.9e4	0.64	7056-7854
(K)	GYNVFAK (1)	gi|29541523|gb|aao90463.1| +13	8.5e3	0.83	7251
(K)	GYNVFAKIGAAQV (1)	gi|29541523|gb|aao90463.1| +13	4.1e3	0.77	10108
(K)	KGYNVFAK (2)	gi|29541523|gb|aao90463.1| +13	3.6e4	0.48	6949-6953
(A)	LEGNYTR (1)	gi|29541523|gb|aao90463.1| +11	6.9e3	0.50	4017
(A)	LEGNYTRFPNLK (1)	gi|29541523|gb|aao90463.1| +11	6.1e3	0.43	7960
(A)	LEGNYTRFPNLKSGR (2)	gi|29541523|gb|aao90463.1| +11	1.2e4	0.63	7234-7265
(G)	LYTNGDLVGVKPK (1)	gi|29541523|gb|aao90463.1| +11	4.2e3	0.59	6827
(T)	NLGSIDDGGIIYK (1)	gi|29541523|gb|aao90463.1| +11	4.6e3	0.61	8292
(S)	NTEIYAAGK (1)	gi|29541523|gb|aao90463.1| +13	2.5e3	0.16	4858
(G)	NYTRFPNLK (1)	gi|29541523|gb|aao90463.1| +11	4.2e3	0.18	7981
(G)	NYTRFPNLKSGR (2)	gi|29541523|gb|aao90463.1| +11	3.3e4	0.83	6591-6599
(E)	SGLYTNGDLVGVKPK (1)	gi|29541523|gb|aao90463.1| +11	4.9e3	0.83	7301
(R)	VIPEKSNTEIYA (2)	gi|29541523|gb|aao90463.1| +11	2.1e4	0.50	6396-6397
(K)	VTNLGSIDDGGIIYK (2)	gi|29541523|gb|aao90463.1| +11	8.5e3	0.96	8416-9350
(K)	YFALEGNYTR (1)	gi|29541523|gb|aao90463.1| +11	4.1e3	0.23	8442
(N)	YTRFPNLK (1)	gi|29541523|gb|aao90463.1| +11	1.4e4	0.44	6958

l gi|206583875|gb|AAO90078.2| **MS/MS Spectra:** 16 **Sum TIC:** 2.5e5 **Avg TIC:** 1.6e4 **Cov:** 50.4% **Uniq:** 7
COME operon protein 1 [Coxiella burnetii RSA 493]

(P1)	**Sequence (count)**	**Reference (+addnl)**	**Σ TIC**	**Sf_{max}**	**Scans**
(A)	DATLLTTLKGIGVKK (1)	gi|206583875|gb|aao90078.2| +13	6.7e3	0.68	9897

(I)	EALSSVPGISQK (1)	gi|206583875|gb|aao90078.2| +13	6.0e3	0.51	6864
(R)	HAAVSTQK (4)	gi|206583875|gb|aao90078.2| +13	1.4e4	0.95	1472-3175
(A)	KPASEVIK (2)	gi|206583875|gb|aao90078.2| +13	3.7e4	0.68	5157-5186
(R)	LIRNNPHR (1)	gi|206583875|gb|aao90078.2| +13	7.4e3	0.26	3260
(K)	SIEALSSVPGISQK (6)	gi|206583875|gb|aao90078.2| +13	1.7e5	0.96	8528-10679
(K)	TGPVDINTADATLLTTLK (1)	gi|206583875|gb|aao90078.2| +13	8.0e3	0.94	13047

m gi|29542079|gb|AAO91017.1| **MS / MS Spectra:** 13 **Sum TIC:** 2.4e5 **Avg TIC:** 1.8e4 **Cov:** 83.5% **Uniq:** 9
glutaredoxin [Coxiella burnetii RSA 493]

(P1)	Sequence (count)	Reference (+addnl)	Σ TIC	Sf_{max}	Scans
(M)	AKIEIYTTAR (2)	gi|29542079|gb|aao91017.1| +13	3.7e4	0.72	6790-6871
(R)	CPYCVR (1)	gi|29542079|gb|aao91017.1| +14	8.6e3	0.81	4744
(R)	DEM*LSR (1)	gi|29542079|gb|aao91017.1| +13	2.3e4	0.71	3450
(R)	GIGGFDELWELEQSK (1)	gi|29542079|gb|aao91017.1| +13	4.5e3	0.90	12859
(K)	GLDYM*EIR (1)	gi|29542079|gb|aao91017.1| +13	1.2e4	0.88	7601
(K)	GLDYMEIR (1)	gi|29542079|gb|aao91017.1| +13	8.1e3	0.81	9558
(R)	IDEAPEKRDEM*LSR (1)	gi|29542079|gb|aao91017.1| +13	6.7e4	0.67	4402
(R)	IDEAPEKRDEMLSR (1)	gi|29542079|gb|aao91017.1| +13	7.6e3	0.11	5378
(K)	IEIYTTAR (1)	gi|29542079|gb|aao91017.1| +13	7.3e3	0.71	7031
(K)	KLDELLKT (2)	gi|29542079|gb|aao91017.1| +13	4.5e4	0.76	8055-8094
(R)	TVPQIFINGR (1)	gi|29542079|gb|aao91017.1| +13	1.9e4	0.55	9807

n gi|29541504|gb|AAO90444.1| **MS / MS Spectra:** 14 **Sum TIC:** 2.2e5 **Avg TIC:** 1.6e4 **Cov:** 30.8% **Uniq:** 6
enhanced entry protein [Coxiella burnetii RSA 493]

(P1)	Sequence (count)	Reference (+addnl)	Σ TIC	Sf_{max}	Scans
(A)	AKTGEQHFPSGCR (4)	gi|29541504|gb|aao90444.1| +13	1.1e5	0.96	5339-5694
(K)	FAEHNQGTYWILK (2)	gi|29541504|gb|aao90444.1| +13	2.2e4	0.94	11465-11489
(R)	GAIDNGILLR (3)	gi|29541504|gb|aao90444.1| +13	3.5e4	0.87	10400-10700
(A)	LNQPFLQLK (3)	gi|29541504|gb|aao90444.1| +13	3.5e4	0.75	9335-11194
(R)	MVGYEFK (1)	gi|29541504|gb|aao90444.1| +13	2.8e3	0.80	9427
(E)	QHFPSGCRMVGYEFK (1)	gi|29541504|gb|aao90444.1| +13	1.7e4	0.11	6803

o gi|29541010|gb|AAO89953.1| **MS / MS Spectra:** 7 **Sum TIC:** 2.2e5 **Avg TIC:** 3.1e4 **Cov:** 19.5% **Uniq:** 3
hypothetical protein CBU_0400 [Coxiella burnetii RSA 493]

(P1)	Sequence (count)	Reference (+addnl)	Σ TIC	Sf_{max}	Scans
(K)	AQPPTITTSDGK (2)	gi|29541010|gb|aao89953.1| +7	6.6e4	0.42	6259-6442
(R)	SRTPLQLINQTVEPHNM*M*K (2)	gi|29541010|gb|aao89953.1| +7	7.5e4	0.96	10748-10838
(R)	TPLQLINQTVEPHNM*M*K (2)	gi|29541010|gb|aao89953.1| +7	4.0e4	0.87	10763-10947
(R)	TPLQLINQTVEPHNMM*K (1)	gi|29541010|gb|aao89953.1| +7	3.9e4	0.57	11399

p gi|206584112|gb|AAO91177.2| **MS / MS Spectra:** 20 **Sum TIC:** 2.1e5 **Avg TIC:** 1.1e4 **Cov:** 44.9% **Uniq:** 12
hypothetical exported protein [Coxiella burnetii RSA 493]

(P1)	Sequence (count)	Reference (+addnl)	Σ TIC	Sf_{max}	Scans
(K)	AYQTM*SGGGSAK (1)	gi|206584112|gb|aao91177.2| +13	4.0e3	0.94	3023
(K)	AYQTMSGGGSAK (1)	gi|206584112|gb|aao91177.2| +13	1.5e4	0.96	3881
(Y)	IAGQPQPAGLR (2)	gi|206584112|gb|aao91177.2| +13	5.4e4	0.91	5369-5525
(Y)	IAGQPQPAGLRQLK (2)	gi|206584112|gb|aao91177.2| +13	3.6e4	0.89	6107-6126
(Y)	IAGQPQPAGLRQLKK (2)	gi|206584112|gb|aao91177.2| +13	2.3e4	0.79	5607-5633
(K)	IKAPVDL (2)	gi|206584112|gb|aao91177.2| +13	2.3e4	0.61	7865-8842
(K)	IKAPVDLV (2)	gi|206584112|gb|aao91177.2| +13	1.5e4	0.63	8756-8768
(K)	KLKIKAPVD (1)	gi|206584112|gb|aao91177.2| +13	5.5e3	0.23	5728
(S)	LIYIAGQPQPAGLR (3)	gi|206584112|gb|aao91177.2| +13	1.7e4	0.93	8287-9321
(K)	LKIKAPVD (1)	gi|206584112|gb|aao91177.2| +13	7.2e3	0.66	6158
(K)	LKIKAPVDL (1)	gi|206584112|gb|aao91177.2| +13	5.8e3	0.73	8517
(A)	TLDSLIYIAGQPQPAGLR (1)	gi|206584112|gb|aao91177.2| +13	2.5e3	0.38	10786
(I)	YIAGQPQPAGLR (1)	gi|206584112|gb|aao91177.2| +13	5.9e3	0.81	6008

q gi|29542637|gb|AAO91571.1| **MS / MS Spectra:** 9 **Sum TIC:** 2.1e5 **Avg TIC:** 2.3e4 **Cov:** 42.0% **Uniq:** 6
thioredoxin [Coxiella burnetii RSA 493]

(P1)	Sequence (count)	Reference (+addnl)	Σ TIC	Sf_{max}	Scans
(R)	EGEVVDR (2)	gi|29542637|gb|aao91571.1| +13	3.8e4	0.29	3523-4827
(R)	GIPSLLIFR (1)	gi|29542637|gb|aao91571.1| +13	4.6e3	0.50	13668
(K)	LNVDENAQTPTK (1)	gi|29542637|gb|aao91571.1| +9	5.4e4	0.36	6743
(K)	MISPVVEEIAK (1)	gi|29542637|gb|aao91571.1| +13	1.9e4	0.91	10906
(K)	MISPVVEEIAKEYAGR (2)	gi|29542637|gb|aao91571.1| +13	3.0e4	0.95	13197-13199
(K)	VFKLNVDENAQTPTK (2)	gi|29542637|gb|aao91571.1| +9	6.2e4	0.96	8978-8990

r gi|206583949|gb|AAO90419.2| **MS / MS Spectra:** 18 **Sum TIC:** 2.0e5 **Avg TIC:** 1.1e4 **Cov:** 44.7% **Uniq:** 12
thiol:disulfide interchange protein [Coxiella burnetii RSA 493]

(P1)	Sequence (count)	Reference (+addnl)	Σ TIC	Sf_{max}	Scans
(K)	ATTIDTK (4)	gi|206583949|gb|aao90419.2| +13	3.1e4	0.36	4145-4365
(A)	FEDLKCPNCAR (1)	gi|206583949|gb|aao90419.2| +13	4.6e3	0.86	5546
(R)	FNVEVLPAIK (3)	gi|206583949|gb|aao90419.2| +13	6.8e4	0.96	10048-11362
(R)	FNVEVLPAIKK (2)	gi|206583949|gb|aao90419.2| +13	8.6e3	0.86	8911-10117
(R)	FNVEVLPAIKKK (1)	gi|206583949|gb|aao90419.2| +13	6.3e3	0.81	8301

(K)	GQPTLGNPAAPVHIVAFEDLK (1)	gi|206583949|gb|aao90419.2| +13	2.9e4	0.43	11375
(A)	LSKPAALK (1)	gi|206583949|gb|aao90419.2| +13	6.8e3	0.27	5756
(R)	NSVPQANMK (1)	gi|206583949|gb|aao90419.2| +13	1.0e4	0.35	5433
(K)	QLSNCIFSSR (1)	gi|206583949|gb|aao90419.2| +13	4.6e3	0.10	8925
(A)	RNSVPQANMK (1)	gi|206583949|gb|aao90419.2| +13	3.1e3	0.77	4076
(K)	YINTGVAK (1)	gi|206583949|gb|aao90419.2| +13	8.5e3	0.33	4675
(R)	YSGALQK (1)	gi|206583949|gb|aao90419.2| +13	2.3e4	0.83	5120

s gi|29541070|gb|AAO90013.1| **MS/MS Spectra:** 32 **Sum TIC:** 2.0e5 **Avg TIC:** 6.3e3 **Cov:** 43.0% **Uniq:** 18
dihydrolipoamide dehydrogenase [Coxiella burnetii RSA 493]

(P1)	**Sequence (count)**	**Reference (+addnl)**	**Σ TIC**	**Sf_{max}**	**Scans**
(K)	FSSSNELAVENK (1)	gi|29541070|gb|aao90013.1| +11	1.1e4	0.96	6736
(K)	GVFPWAASGR (5)	gi|29541070|gb|aao90013.1| +11	2.4e4	0.76	10788-12206
(K)	ISVVEM*M*DQLIPGADADVVKPLHQR (1)	gi|29541070|gb|aao90013.1| +11	6.2e3	0.75	9506
(K)	KDGLYVTFEGENAPK (2)	gi|29541070|gb|aao90013.1| +11	8.9e3	0.94	8820-9526
(K)	KDGLYVTFEGENAPKEPK (4)	gi|29541070|gb|aao90013.1| +11	3.4e4	0.96	8289-9167
(R)	KVEIITGYGK (1)	gi|29541070|gb|aao90013.1| +11	7.2e3	0.95	8150
(R)	LAAEVIAGIK (1)	gi|29541070|gb|aao90013.1| +5	3.8e3	0.24	9057
(K)	LIDAEKAGVK (1)	gi|29541070|gb|aao90013.1| +11	6.0e3	0.66	6286
(K)	LLFDEK (2)	gi|29541070|gb|aao90013.1| +11	9.1e3	0.63	9056-9728
(K)	RYEEILLK (2)	gi|29541070|gb|aao90013.1| +11	1.4e4	0.83	8380-9178
(R)	SWKENVVK (1)	gi|29541070|gb|aao90013.1| +11	6.0e3	0.48	6247
(K)	TEVVVLGSGPGGYAAAFR (2)	gi|29541070|gb|aao90013.1| +7	1.3e4	0.98	10718-11441
(R)	TNVSHIYAIGDVVGQPM*LAHK (1)	gi|29541070|gb|aao90013.1| +11	2.9e3	0.95	9380
(K)	VDDKGYIAVDK (1)	gi|29541070|gb|aao90013.1| +11	1.1e4	0.33	6965
(K)	VDDKGYIAVDKQM*R (2)	gi|29541070|gb|aao90013.1| +11	8.4e3	0.86	5850-6533
(K)	VIDDAKDM*SSFGIDFGK (2)	gi|29541070|gb|aao90013.1| +11	2.0e4	0.87	9577-10361
(R)	YEEILLK (2)	gi|29541070|gb|aao90013.1| +11	1.2e4	0.89	8938-9652
(R)	YETIGGVCLNVGCIPSK (1)	gi|29541070|gb|aao90013.1| +11	5.4e3	0.87	10424

t gi|29542582|gb|AAO91516.1| **MS/MS Spectra:** 25 **Sum TIC:** 2.0e5 **Avg TIC:** 7.9e3 **Cov:** 42.9% **Uniq:** 20
hypothetical exported protein [Coxiella burnetii RSA 493]

(P1)	**Sequence (count)**	**Reference (+addnl)**	**Σ TIC**	**Sf_{max}**	**Scans**
(R)	DQYHEALQK (2)	gi|29542582|gb|aao91516.1| +7	2.9e4	0.89	4381-6017
(A)	ERLIPIYR (1)	gi|29542582|gb|aao91516.1| +13	5.1e4	0.86	7968
(V)	FIRAEHNEK (2)	gi|29542582|gb|aao91516.1| +13	1.2e4	0.51	3488-3511
(V)	FIRAEHNEKGKR (1)	gi|29542582|gb|aao91516.1| +13	2.8e3	0.60	3335
(V)	GWVNRDQYHEALQK (1)	gi|29542582|gb|aao91516.1| +7	6.0e3	0.89	6297
(R)	HVFWDM*DKLM*A (1)	gi|29542582|gb|aao91516.1| +13	4.8e3	0.45	8794
(K)	ILQKLSPAER (1)	gi|29542582|gb|aao91516.1| +13	6.8e3	0.69	5644
(I)	LQKLSPAER (1)	gi|29542582|gb|aao91516.1| +13	5.4e3	0.47	4735
(N)	LYEKPQSNAK (1)	gi|29542582|gb|aao91516.1| +13	4.6e3	0.59	3542
(Q)	LYEQIKNQQAK (2)	gi|29542582|gb|aao91516.1| +13	1.8e4	0.92	4857-4888
(Q)	LYEQIKNQQAKESR (1)	gi|29542582|gb|aao91516.1| +13	4.8e3	0.95	4898
(V)	NRDQYHEALQK (1)	gi|29542582|gb|aao91516.1| +7	3.4e3	0.37	4148
(E)	RLIPIYR (1)	gi|29542582|gb|aao91516.1| +13	8.8e3	0.45	7859
(T)	VFIRAEHNEK (1)	gi|29542582|gb|aao91516.1| +13	5.1e3	0.70	4614
(H)	VFWDMDKLM*AQQMR (1)	gi|29542582|gb|aao91516.1| +13	6.5e3	0.18	10662
(L)	YEKPQSNAK (1)	gi|29542582|gb|aao91516.1| +13	4.5e3	0.64	2944
(L)	YEQIKNQQAK (2)	gi|29542582|gb|aao91516.1| +13	9.6e3	0.92	3381-3500
(L)	YEQIKNQQAKESR (1)	gi|29542582|gb|aao91516.1| +13	3.3e3	0.88	3510
(K)	YYQPDIQT (1)	gi|29542582|gb|aao91516.1| +13	4.5e3	0.11	7647
(K)	YYQPDIQTV (2)	gi|29542582|gb|aao91516.1| +13	7.0e3	0.52	7932-7938

u gi|29541811|gb|AAO90750.1| **MS/MS Spectra:** 25 **Sum TIC:** 1.9e5 **Avg TIC:** 7.6e3 **Cov:** 27.4% **Uniq:** 9
malate dehydrogenase [Coxiella burnetii RSA 493]

(P1)	**Sequence (count)**	**Reference (+addnl)**	**Σ TIC**	**Sf_{max}**	**Scans**
(K)	AGVDVPSVK (5)	gi|29541811|gb|aao90750.1| +18	4.1e4	0.88	6722-7445
(R)	AIGQLALK (1)	gi|29541811|gb|aao90750.1| +18	7.5e3	0.39	8956
(K)	AINENAASDVR (3)	gi|29541811|gb|aao90750.1| +18	2.5e4	0.97	5915-6124
(K)	DLLEKNGSIFAGQGK (1)	gi|29541811|gb|aao90750.1| +18	4.5e3	0.52	9865
(D)	ELREER (1)	gi|29541811|gb|aao90750.1| +18	3.2e3	0.50	3807
(R)	FYAM*TR (3)	gi|29541811|gb|aao90750.1| +18	4.3e4	0.83	5372-5947
(R)	IFVVGNPCNTNCLIAM*NNAPDIPKDR (1)	gi|29541811|gb|aao90750.1| +18	1.1e4	0.80	10465
(K)	NGSIFAGQGK (3)	gi|29541811|gb|aao90750.1| +18	2.3e4	0.94	6796-7493
(R)	NM*VVTSDPR (4)	gi|29541811|gb|aao90750.1| +18	1.7e4	0.90	4716-5205
(R)	NMVVTSDPR (3)	gi|29541811|gb|aao90750.1| +18	1.6e4	0.82	5872-6515

v gi|212010281|gb|ACJ17662.1| **MS/MS Spectra:** 12 **Sum TIC:** 1.9e5 **Avg TIC:** 1.6e4 **Cov:** 76.9% **Uniq:** 8
hypothetical exported protein [Coxiella burnetii CbuG_Q212]

(P1)	**Sequence (count)**	**Reference (+addnl)**	**Σ TIC**	**Sf_{max}**	**Scans**
(R)	EICYM*VNETK (2)	gi|212010281|gb|acj17662.1| +5	2.7e4	0.91	6114-6150
(R)	EICYMVNETK (1)	gi|212010281|gb|acj17662.1| +5	2.2e4	0.93	7545
(K)	GM*VLFYLKPFLDK (1)	gi|212010281|gb|acj17662.1| +13	3.2e3	0.53	13270
(-)	M*LKKGRMMK (1)	gi|212010281|gb|acj17662.1| +1	7.5e3	0.52	6840
(R)	MEAEGLANSLTEKETR (1)	gi|212010281|gb|acj17662.1| +13	2.5e4	0.96	8250
(T)	RLYNDLLSYQCIAQQAPDKPLAAR (1)	gi|212010281|gb|acj17662.1| +11	1.7e4	0.94	10433
(N)	SLTEKETR (1)	gi|212010281|gb|acj17662.1| +13	6.3e3	0.82	3140

(K)	TNPVFSLR (3)	gi|212010281|gb|acj17662.1| +13	6.8e4	0.76	9391-9510
(R)	YLSVNEAYIHSICNATTNADYCK (1)	gi|212010281|gb|acj17662.1| +13	1.4e4	0.96	10238

w gi|29542273|gb|AAO91210.1| **MS/MS Spectra:** 8 **Sum TIC:** 1.6e5 **Avg TIC:** 2.0e4 **Cov:** 33.8% **Uniq:** 5
glycine cleavage system H protein [Coxiella burnetii RSA 493]

(P1)	**Sequence (count)**	**Reference (+addnl)**	**Σ TIC**	**Sf_{max}**	**Scans**
(M)	AEFPAELYYSK (2)	gi|29542273|gb|aao91210.1| +18	4.3e4	0.95	11125-11337
(R)	DPYGDGWLYR (1)	gi|29542273|gb|aao91210.1| +18	1.1e4	0.96	11465
(K)	NHEWMR (2)	gi|29542273|gb|aao91210.1| +18	5.2e4	0.84	7118-7263
(K)	VIEINNALENEPATVNR (2)	gi|29542273|gb|aao91210.1| +18	1.4e4	0.98	9883-9908
(K)	VIEINNALENEPATVNRD (1)	gi|29542273|gb|aao91210.1| +18	4.0e4	0.97	10165

x gi|29542023|gb|AAO90961.1| **MS/MS Spectra:** 9 **Sum TIC:** 1.6e5 **Avg TIC:** 1.7e4 **Cov:** 43.6% **Uniq:** 6
DNA-binding protein HU [Coxiella burnetii RSA 493]

(P1)	**Sequence (count)**	**Reference (+addnl)**	**Σ TIC**	**Sf_{max}**	**Scans**
(V)	GFGSFTTSQR (2)	gi|29542023|gb|aao90961.1| +11	3.1e4	0.89	7910-7933
(K)	KLKDAVNKEK (1)	gi|29542023|gb|aao90961.1| +11	1.3e4	0.29	2863
(K)	LKDAVNK (1)	gi|29542023|gb|aao90961.1| +11	3.6e4	0.67	2987
(K)	LKDAVNKEKV (1)	gi|29542023|gb|aao90961.1| +11	4.1e4	0.93	3565
(R)	NPQTGDTM*QIPAATVAR (1)	gi|29542023|gb|aao90961.1| +11	9.0e3	0.95	6210
(R)	TGRNPQTGDTM*QIPAATVAR (2)	gi|29542023|gb|aao90961.1| +11	2.3e4	0.94	5630-5647
(R)	TGRNPQTGDTMQIPAATVAR (1)	gi|29542023|gb|aao90961.1| +11	3.3e3	0.54	7487

y gi|29541088|gb|AAO90031.1| **MS/MS Spectra:** 13 **Sum TIC:** 1.5e5 **Avg TIC:** 1.2e4 **Cov:** 20.4% **Uniq:** 7
arginine-binding protein [Coxiella burnetii RSA 493]

(P1)	**Sequence (count)**	**Reference (+addnl)**	**Σ TIC**	**Sf_{max}**	**Scans**
(G)	EYVLIGK (1)	gi|29541088|gb|aao90031.1| +13	5.4e3	0.11	8669
(G)	EYVLIGKPVNDPNYFGK (1)	gi|29541088|gb|aao90031.1| +13	3.7e4	0.96	10349
(K)	FDALFGGM*NITTAR (1)	gi|29541088|gb|aao90031.1| +13	7.2e3	0.97	10806
(K)	LGKFDALFGGM*NITTAR (3)	gi|29541088|gb|aao90031.1| +13	5.4e4	0.98	11087-11176
(K)	LGKFDALFGGMNITTAR (1)	gi|29541088|gb|aao90031.1| +13	2.4e3	0.97	12451
(K)	NTPLTLSK (2)	gi|29541088|gb|aao90031.1| +11	10.0e3	0.30	7228-7267
(R)	REYVLIGKPVNDPNYFGK (1)	gi|29541088|gb|aao90031.1| +5	6.4e3	0.85	9719
(R)	VDAVVGDTPLIK (3)	gi|29541088|gb|aao90031.1| +11	2.9e4	0.96	9187-9336

z uc|1363944|pir||JC4313 **MS/MS Spectra:** 12 **Sum TIC:** 1.5e5 **Avg TIC:** 1.3e4 **Cov:** 18.8% **Uniq:** 7
CON keratin 16, type I - human gi|1195531|bbs|172338 (S79867) type I keratin 16, K16 [human, epidermal keratinocytes, Peptide, 473 aa] [Homo sapiens]

(P1)	**Sequence (count)**	**Reference (+addnl)**	**Σ TIC**	**Sf_{max}**	**Scans**
(R)	DAETWFLSK (2)	uc|1363944|pir||jc4313 +3	8.6e3	0.93	10956-11677
(R)	EVFTSSSSSSSR (1)	uc|1363944|pir||jc4313 +1	7.4e3	0.73	5775
(R)	GQTGGDVNVEM*DAAPGVDLSR (3)	uc|1363944|pir||jc4313 +3	3.0e4	0.96	7860-8639
(K)	GSCGIGGGIGGGSSR (3)	uc|1363944|pir||jc4313	8.1e4	0.94	6679-7004
(R)	LLEGEDAHLSSQQASGQSYSSR (1)	uc|1363944|pir||jc4313 +3	1.2e4	0.93	7523
(R)	TKYEHELALR (1)	uc|1363944|pir||jc4313	3.3e3	0.86	6643
(V)	VFTSSSSSSSR (1)	uc|1363944|pir||jc4313 +3	8.5e3	0.13	6530

Aa gi|206584008|gb|AAO90692.2| **MS/MS Spectra:** 5 **Sum TIC:** 1.5e5 **Avg TIC:** 3.0e4 **Cov:** 33.3% **Uniq:** 4
glycine-rich RNA-binding protein [Coxiella burnetii RSA 493]

(P1)	**Sequence (count)**	**Reference (+addnl)**	**Σ TIC**	**Sf_{max}**	**Scans**
(K)	ANGIDLQGR (1)	gi|206584008|gb|aao90692.2| +13	6.7e4	0.81	5922
(K)	GFAFITYGTQDAAQEAVSK (2)	gi|206584008|gb|aao90692.2| +13	4.0e4	0.99	11054-11101
(K)	LIM*DRETGR (1)	gi|206584008|gb|aao90692.2| +13	1.3e4	0.23	3604
(R)	SKGFAFITYGTQDAAQEAVSK (1)	gi|206584008|gb|aao90692.2| +13	2.9e4	0.97	10201

Ab gi|29541847|gb|AAO90785.1| **MS/MS Spectra:** 14 **Sum TIC:** 1.4e5 **Avg TIC:** 1.0e4 **Cov:** 40.0% **Uniq:** 10
thiol-disulfide isomerase and thioredoxin [Coxiella burnetii RSA 493]

(P1)	**Sequence (count)**	**Reference (+addnl)**	**Σ TIC**	**Sf_{max}**	**Scans**
(K)	APDFNLPDAVSGK (1)	gi|29541847|gb|aao90785.1| +13	8.0e3	0.95	10113
(K)	APDFNLPDAVSGKK (3)	gi|29541847|gb|aao90785.1| +13	3.1e4	0.94	9510-10079
(K)	AYQAECTPDFYVFDK (1)	gi|29541847|gb|aao90785.1| +13	6.0e3	0.97	11381
(R)	FDSATPGR (1)	gi|29541847|gb|aao90785.1| +13	3.8e3	0.27	5235
(R)	GRFDSATPGR (1)	gi|29541847|gb|aao90785.1| +13	4.0e3	0.29	5547
(R)	GRFDSATPGRDTPVTGEDLR (2)	gi|29541847|gb|aao90785.1| +13	4.0e4	0.55	7482-7511
(K)	KM*SLAQLK (1)	gi|29541847|gb|aao90785.1| +13	7.7e3	0.66	6147
(K)	LVEVAKEYQAK (1)	gi|29541847|gb|aao90785.1| +11	5.0e3	0.86	6834
(K)	MSLAQLK (1)	gi|29541847|gb|aao90785.1| +13	7.2e3	0.71	8791
(K)	NLACVYR (2)	gi|29541847|gb|aao90785.1| +13	3.0e4	0.82	7343-7345

Ac gi|206583994|gb|AAO90616.2| **MS/MS Spectra:** 20 **Sum TIC:** 1.4e5 **Avg TIC:** 6.9e3 **Cov:** 26.3% **Uniq:** 14
membrane-bound lytic murein transglycosylase D precursor [Coxiella burnetii RSA 493]

(P1)	**Sequence (count)**	**Reference (+addnl)**	**Σ TIC**	**Sf_{max}**	**Scans**
(R)	AAQQYNQR (1)	gi|206583994|gb|aao90616.2| +5	6.3e3	0.45	3133
[illegible]	[illegible]	[illegible]	[illegible]	[illegible]	[illegible]

(A)	IASQKPAAR (1)	gi|206583994|gb|aao90616.2| +5	7.0e3	0.82	2934
(R)	IYHTNIA (1)	gi|206583994|gb|aao90616.2| +5	5.3e3	0.16	5681
(R)	LAGVSSSVIR (1)	gi|206583994|gb|aao90616.2| +5	1.4e4	0.93	6557
(M)	NSQIDRAEAAR (2)	gi|206583994|gb|aao90616.2| +5	1.3e4	0.55	4138-4178
(R)	SGETLQSIAR (2)	gi|206583994|gb|aao90616.2| +5	1.5e4	0.94	6140-6711
(A)	TAAYDAGM*GAVR (1)	gi|206583994|gb|aao90616.2| +5	6.3e3	0.97	4725
(A)	TAAYDAGMGAVR (1)	gi|206583994|gb|aao90616.2| +5	4.5e3	0.93	6160
(K)	TAIASQKPAAR (1)	gi|206583994|gb|aao90616.2| +5	5.0e3	0.70	3477
(R)	TANLWPIM*TAR (1)	gi|206583994|gb|aao90616.2| +1	7.2e3	0.10	9401
(R)	TYSAPVGEQPATVA (1)	gi|206583994|gb|aao90616.2| +5	7.9e3	0.44	7062
(A)	VNM*NSQIDR (1)	gi|206583994|gb|aao90616.2| +5	4.5e3	0.81	3590
(A)	VNM*NSQIDRAEAAR (2)	gi|206583994|gb|aao90616.2| +5	2.4e4	0.35	4560-4575
(A)	VNMNSQIDRAEAAR (2)	gi|206583994|gb|aao90616.2| +5	1.0e4	0.77	5851-5885
(A)	YDAGM*GAVR (1)	gi|206583994|gb|aao90616.2| +5	5.7e3	0.62	3986

Ad uc|1346349|sp|P48669|K2CF_HUMAN **MS/MS Spectra:** 15 **Sum TIC:** 1.3e5 **Avg TIC:** 8.9e3 **Cov:** 20.7% **Uniq:** 11

CON Keratin, type ii cytoskeletal 6f (cytokeratin 6F) (CK 6F) (K6F keratin) gi|2119219|pir||I61771 keratin type II - human gi|908805 (L42612) keratin type II [Homo sapiens]

(P1)	Sequence (count)	Reference (+addnl)	Σ TIC	Sf_{max}	Scans
(K)	ADTLTDEINFLR (2)	uc|1346349|sp|p48669|k2cf_hum +8	1.1e4	0.95	11794-12523
(R)	ATGGGLSSVGGGSSTIK (1)	uc|1346349|sp|p48669|k2cf_hum +3	1.6e4	0.98	7282
(R)	ATGGGLSSVGGGSSTIKYTTTSSSSR (1)	uc|1346349|sp|p48669|k2cf_hum +3	9.0e3	0.36	7803
(K)	DVDAAYM*NKVELQAK (2)	uc|1346349|sp|p48669|k2cf_hum +8	1.6e4	0.50	7757-7797
(R)	GFSANSAR (1)	uc|1346349|sp|p48669|k2cf_hum +3	1.5e4	0.22	5414
(R)	ISIGGGSCAISGGYGSR (2)	uc|1346349|sp|p48669|k2cf_hum +6	1.7e4	0.96	8790-8845
(R)	NTKQEIAEINR (2)	uc|1346349|sp|p48669|k2cf_hum +8	1.8e4	0.89	6515-6521
(R)	SLYGLGGSKR (1)	uc|1346349|sp|p48669|k2cf_hum +6	8.9e3	0.38	8025
(R)	SRAEAESWYQTK (1)	uc|1346349|sp|p48669|k2cf_hum +8	2.7e3	0.27	7000
(R)	VRAEER (1)	uc|1346349|sp|p48669|k2cf_hum +4	1.2e4	0.35	3397
(K)	YTTTSSSSR (1)	uc|1346349|sp|p48669|k2cf_hum +8	7.3e3	0.74	3984

Ae gi|29542210|gb|AAO91147.1| **MS/MS Spectra:** 10 **Sum TIC:** 1.3e5 **Avg TIC:** 1.3e4 **Cov:** 50.0% **Uniq:** 6

hypothetical membrane associated protein [Coxiella burnetii RSA 493]

(P1)	Sequence (count)	Reference (+addnl)	Σ TIC	Sf_{max}	Scans
(K)	GDSVSSAVPHGGGFSAAPK (1)	gi|29542210|gb|aao91147.1| +13	1.8e4	0.92	8085
(K)	KQDSSNIHWF (4)	gi|29542210|gb|aao91147.1| +13	5.0e4	0.86	10574-11588
(K)	LPTTALPIVNPPANTPSPVADQPSSTQTKK (1)	gi|29542210|gb|aao91147.1| +13	3.8e4	0.83	10737
(K)	QDSSNIHWF (2)	gi|29542210|gb|aao91147.1| +13	1.8e4	0.85	12018-12290
(A)	SACSSISVDHCSALLSTR (1)	gi|29542210|gb|aao91147.1| +13	5.1e3	0.90	7320
(P)	VADQPSSTQTK (1)	gi|29542210|gb|aao91147.1| +13	4.3e3	0.28	4124

Af gi|29540885|gb|AAO89829.1| **MS/MS Spectra:** 8 **Sum TIC:** 1.3e5 **Avg TIC:** 1.6e4 **Cov:** 46.8% **Uniq:** 8

single-strand DNA binding protein [Coxiella burnetii RSA 493]

(P1)	Sequence (count)	Reference (+addnl)	Σ TIC	Sf_{max}	Scans
(R)	DKQTGELQER (1)	gi|29540885|gb|aao89829.1| +12	4.0e4	0.93	4842
(R)	GGGNSGNYGNHSEGGASNK (1)	gi|29540885|gb|aao89829.1| +8	1.3e4	0.68	4149
(R)	GVNKVILIGNLGQDPEVR (1)	gi|29540885|gb|aao89829.1| +12	1.5e4	0.98	11030
(R)	LAEIVGEYLR (1)	gi|29540885|gb|aao89829.1| +12	6.5e3	0.94	11502
(R)	LAEIVGEYLRK (1)	gi|29540885|gb|aao89829.1| +12	1.2e4	0.22	10632
(K)	QTGELQER (1)	gi|29540885|gb|aao89829.1| +12	1.9e4	0.57	4932
(K)	VILIGNLGQDPEVR (1)	gi|29540885|gb|aao89829.1| +12	2.1e4	0.97	11510
(R)	YTTEIIANEMHM*LDNR (1)	gi|29540885|gb|aao89829.1| +12	6.4e3	0.11	11560

Ag gi|29540988|gb|AAO89931.1| **MS/MS Spectra:** 15 **Sum TIC:** 1.3e5 **Avg TIC:** 8.7e3 **Cov:** 35.7% **Uniq:** 10

hypothetical membrane associated protein [Coxiella burnetii RSA 493]

(P1)	Sequence (count)	Reference (+addnl)	Σ TIC	Sf_{max}	Scans
(A)	AFDYPDGIILPM*QK (1)	gi|29540988|gb|aao89931.1| +5	1.3e4	0.95	9862
(F)	DYPDGIILPM*QK (3)	gi|29540988|gb|aao89931.1| +5	4.6e4	0.94	8026-8444
(F)	DYPDGIILPMQK (2)	gi|29540988|gb|aao89931.1| +5	1.8e4	0.83	9760-9813
(A)	FDYPDGIILPM*QK (1)	gi|29540988|gb|aao89931.1| +5	6.2e3	0.92	9702
(A)	FDYPDGIILPMQK (1)	gi|29540988|gb|aao89931.1| +5	3.8e3	0.91	10820
(K)	GYLPANR (1)	gi|29540988|gb|aao89931.1| +13	8.1e3	0.23	5522
(K)	KGYLPANR (1)	gi|29540988|gb|aao89931.1| +13	6.6e3	0.65	5090
(K)	KGYLPANRLVHSK (1)	gi|29540988|gb|aao89931.1| +13	9.0e3	0.12	6033
(H)	KKGYLPANR (1)	gi|29540988|gb|aao89931.1| +13	5.0e3	0.10	7299
(K)	STPGYVTVHR (1)	gi|29540988|gb|aao89931.1| +13	6.2e3	0.87	5258
(K)	TKSM*AFGNLV (1)	gi|29540988|gb|aao89931.1| +13	7.2e3	0.15	8304
(K)	TKSM*AFGNLVFG (1)	gi|29540988|gb|aao89931.1| +13	3.1e3	0.26	10490

Ah uc|Q86YZ3|HORN_HUMAN **MS/MS Spectra:** 19 **Sum TIC:** 1.3e5 **Avg TIC:** 6.6e3 **Cov:** 14.1% **Uniq:** 26

CON Hornerin - Homo sapiens

(P1)	Sequence (count)	Reference (+addnl)	Σ TIC	Sf_{max}	Scans
(R)	GEQHGSSSGSSSSYGQHGSGSR (2)	uc|q86yz3|horn_human	1.1e4	0.96	4022-4049
(R)	GPYESGSGHSSGLGHR (2)	uc|q86yz3|horn_human	1.0e4	0.97	3543-5308
(R)	GSGSGQSPSSGQHGTGFGR (1)	uc|q86yz3|horn_human	5.7e3	0.94	5443
(R)	HGAGSGQSLSHGR (1)	uc|q86yz3|horn_human	8.4e3	0.82	4045

(R)	HGSGLGHSSSHGQHGSGSGR (1)	uc|q86yz3|horn_human	7.5e3	0.95	3335
(R)	HGSGSGQSPGHGQR (1)	uc|q86yz3|horn_human	5.1e3	0.87	3367
(R)	HGSGSGQSSGFGHK (1)	uc|q86yz3|horn_human	7.3e3	0.84	4199
(R)	HGSGSGQSSSYGPYGSGSGWSSSR (1)	uc|q86yz3|horn_human	6.5e3	0.84	7861
(R)	HGSSSGSSSHYGQHGSGSR (3)	uc|q86yz3|horn_human	1.9e4	0.96	3480-3498
(R)	SSSGSSSSYGQHGSGSR (1)	uc|q86yz3|horn_human	5.5e3	0.96	4029
(R)	SSSRGPYESR (3)	uc|q86yz3|horn_human	2.2e4	0.89	4244-4729
(R)	YGQQGSGSGQSPSR (2)	uc|q86yz3|horn_human	1.7e4	0.88	4413-4426

Ai gi|29541626|gb|AAO90565.1| **MS/MS Spectra:** 10 **Sum TIC:** 1.2e5 **Avg TIC:** 1.2e4 **Cov:** 47.1% **Uniq:** 3
carbon storage regulator [Coxiella burnetii RSA 493]

(P1)	Sequence (count)	Reference (+addnl)	Σ TIC	Sf_{max}	Scans
(K)	DISVHREEIYER (2)	gi|29541626|gb|aao90565.1| +16	2.4e4	0.64	7416-7423
(K)	LAQSEDQGEKTDEFE (7)	gi|29541626|gb|aao90565.1| +8	9.1e4	0.98	5492-7424
(-)	M*LILTR (1)	gi|29541626|gb|aao90565.1| +14	8.6e3	0.85	7206

Aj gi|206583771|gb|AAO89658.2| **MS/MS Spectra:** 14 **Sum TIC:** 1.2e5 **Avg TIC:** 8.6e3 **Cov:** 29.8% **Uniq:** 8
tol system periplasmic component [Coxiella burnetii RSA 493]

(P1)	Sequence (count)	Reference (+addnl)	Σ TIC	Sf_{max}	Scans
(A)	DAPVEDISAQPQPTK (1)	gi|206583771|gb|aao89658.2| +8	5.6e3	0.91	7471
(R)	GQLQVQER (1)	gi|206583771|gb|aao89658.2| +16	1.4e4	0.90	5550
(K)	LAIIDAEDGKIK (1)	gi|206583771|gb|aao89658.2| +16	5.4e3	0.85	9490
(R)	LQQLEEVDSATTTP (4)	gi|206583771|gb|aao89658.2| +16	3.0e4	0.96	8514-9138
(K)	NAAHEFQTVR (2)	gi|206583771|gb|aao89658.2| +16	2.1e4	0.92	6110-6529
(R)	NLELLNNQQR (2)	gi|206583771|gb|aao89658.2| +16	1.2e4	0.94	8585-8668
(R)	QALDLLTK (1)	gi|206583771|gb|aao89658.2| +16	6.8e3	0.17	10136
(K)	QHPESTAAQLANIR (2)	gi|206583771|gb|aao89658.2| +16	2.6e4	0.84	8311-8341

Ak gi|29541757|gb|AAO90696.1| **MS/MS Spectra:** 14 **Sum TIC:** 1.1e5 **Avg TIC:** 7.6e3 **Cov:** 32.1% **Uniq:** 11
hypothetical exported protein [Coxiella burnetii RSA 493]

(P1)	Sequence (count)	Reference (+addnl)	Σ TIC	Sf_{max}	Scans
(I)	DFKQPSNKK (1)	gi|29541757|gb|aao90696.1| +13	5.4e3	0.42	3360
(K)	GLSPQALQVGLK (1)	gi|29541757|gb|aao90696.1| +13	4.6e3	0.32	8508
(T)	LIDFKQPSNKK (1)	gi|29541757|gb|aao90696.1| +13	1.5e4	0.85	6192
(T)	LIDFKQPSNKKR (1)	gi|29541757|gb|aao90696.1| +13	3.9e3	0.14	5937
(R)	SWGCFAIDPK (1)	gi|29541757|gb|aao90696.1| +13	3.9e3	0.51	9612
(V)	TGESYYGHHGLSVR (1)	gi|29541757|gb|aao90696.1| +13	2.9e3	0.22	5908
(R)	THGAVKKPIM*T (2)	gi|29541757|gb|aao90696.1| +13	3.7e4	0.78	3521-3536
(R)	THGAVKKPIMT (1)	gi|29541757|gb|aao90696.1| +13	4.7e3	0.58	4235
(R)	THGAVKKPIMTLI (1)	gi|29541757|gb|aao90696.1| +13	5.9e3	0.47	8896
(A)	TKSFAEK (1)	gi|29541757|gb|aao90696.1| +13	6.4e3	0.64	3392
(A)	TKSFAEKVGR (1)	gi|29541757|gb|aao90696.1| +13	4.0e3	0.91	4627
(S)	YYGHHGLSVR (2)	gi|29541757|gb|aao90696.1| +13	1.2e4	0.87	5362-5388

Al gi|161762893|gb|ABX78535.1| **MS/MS Spectra:** 7 **Sum TIC:** 1.1e5 **Avg TIC:** 1.5e4 **Cov:** 13.8% **Uniq:** 6
glycosyl hydrolase, family 18 protein [Coxiella burnetii RSA 331]

(P1)	Sequence (count)	Reference (+addnl)	Σ TIC	Sf_{max}	Scans
(R)	AVITQSSAR (2)	gi|161762893|gb|abx78535.1| +1	5.4e4	0.94	3928-3933
(S)	LGHWNPDKMR (1)	gi|161762893|gb|abx78535.1| +1	4.9e3	0.74	6244
(K)	QNLIVIR (1)	gi|161762893|gb|abx78535.1| +3	9.3e3	0.19	7918
(K)	QNLIVIRR (1)	gi|161762893|gb|abx78535.1| +3	2.8e4	0.50	6946
(I)	TKQNLIVIR (1)	gi|161762893|gb|abx78535.1| +3	5.6e3	0.57	6966
(N)	YFSPQTDEVKK (1)	gi|161762893|gb|abx78535.1| +3	3.8e3	0.29	5596

Am gi|206584122|gb|AAO91218.2| **MS/MS Spectra:** 3 **Sum TIC:** 1.0e5 **Avg TIC:** 3.4e4 **Cov:** 5.2% **Uniq:** 3
thiol:disulfide interchange protein [Coxiella burnetii RSA 493]

(P1)	Sequence (count)	Reference (+addnl)	Σ TIC	Sf_{max}	Scans
(K)	SADYLGEPLWPR (1)	gi|206584122|gb|aao91218.2| +11	5.2e3	0.93	12309
(R)	VDTVKDYPGIGK (1)	gi|206584122|gb|aao91218.2| +13	3.7e4	0.96	7794
(R)	VDTVKDYPGIGKLPVYQGQLR (1)	gi|206584122|gb|aao91218.2| +13	6.0e4	0.95	10961

An gi|29541712|gb|AAO90651.1| **MS/MS Spectra:** 14 **Sum TIC:** 9.0e4 **Avg TIC:** 6.4e3 **Cov:** 30.6% **Uniq:** 13
enhanced entry protein [Coxiella burnetii RSA 493]

(P1)	Sequence (count)	Reference (+addnl)	Σ TIC	Sf_{max}	Scans
(I)	FYVFHK (1)	gi|29541712|gb|aao90651.1| +13	10.0e3	0.62	7746
(G)	IFYVFHK (1)	gi|29541712|gb|aao90651.1| +13	6.4e3	0.14	8983
(T)	IYSISPFPLR (1)	gi|29541712|gb|aao90651.1| +13	4.5e3	0.77	10764
(I)	KWGPISSGK (1)	gi|29541712|gb|aao90651.1| +13	5.0e3	0.27	6252
(A)	LHGSNEVTGYR (1)	gi|29541712|gb|aao90651.1| +13	4.6e3	0.42	6080
(K)	SGFECIK (1)	gi|29541712|gb|aao90651.1| +13	6.8e3	0.88	6748
(K)	SGFECIKVQR (1)	gi|29541712|gb|aao90651.1| +13	5.5e3	0.89	6622
(K)	SNVFPVE (1)	gi|29541712|gb|aao90651.1| +13	5.1e3	0.47	8063
(K)	SNVFPVET (1)	gi|29541712|gb|aao90651.1| +13	7.4e3	0.40	8108
(K)	SNVFPVETSGG (1)	gi|29541712|gb|aao90651.1| +13	2.6e3	0.53	7564
(A)	TIYSISPFPLR (1)	gi|29541712|gb|aao90651.1| +13	4.8e3	0.90	11001

(A)	YDPEGQLIK (1)	gi|29541712|gb|aao90651.1| +13	5.8e3	0.10	7702
(E)	YYGTGLCGK (2)	gi|29541712|gb|aao90651.1| +13	2.1e4	0.80	5855-6166

Ao uc|1346344|sp|P02538|K2CA_HUMAN **MS / MS Spectra:** 3 **Sum TIC:** 8.8e4 **Avg TIC:** 2.9e4 **Cov:** 6.6% **Uniq:** 3
CON Keratin, type ii cytoskeletal 6a (cytokeratin 6a) (CK 6a) (k6a keratin) gi|2119221|pir||A57398 keratin type II - human gi|908779 (L42583) keratin type II [Homo sapiens]

(P1)	**Sequence (count)**	**Reference (+addnl)**	**Σ TIC**	**Sf_{max}**	**Scans**
(R)	AIGGGLSSVGGGSSTIK (1)	uc|1346344|sp|p02538|k2ca_hum +4	9.4e3	0.94	8395
(R)	SGFSSVSVSR (1)	uc|1346344|sp|p02538|k2ca_hum	5.6e4	0.96	7592
(K)	YEELQVTAGR (1)	uc|1346344|sp|p02538|k2ca_hum +7	2.3e4	0.98	7858

Ap gi|206583770|gb|AAO89657.2| **MS / MS Spectra:** 6 **Sum TIC:** 8.4e4 **Avg TIC:** 1.4e4 **Cov:** 30.3% **Uniq:** 6
peptidoglycan-associated lipoprotein [Coxiella burnetii RSA 493]

(P1)	**Sequence (count)**	**Reference (+addnl)**	**Σ TIC**	**Sf_{max}**	**Scans**
(N)	AATGLSDGTGAQAYALAEGKGYQGQLK (1)	gi|206583770|gb|aao89657.2| +13	1.3e4	0.19	9065
(A)	ATGLSDGTGAQAYALAEGK (1)	gi|206583770|gb|aao89657.2| +13	4.6e3	0.95	9281
(R)	EYNIGLGWR (1)	gi|206583770|gb|aao89657.2| +13	3.2e3	0.24	10725
(R)	ILEQEGVAPK (1)	gi|206583770|gb|aao89657.2| +13	4.3e4	0.89	7094
(A)	NYLATHSTAK (1)	gi|206583770|gb|aao89657.2| +13	1.1e4	0.80	4026
(R)	VQANYLATHSTAK (1)	gi|206583770|gb|aao89657.2| +13	9.3e3	0.16	6892

Aq gi|206584067|gb|ACI15297.1| **MS / MS Spectra:** 4 **Sum TIC:** 7.9e4 **Avg TIC:** 2.0e4 **Cov:** 36.8% **Uniq:** 4
hypothetical protein CBU_1429a [Coxiella burnetii RSA 493]

(P1)	**Sequence (count)**	**Reference (+addnl)**	**Σ TIC**	**Sf_{max}**	**Scans**
(K)	LPASVLCCGSR (1)	gi|206584067|gb|aci15297.1| +5	4.1e4	0.60	7567
(K)	SYYNSK (1)	gi|206584067|gb|aci15297.1| +7	6.2e3	0.43	3695
(K)	TEFVCPANYTIPK (1)	gi|206584067|gb|aci15297.1| +7	2.6e4	0.97	9481
(K)	WSDCVLVPLGTK (1)	gi|206584067|gb|aci15297.1| +5	6.4e3	0.86	10828

Ar gi|29542203|gb|AAO91140.1| **MS / MS Spectra:** 6 **Sum TIC:** 7.7e4 **Avg TIC:** 1.3e4 **Cov:** 11.3% **Uniq:** 6
DotC [Coxiella burnetii RSA 493]

(P1)	**Sequence (count)**	**Reference (+addnl)**	**Σ TIC**	**Sf_{max}**	**Scans**
(R)	DYLGM*VLYR (1)	gi|29542203|gb|aao91140.1| +13	6.8e3	0.95	9402
(R)	DYLGMVLYRK (1)	gi|29542203|gb|aao91140.1| +13	3.8e3	0.13	9888
(R)	INDEIM*R (1)	gi|29542203|gb|aao91140.1| +13	2.9e4	0.89	4622
(A)	KVDLGVTGDANQIR (1)	gi|29542203|gb|aao91140.1| +3	9.0e3	0.54	7146
(K)	VDLGVTGDANQIR (1)	gi|29542203|gb|aao91140.1| +3	1.3e4	0.90	7719
(G)	VTGDANQIR (1)	gi|29542203|gb|aao91140.1| +13	1.6e4	0.72	5319

As gi|206583916|gb|AAO90271.2| **MS / MS Spectra:** 3 **Sum TIC:** 7.6e4 **Avg TIC:** 2.5e4 **Cov:** 7.7% **Uniq:** 2
hypothetical exported protein [Coxiella burnetii RSA 493]

(P1)	**Sequence (count)**	**Reference (+addnl)**	**Σ TIC**	**Sf_{max}**	**Scans**
(K)	DFGGAVYSPAK (2)	gi|206583916|gb|aao90271.2| +13	6.1e4	0.94	7373-8661
(K)	DFGGAVYSPAKK (1)	gi|206583916|gb|aao90271.2| +13	1.5e4	0.89	8001

At gi|206583959|gb|AAO90485.2| **MS / MS Spectra:** 9 **Sum TIC:** 7.5e4 **Avg TIC:** 8.3e3 **Cov:** 32.2% **Uniq:** 9
thioredoxin peroxidase [Coxiella burnetii RSA 493]

(P1)	**Sequence (count)**	**Reference (+addnl)**	**Σ TIC**	**Sf_{max}**	**Scans**
(K)	AGVVVLGISK (1)	gi|206583959|gb|aao90485.2| +6	1.3e4	0.96	10029
(K)	AGVVVLGISKDSVK (1)	gi|206583959|gb|aao90485.2| +6	7.5e3	0.62	9566
(K)	AHQSFK (1)	gi|206583959|gb|aao90485.2| +14	4.2e3	0.38	3921
(R)	DVWSQLSK (1)	gi|206583959|gb|aao90485.2| +14	7.5e3	0.61	10580
(K)	VDGHVAEVVGRL (1)	gi|206583959|gb|aao90485.2| +14	8.4e3	0.92	10034
(K)	VILYFYPK (1)	gi|206583959|gb|aao90485.2| +14	4.6e3	0.41	12198
(K)	VKVDGHVAEVVGR (1)	gi|206583959|gb|aao90485.2| +14	7.8e3	0.52	7508
(K)	VKVDGHVAEVVGRL (1)	gi|206583959|gb|aao90485.2| +14	5.6e3	0.91	10542
(K)	YKGIER (1)	gi|206583959|gb|aao90485.2| +14	1.6e4	0.36	5077

Au gi|206583799|gb|AAO89774.2| **MS / MS Spectra:** 5 **Sum TIC:** 6.0e4 **Avg TIC:** 1.2e4 **Cov:** 3.9% **Uniq:** 2
peptidase, C40 family [Coxiella burnetii RSA 493]

(P1)	**Sequence (count)**	**Reference (+addnl)**	**Σ TIC**	**Sf_{max}**	**Scans**
(R)	GIAM*TNLNAR (1)	gi|206583799|gb|aao89774.2| +11	1.4e4	0.87	6035
(R)	GIAMTNLNAR (1)	gi|206583799|gb|aao89774.2| +11	4.7e3	0.91	8432
(K)	TNVIDDQDQFR (3)	gi|206583799|gb|aao89774.2| +11	4.1e4	0.97	7311-8289

Av gi|29540670|gb|AAO89614.1| **MS / MS Spectra:** 6 **Sum TIC:** 5.9e4 **Avg TIC:** 9.9e3 **Cov:** 19.4% **Uniq:** 6
outer membrane protein [Coxiella burnetii RSA 493]

(P1)	**Sequence (count)**	**Reference (+addnl)**	**Σ TIC**	**Sf_{max}**	**Scans**
(R)	FFLPQTTTVK (1)	gi|29540670|gb|aao89614.1| +13	9.0e3	0.52	8977
(Y)	LHNYKR (1)	gi|29540670|gb|aao89614.1| +13	4.6e3	0.70	3018
(A)	LYLHNYK (1)	gi|29540670|gb|aao89614.1| +13	3.1e3	0.13	5946
(A)	LYLHNYKR (1)	gi|29540670|gb|aao89614.1| +13	1.4e4	0.91	5584

(R) THIVHYPIK (1)	gi|29540670|gb|aao89614.1| +13	2.2e4	0.92	5668
(R) VTTPLTHAR (1)	gi|29540670|gb|aao89614.1| +13	6.2e3	0.24	4974

Aw gi|29541213|gb|AAO90156.1| **MS/MS Spectra:** 9 **Sum TIC:** 5.7e4 **Avg TIC:** 6.3e3 **Cov:** 37.0% **Uniq:** 7
outer membrane protein [Coxiella burnetii RSA 493]

(P1) Sequence (count)	Reference (+addnl)	Σ TIC	Sf_{max}	Scans
(K) EIQNDESTLR (2)	gi|29541213|gb|aao90156.1| +13	1.3e4	0.87	4905-4906
(K) LKRDEAVM*GK (1)	gi|29541213|gb|aao90156.1| +13	6.6e3	0.78	4116
(K) LKRDEAVM*GKK (1)	gi|29541213|gb|aao90156.1| +13	1.1e4	0.32	3904
(K) LTQSLQQNLQK (2)	gi|29541213|gb|aao90156.1| +13	1.3e4	0.92	5169-6124
(R) QQQQQFQQELFVAQNK (1)	gi|29541213|gb|aao90156.1| +13	3.9e3	0.77	10121
(K) VAERENLD (1)	gi|29541213|gb|aao90156.1| +13	3.8e3	0.46	3977
(K) VAERENLDLVLPK (1)	gi|29541213|gb|aao90156.1| +13	5.6e3	0.93	10391

Ax gi|29541711|gb|AAO90650.1| **MS/MS Spectra:** 10 **Sum TIC:** 5.4e4 **Avg TIC:** 5.4e3 **Cov:** 49.5% **Uniq:** 8
enhanced entry protein [Coxiella burnetii RSA 493]

(P1) Sequence (count)	Reference (+addnl)	Σ TIC	Sf_{max}	Scans
(R) DLENEGSLL (1)	gi|29541711|gb|aao90650.1| +13	7.8e3	0.17	9050
(T) IYFIHNVSK (2)	gi|29541711|gb|aao90650.1| +13	9.7e3	0.91	7150-7667
(K) LKIINCSKVLDICEFPRTRFGDNNRGN (1)	gi|29541711|gb|aao90650.1| +13	3.1e3	0.12	9108
(R) SNDEPYIIHI (1)	gi|29541711|gb|aao90650.1| +13	2.7e3	0.45	10028
(A) VENIGSSTAAVSAIR (2)	gi|29541711|gb|aao90650.1| +13	1.5e4	0.97	7022-7812
(K) VLDICEFPR (1)	gi|29541711|gb|aao90650.1| +13	5.8e3	0.93	9245
(Y) YNLILQPTGR (1)	gi|29541711|gb|aao90650.1| +13	4.8e3	0.42	8554
(R) YPYGCYPMGYK (1)	gi|29541711|gb|aao90650.1| +13	5.0e3	0.89	7914

Ay gi|29542580|gb|AAO91514.1| **MS/MS Spectra:** 8 **Sum TIC:** 4.7e4 **Avg TIC:** 5.9e3 **Cov:** 42.1% **Uniq:** 6
hypothetical exported protein [Coxiella burnetii RSA 493]

(P1) Sequence (count)	Reference (+addnl)	Σ TIC	Sf_{max}	Scans
(K) AGASDQDLK (1)	gi|29542580|gb|aao91514.1| +13	1.1e4	0.89	3414
(K) AGASDQDLKEI (1)	gi|29542580|gb|aao91514.1| +13	6.9e3	0.71	7025
(S) FNAFPSM*SPLSK (1)	gi|29542580|gb|aao91514.1| +13	6.2e3	0.53	8278
(S) FNAFPSMSPLSK (1)	gi|29542580|gb|aao91514.1| +13	5.1e3	0.22	9863
(R) GISFNAFPSM*SPLSK (2)	gi|29542580|gb|aao91514.1| +13	1.0e4	0.76	9865-10880
(K) NKDQFFTAIRSEVE (1)	gi|29542580|gb|aao91514.1| +13	3.8e3	0.83	9448
(I) SFNAFPSM*SPLSK (1)	gi|29542580|gb|aao91514.1| +13	4.1e3	0.61	8521

Az gi|29542118|gb|AAO91055.1| **MS/MS Spectra:** 3 **Sum TIC:** 4.7e4 **Avg TIC:** 1.6e4 **Cov:** 38.5% **Uniq:** 3
hypothetical protein CBU_1558 [Coxiella burnetii RSA 493]

(P1) Sequence (count)	Reference (+addnl)	Σ TIC	Sf_{max}	Scans
(K) CGEQTEVIQK (1)	gi|29542118|gb|aao91055.1| +13	2.4e4	0.91	5928
(R) DKDNKPKPEAASKPEK (1)	gi|29542118|gb|aao91055.1| +13	5.6e3	0.26	2784
(M) PIYEYQCEK (1)	gi|29542118|gb|aao91055.1| +13	1.7e4	0.90	7900

Ba gi|29541765|gb|AAO90704.1| **MS/MS Spectra:** 3 **Sum TIC:** 4.6e4 **Avg TIC:** 1.5e4 **Cov:** 59.0% **Uniq:** 3
bacterial protein translation initiation factor 1 (IF-1) [Coxiella burnetii RSA 493]

(P1) Sequence (count)	Reference (+addnl)	Σ TIC	Sf_{max}	Scans
(M) AKEESIEM*QGTVVDSLPNTTFR (1)	gi|29541765|gb|aao90704.1| +16	1.4e4	0.93	10454
(R) ILTGDAVTVELTPYDLTR (1)	gi|29541765|gb|aao90704.1| +14	2.3e4	0.97	12946
(K) KPPTSKAEE (1)	gi|29541765|gb|aao90704.1| +16	9.7e3	0.69	3435

Bb uc|P81605|DCD_HUMAN **MS/MS Spectra:** 8 **Sum TIC:** 4.6e4 **Avg TIC:** 5.8e3 **Cov:** 42.7% **Uniq:** 5
CON Dermcidin precursor - Homo sapiens

(P1) Sequence (count)	Reference (+addnl)	Σ TIC	Sf_{max}	Scans
(K) ENAGEDPGLAR (1)	uc|p81605|dcd_human	3.4e3	0.79	3604
(K) GAVHDVKDVLDSVL (1)	uc|p81605|dcd_human	5.0e3	0.34	11024
(K) KAVGGLGK (1)	uc|p81605|dcd_human	4.4e3	0.40	3305
(K) LGKDAVEDLESVGK (4)	uc|p81605|dcd_human	2.5e4	0.97	7400-9433
(A) VEDLESVGK (1)	uc|p81605|dcd_human	8.2e3	0.58	7098

Bc gi|29540981|gb|AAO89924.1| **MS/MS Spectra:** 7 **Sum TIC:** 4.5e4 **Avg TIC:** 6.4e3 **Cov:** 50.7% **Uniq:** 7
hypothetical membrane associated protein [Coxiella burnetii RSA 493]

(P1) Sequence (count)	Reference (+addnl)	Σ TIC	Sf_{max}	Scans
(G) AAVGSAVTSR (1)	gi|29540981|gb|aao89924.1| +13	3.9e3	0.49	4111
(A) AVGSAVTSR (1)	gi|29540981|gb|aao89924.1| +13	7.9e3	0.86	3831
(A) AVGSAVTSRR (1)	gi|29540981|gb|aao89924.1| +13	9.8e3	0.67	3768
(A) GAAVGSAVTSR (1)	gi|29540981|gb|aao89924.1| +13	5.6e3	0.89	4233
(K) TGAVVGGVAGAAVGSAVTSR (1)	gi|29540981|gb|aao89924.1| +13	7.2e3	0.98	7496
(A) TTGAAVGGITGAALGGK (1)	gi|29540981|gb|aao89924.1| +9	4.3e3	0.88	7358
(V) VGGVAGAAVGSAVTSRR (1)	gi|29540981|gb|aao89924.1| +13	6.6e3	0.27	6173

Bd gi|120575839|gb|EAX32463.1| **MS/MS Spectra:** 3 **Sum TIC:** 4.4e4 **Avg TIC:** 1.5e4 **Cov:** 17.3% **Uniq:** 2
putative endoribonuclease L-PSP [Coxiella burnetii 'MSU Goat Q177']

(P1)	Sequence (count)	Reference (+addnl)	Σ TIC	Sf_{max}	Scans
(K)	HYYEEPYPAR (2)	gi|120575839|gb|eax32463.1| +11	1.8e4	0.85	6089-6094
(-)	M*KQIIGTNKAPR (1)	gi|120575839|gb|eax32463.1| +3	2.6e4	0.87	5699

Be gi|29540650|gb|AAO89594.1| **MS/MS Spectra:** 3 **Sum TIC:** 4.0e4 **Avg TIC:** 1.3e4 **Cov:** 41.2% **Uniq:** 3
carbon storage regulator [Coxiella burnetii RSA 493]

(P1)	Sequence (count)	Reference (+addnl)	Σ TIC	Sf_{max}	Scans
(K)	APPILEEVE (1)	gi|29540650|gb|aao89594.1| +16	7.5e3	0.13	10833
(K)	HIAVHREEIYQR (1)	gi|29540650|gb|aao89594.1| +16	2.4e4	0.95	5148
(R)	IQEEKPKAPPILEEVE (1)	gi|29540650|gb|aao89594.1| +16	8.7e3	0.84	10164

Bf gi|206584056|gb|AAO90889.2| **MS/MS Spectra:** 3 **Sum TIC:** 4.0e4 **Avg TIC:** 1.3e4 **Cov:** 11.4% **Uniq:** 3
SSU ribosomal protein S2P [Coxiella burnetii RSA 493]

(P1)	Sequence (count)	Reference (+addnl)	Σ TIC	Sf_{max}	Scans
(K)	EVQQPIEASKAEAEEGK (1)	gi|206584056|gb|aao90889.2| +17	3.0e4	0.92	6759
(K)	KSEKPTTEKRPTK (1)	gi|206584056|gb|aao90889.2| +17	5.3e3	0.88	3628
(K)	VAEKAQAAAEKK (1)	gi|206584056|gb|aao90889.2| +17	4.4e3	0.85	4060

Bg gi|29542236|gb|AAO91173.1| **MS/MS Spectra:** 6 **Sum TIC:** 3.6e4 **Avg TIC:** 6.1e3 **Cov:** 31.9% **Uniq:** 5
hypothetical cytosolic protein [Coxiella burnetii RSA 493]

(P1)	Sequence (count)	Reference (+addnl)	Σ TIC	Sf_{max}	Scans
(K)	HLISEEKDAEK (2)	gi|29542236|gb|aao91173.1| +13	1.5e4	0.95	4976-5022
(R)	KLFDTLCAM*LIR (1)	gi|29542236|gb|aao91173.1| +11	2.7e3	0.77	12508
(K)	KVNEHQAWEK (1)	gi|29542236|gb|aao91173.1| +13	4.2e3	0.83	5607
(-)	M*DAIDFLTKEHEK (1)	gi|29542236|gb|aao91173.1| +13	4.0e3	0.43	10339
(K)	VNEHQAWEK (1)	gi|29542236|gb|aao91173.1| +13	1.0e4	0.27	6069

Bh gi|206584216|gb|AAO91564.2| **MS/MS Spectra:** 4 **Sum TIC:** 3.6e4 **Avg TIC:** 9.0e3 **Cov:** 9.4% **Uniq:** 3
uroporphyrin-III C-methyltransferase [Coxiella burnetii RSA 493]

(P1)	Sequence (count)	Reference (+addnl)	Σ TIC	Sf_{max}	Scans
(A)	ALSM*QPHR (1)	gi|206584216|gb|aao91564.2| +13	7.1e3	0.48	3172
(R)	SAPESPTTSTPESK (2)	gi|206584216|gb|aao91564.2| +13	2.2e4	0.88	3906-4045
(K)	VYQGSLNR (1)	gi|206584216|gb|aao91564.2| +13	6.6e3	0.79	4481

Bi gi|29541249|gb|AAO90192.1| **MS/MS Spectra:** 2 **Sum TIC:** 3.4e4 **Avg TIC:** 1.7e4 **Cov:** 11.3% **Uniq:** 2
6,7-dimethyl-8-ribityllumazine synthase [Coxiella burnetii RSA 493]

(P1)	Sequence (count)	Reference (+addnl)	Σ TIC	Sf_{max}	Scans
(K)	EIEITDE (1)	gi|29541249|gb|aao90192.1| +18	1.9e4	0.76	8539
(K)	LAIVVSQFNR (1)	gi|29541249|gb|aao90192.1| +18	1.5e4	0.96	10816

Bj uc|547751|sp|Q04695|K1CQ_HUMAN **MS/MS Spectra:** 2 **Sum TIC:** 2.4e4 **Avg TIC:** 1.2e4 **Cov:** 5.3% **Uniq:** 2
CON Keratin, type i cytoskeletal 17 (cytokeratin 17) (K17) (CK 17) (39.1) (VERSION 1) gi|422802|pir||S30433 keratin 17, cytoskeletal - human gi|30379 (Z19574) cytokeratin 17 [Homo sapiens] gi|34075 (X62571) keratin related product [Homo sapiens]

(P1)	Sequence (count)	Reference (+addnl)	Σ TIC	Sf_{max}	Scans
(R)	ALEEANTELEVK (1)	uc|547751|sp|q04695|k1cq_huma +1	1.4e4	0.97	8228
(R)	LSGGLGAGSCR (1)	uc|547751|sp|q04695|k1cq_huma +1	1.0e4	0.92	6136

Bk uc|Q02413|DSG1_HUMAN **MS/MS Spectra:** 2 **Sum TIC:** 2.1e4 **Avg TIC:** 1.1e4 **Cov:** 2.5% **Uniq:** 2
CON Desmoglein-1 OS=Homo sapiens GN=DSG1 PE=1 SV=1

(P1)	Sequence (count)	Reference (+addnl)	Σ TIC	Sf_{max}	Scans
(R)	ESSNVVVTER (1)	uc|q02413|dsg1_human	1.6e4	0.90	5974
(R)	YVM*GNNPADLLAVDSR (1)	uc|q02413|dsg1_human	4.9e3	0.95	10511

Bl gi|29541138|gb|AAO90081.1| **MS/MS Spectra:** 2 **Sum TIC:** 1.2e4 **Avg TIC:** 5.8e3 **Cov:** 7.1% **Uniq:** 2
hypothetical protein CBU_0535 [Coxiella burnetii RSA 493]

(P1)	Sequence (count)	Reference (+addnl)	Σ TIC	Sf_{max}	Scans
(R)	AAAQGQPLAIR (1)	gi|29541138|gb|aao90081.1| +13	4.8e3	0.85	6045
(K)	AAIQQLPQPLQK (1)	gi|29541138|gb|aao90081.1| +11	6.7e3	0.81	7341

Bm gi|29540652|gb|AAO89596.1| **MS/MS Spectra:** 2 **Sum TIC:** 1.1e4 **Avg TIC:** 5.5e3 **Cov:** 8.6% **Uniq:** 2
ribose 5-phosphate isomerase [Coxiella burnetii RSA 493]

(P1)	Sequence (count)	Reference (+addnl)	Σ TIC	Sf_{max}	Scans
(K)	AAAM*EAIQFVK (1)	gi|29540652|gb|aao89596.1| +18	5.3e3	0.86	8994
(K)	LKGDPVYR (1)	gi|29540652|gb|aao89596.1| +18	5.7e3	0.44	5686

Bn uc|P15924|DESP_HUMAN **MS/MS Spectra:** 2 **Sum TIC:** 1.1e4 **Avg TIC:** 5.4e3 **Cov:** 0.8% **Uniq:** 2
CON Desmoplakin - Homo sapiens

(P1)	Sequence (count)	Reference (+addnl)	Σ TIC	Sf_{max}	Scans
(K)	NQCTQVVQER (1)	uc|p15924|desp_human	4.7e3	0.82	5596

(R) YEVTSGGGGTSR (1)	uc|p15924|desp_human	6.1e3	0.91	5390

Bo gi|29541890|gb|AAO90828.1| **MS/MS Spectra:** 2 **Sum TIC:** 1.1e4 **Avg TIC:** 5.3e3 **Cov:** 32.8% **Uniq:** 2
LSU ribosomal protein L35P [Coxiella burnetii RSA 493]

(P1) **Sequence (count)**	**Reference (+addnl)**	**Σ TIC**	**Sf_{max}**	**Scans**
(R) AASNHNHILTK (1)	gi|29541890|gb|aao90828.1| +13	4.5e3	0.91	3226
(K) IHEVAPSDM*R (1)	gi|29541890|gb|aao90828.1| +16	6.0e3	0.86	3831

Bp gi|29542459|gb|AAO91393.1| **MS/MS Spectra:** 2 **Sum TIC:** 8.0e3 **Avg TIC:** 4.0e3 **Cov:** 5.4% **Uniq:** 2
peptidase, M16 family [Coxiella burnetii RSA 493]

(P1) **Sequence (count)**	**Reference (+addnl)**	**Σ TIC**	**Sf_{max}**	**Scans**
(K) KLQTDPVSEEELKR (1)	gi|29542459|gb|aao91393.1| +13	3.3e3	0.95	6527
(R) LSADQLPVAFR (1)	gi|29542459|gb|aao91393.1| +13	4.7e3	0.95	10271

Single, ungrouped sequences **MS/MS Spectra:** 35 **Sum TIC:** 3.3e5 **Avg TIC:** 9.6e3

(P1) **Sequence (count)**	**Reference (+addnl)**	**Σ TIC**	**Sf_{max}**	**Scans**
Bq (R) AANASPEDEKK (1)	gi|73621787|sp|q83d39.1|rl31_ +17	1.0e4	0.87	3618
RecName: Full=50S ribosomal protein L31				
Br (R) ASGGAYGCPK (1)	gi|29654687|ref|np_820379.1| +1	1.5e4	0.93	3592
enhanced entry protein [Coxiella burnetii RSA 493]				
Bs (R) CIQTLCRR (1)	gi|212218621|ref|yp_002305408 +13	7.5e3	0.89	7218
ATP-dependent clp protease ATP-binding subunit [Coxiella burnetii CbuK_Q154]				
Bt (K) DGCNACALAGTVK (1)	gi|212217843|ref|yp_002304630 +13	6.4e3	0.96	7252
hypothetical protein CbuK_0168 [Coxiella burnetii CbuK_Q154]				
Bu (K) DINEEPQPSTSVSK (1)	gi|212212073|ref|yp_002303009 +11	5.6e3	0.90	6105
hypothetical membrane spanning protein [Coxiella burnetii CbuG_Q212]				
Bv (K) DSGAETCHPM* (1)	gi|212218481|ref|yp_002305268 +13	7.5e3	0.91	4259
hypothetical protein CbuK_0894 [Coxiella burnetii CbuK_Q154]				
Bw (R) DVLANAM*GENNH (1)	gi|212218924|ref|yp_002305711 +13	5.0e3	0.93	5145
hypothetical protein CbuK_1391 [Coxiella burnetii CbuK_Q154]				
Bx (R) DVLANAMGENNH (1)	gi|212218924|ref|yp_002305711 +13	1.0e4	0.90	7668
hypothetical protein CbuK_1391 [Coxiella burnetii CbuK_Q154]				
By (R) EIDEQITLDDAGVM*K (1)	gi|212219305|ref|yp_002306092 +11	7.6e3	0.97	9433
GatB/Yqey domain protein [Coxiella burnetii CbuK_Q154]				
Bz (R) EIDEQITLDDAGVMK (1)	gi|212219305|ref|yp_002306092 +11	4.6e3	0.96	10655
GatB/Yqey domain protein [Coxiella burnetii CbuK_Q154]				
Ca (K) FGIINPETIVVQAPR (1)	gi|212218977|ref|yp_002305764 +11	2.5e3	0.88	11117
hypothetical protein CbuK_1450 [Coxiella burnetii CbuK_Q154]				
Cb (R) GAM*LSKDPTTGETHLR (1)	gi|51316810|sp|q83e41.1|rl32_ +18	7.2e3	0.95	4803
RecName: Full=50S ribosomal protein L32				
Cc (R) HSQVGQGESAGSK (1)	uc|p20930|fila_human	1.1e4	0.93	3748
CON Filaggrin OS=Homo sapiens GN=FLG PE=1 SV=3				
Cd (R) HVAAGTQQPYTDGVR (1)	uc|p14923|plak_human	3.7e3	0.89	6264
CON Junction plakoglobin - Homo sapiens (Human).				
Ce (K) IDDQLVQNMSLPEAVSR (1)	gi|212219251|ref|yp_002306038 +15	6.9e4	0.87	11757
carboxy-terminal processing protease precursor [Coxiella burnetii CbuK_Q154]				
Cf (R) IQGHPLVKR (1)	gi|207081599|gb|abs76531.2| +1	4.4e3	0.90	3993
hypothetical protein CBUD_0079 [Coxiella burnetii Dugway 5J108-111]				
Cg (K) ITTVQQAIDYIK (2)	gi|212218897|ref|yp_002305684 +11	2.4e4	0.96	11978-12237
acyl carrier protein [Coxiella burnetii CbuK_Q154]				
Ch (R) KVPQVSTPTLVEVSR (2)	uc|p02769|albu_bovin +1	1.5e4	0.91	7104-9316
CON serum albumin precursor [Bos taurus]				
Ci (K) LQQEQLNEQQR (1)	gi|212219325|ref|yp_002306112 +13	7.9e3	0.97	4628
IcmE [Coxiella burnetii CbuK_Q154]				
Cj (K) NLGGYM*NCVSHDQK (1)	gi|212219566|ref|yp_002306353 +13	2.0e4	0.92	6704
hypothetical protein CbuK_2117 [Coxiella burnetii CbuK_Q154]				
Ck (K) NTGVQAAVVADK (1)	gi|30142092|gb|aap13856.1| +23	4.7e3	0.86	5266
heat shock protein B [Coxiella burnetii]				
Cl (K) NVGLESWDYLK (1)	gi|212218791|ref|yp_002305578 +13	8.3e3	0.92	12553
ferredoxin, 2Fe-2s [Coxiella burnetii CbuK_Q154]				
Cm (R) RGPLLIHQFLK (1)	gi|212219435|ref|yp_002306222 +13	8.1e3	0.88	9395

phosphoglycerol transferase MdoB-like protein, alkaline phosphatase superfamily [Coxiella burnetii CbuK_Q154]

Cn (R) RLQLFPR (1)<br>*CON* Keratinocyte proline-rich protein OS=Homo sapiens GN=KPRP PE=1 SV=1	uc|q5t749|kprp_human	1.2e4	0.81	10150
Co (R) RVPIQDLVGHSPEVIAVDDKGR (1)<br>replicative DNA helicase [Coxiella burnetii CbuK_Q154]	gi|212218348|ref|yp_002305135 +13	9.5e3	0.97	10416
Cp (R) SASANTAALR (1)<br>Hq1 [Coxiella burnetii]	gi|1448971|gb|aab36614.1| +15	8.4e3	0.83	4143
Cq (K) SFDINDVNFANR (1)<br>RecName: Full=UPF0422 protein CBU_0937; Flags: Precursor	gi|81629087|sp|q83d09.1|y937_ +12	6.0e3	0.91	9239
Cr (R) SLITDPAQNSPSTQPR (1)<br>IcmK [Coxiella burnetii CbuK_Q154]	gi|212219326|ref|yp_002306113 +13	3.5e3	0.92	6998
Cs (R) SQIVNDLPLGR (1)<br>thioredoxin peroxidase [Coxiella burnetii CbuK_Q154]	gi|212217972|ref|yp_002304759 +13	4.7e3	0.95	9684
Ct (R) TDM*GGEQAPLDVISSVR (1)<br>short chain dehydrogenase [Coxiella burnetii CbuK_Q154]	gi|212218470|ref|yp_002305257 +11	6.3e3	0.94	10494
Cu (R) THAENLKR (1)<br>rare lipoprotein A [Coxiella burnetii CbuK_Q154]	gi|212218446|ref|yp_002305233 +13	1.1e4	0.87	2931
Cv (R) VAAVKER (1)<br>DNA-binding protein [Coxiella burnetii CbuK_Q154]	gi|212218921|ref|yp_002305708 +11	6.1e3	0.87	2909
Cw (K) VLQAGQGQSPTLNDEVTVNYEGR (1)<br>peptidyl propyl cis/trans isomerase [Coxiella burnetii]	gi|538149|gb|aaa92352.1| +14	2.2e3	0.93	9432

**Dir:** aomslandrml01to10-gt_cbrpt		**Files:** 101015Yaorml01-gt (10/21/10-10/21/10)	**Enz:** Trypsin	
**Oper:** wsl	**Date:** 10/22/10	**Report:** RML01TO10-GTscb (wsl)	R2968/D1741/C12<br>**MR:** 1	**FDR:** n/a%

Legend:

Sequence: The isobaric residue pairs Leu/IIe, Gln/Lys and Phe/Msx are displayed with the assignment as in the known sequence. Either residue within the pair may be possible: the displayed assignment does not connote a defined assignment to one or the other. The amino acid N-terminal to the known sequence is displayed in parentheses ().

Ions: The number of fragment ions (b-, y- and/or a-ions) experimentally observed / number of fragment ions possible. This fraction is a crude estimate of the minimum percentage of the sequence represented by the spectrum.

Reference: The database reference which contains the displayed sequence. A reference followed by a plus sign and number (e.g. +2) indicates the displayed sequence is also present in that number of additional database references

TIC: The intensity (total ion current) of the MS/MS spectrum. This is a unitless number. Note: mass spectrometry of different peptide analytes is not quantitative, because each peptide's ionization is dependent on structure. Caution should be used in using these values to ascertain major vs. minor components.

Histograms: Each set of peptide sequences grouped A, B, C...etc. by common database identifier have three values in their header: Sum TIC, Avg TIC and number of MS/MS Spectra. Histograms plot the percent of the total that each of these is.

Sum TIC: Sum of all peptides' TIC in the group
Avg TIC: Avgerage of all peptides' TIC in the group
MS/MS Spectra: Number of peptides in the group

Scan: The scan number(s) of the acquired MS/MS spectrum.

Modifications:

* = +15.9949 on the preceding M
The residue weight used for C was 160.03064
