# Supplementary figures and images for: Sec-mediated secretion by Coxiella burnetii
Source: BMC Microbiol. 2013 Oct 5;13:222. doi: 10.1186/1471-2180-13-222 (PMC3882888; doi:10.1186/1471-2180-13-222)

Additional file 3

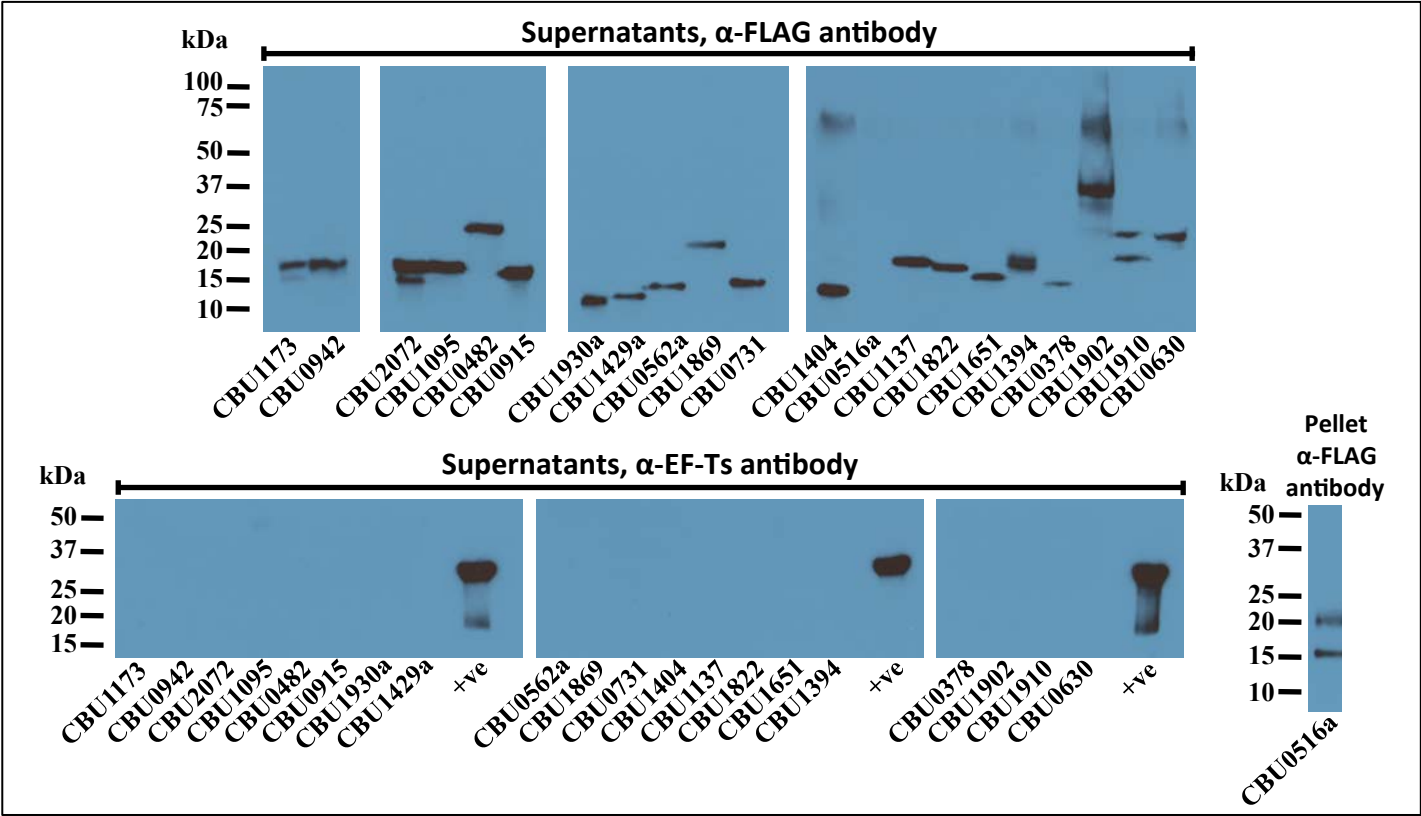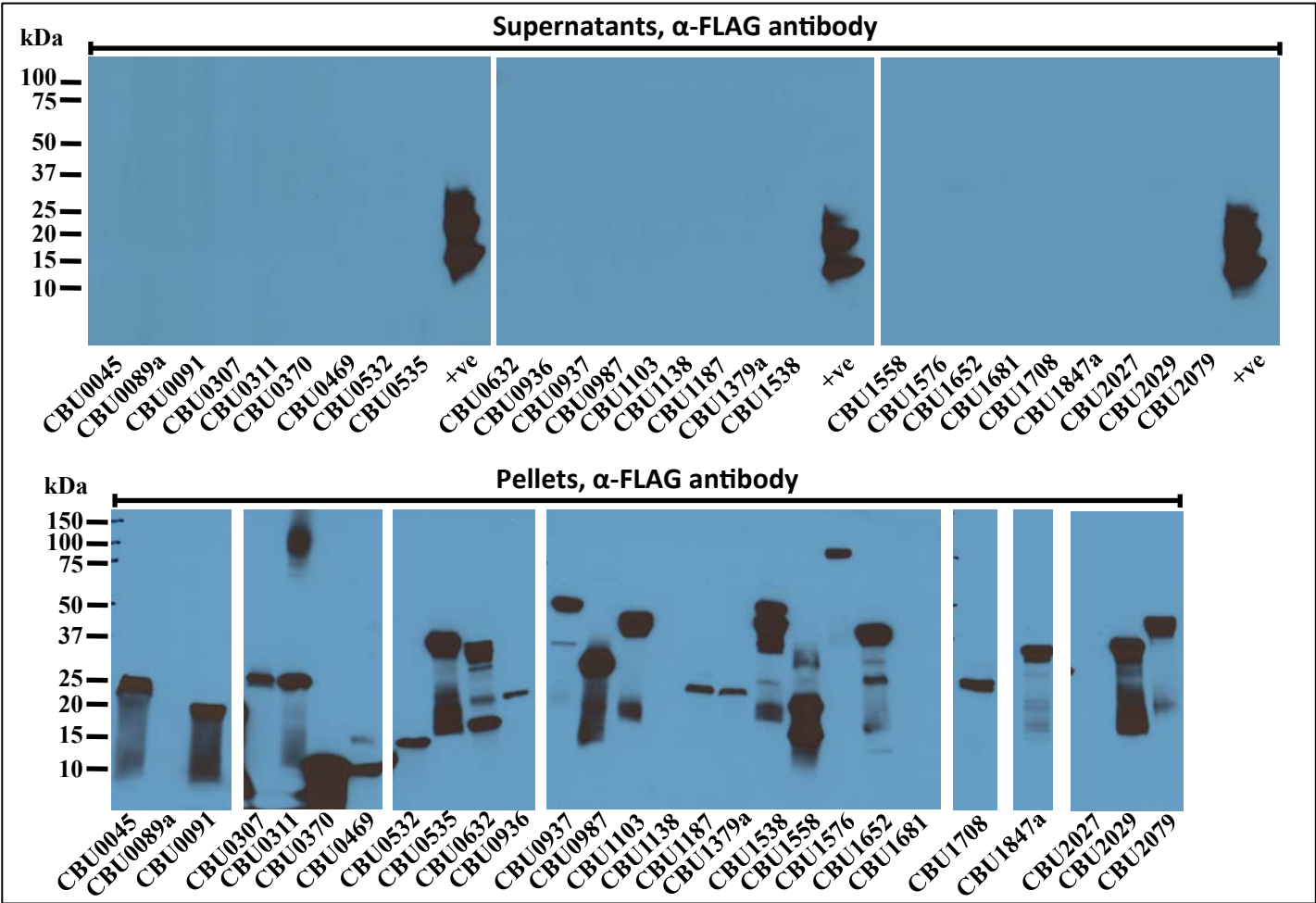

Supplement: Additional file 3 — Expression of FLAG-tagged secretion candidates by C. burnetii transformants to confirm secretion.C. burnetii transformed with plasmids encoding FLAG-tagged secretion candidates were cultured for 48 h, then expression of tagged protein induced by addition of aTc for 24 h. Supernatants were harvested, TCA precipitated and analyzed by immunoblotting using antibody directed against the FLAG-tag. Supernatants of samples that were positive for secretion were then probed using antibody directed against EF-Ts to rule out cell lysis as a source of protein present in supernatants. Whole cell lysate of C. burnetii expressing FLAG-tagged CBU1764a was used as a positive control (+ve). To confirm that proteins not present in supernatants were expressed by C. burnetii transformants, lysates of bacterial pellets were probed with antibody directed against the FLAG-tag. [file 1471-2180-13-222-S3.pdf]

Additional file 5

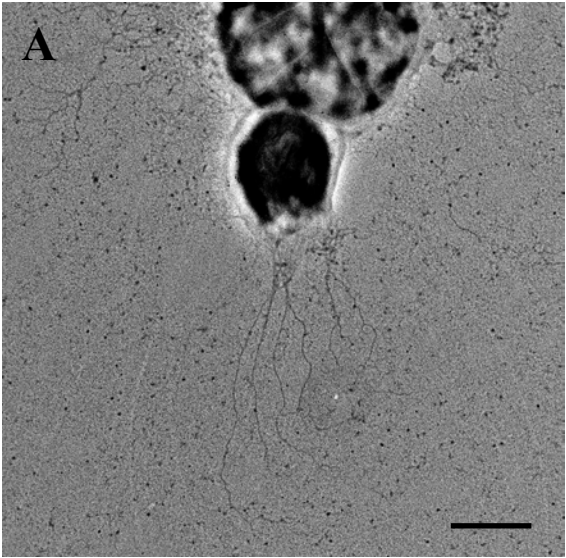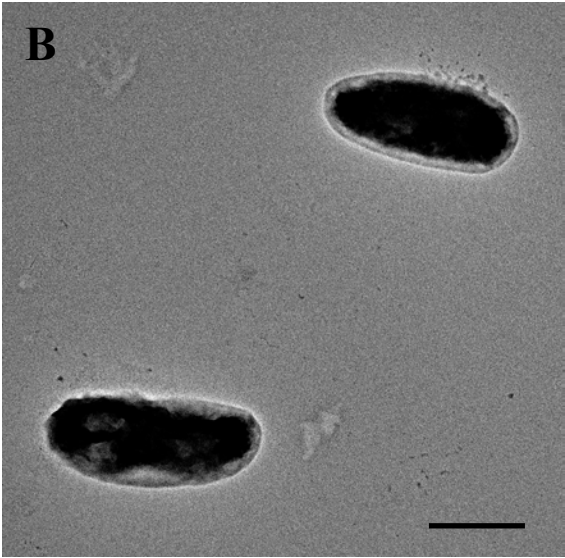

Supplement: Additional file 5 — C. burnetii is not pilliated. Transmission electron micrographs of negatively stained bacteria show pili on F. tularensis LVS (panel A) but not C. burnetii (panel B). Scale bars = 0.5 μm. [file 1471-2180-13-222-S5.pdf]
