# Supplementary material for: Sec-mediated secretion by Coxiella burnetii
Source: BMC Microbiol. 2013 Oct 5;13:222. doi: 10.1186/1471-2180-13-222 (PMC3882888; doi:10.1186/1471-2180-13-222)
Supplement: Additional file 4 — Comparison of F. novicida and C. burnetii pil genes. The C. burnetii genome contains 13 pil genes, 11 of which are also present in the F. novicida genome, a bacterium that employs T4P-mediated secretion. [file 1471-2180-13-222-S4.pdf]

Additional file 4

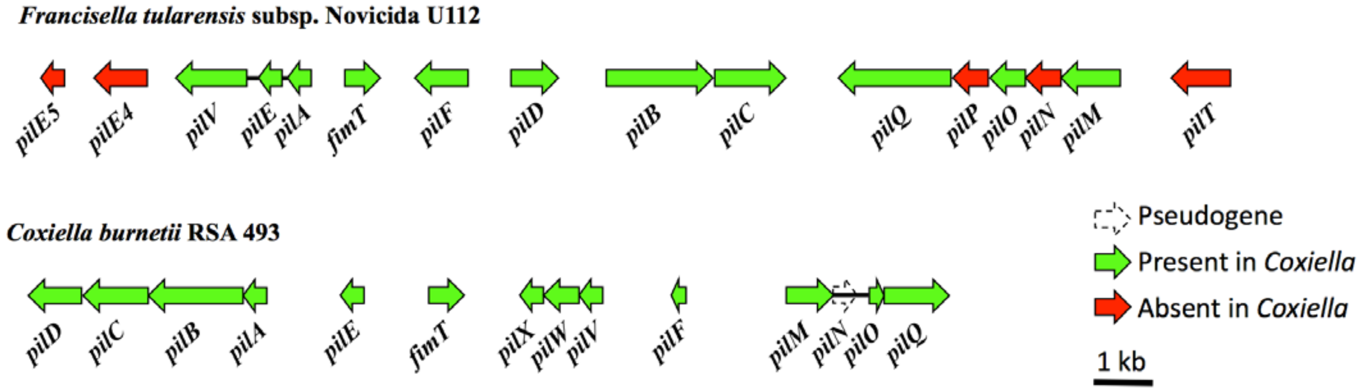

Although not annotated in the *C. burnetii* RSA493 genome the *pilM*, *pilO*, *pilX*, *pilW* & *pilV* genes were identified using either a BLAST or DELTA-BLAST search directed against Pseudomonas aeruginosa PA01 proteins. Gene numbers: *pilA* - CBU0156, FTN0415; *pilB* - CBU0155, FTN1115; *pilC* - CBU0154, FTN1116; *pilD* - CBU0153, FTN1000; *pilE* - CBU0412, FTN0414; *pilE4* - FTN0389; *pilE5* - FTN 0070; *pilF* - CBU1855, FTN0946; *pilM* - CBU1888, FTN1141; *pilN* - CBU1889, FTN1140; *pilO* - CBU1890, FTN1139; *pilP* - FTN1138; *pilQ* - CBU1891, FTN1137; *pilT* - FTN1622; *pilV* - CBU1481, FTN0413; *pilW* - CBU1480; *pilX* - CBU1479; *fimT* - CBU0453, FTN0664.
C. burnetii pilA, *B*, *C* & *D*, *pilV*, *W* & *X* and *pilM, N*, *O* & *Q* are predicted operons [1].

1. Mao F, Dam P, Chou J, Olman V, Xu Y: **DOOR: a database for prokaryotic operons**. *Nucleic Acids Res* 2009, **37**:D459-463.
