# Supplementary material for: Sec-mediated secretion by Coxiella burnetii
Source: BMC Microbiol. 2013 Oct 5;13:222. doi: 10.1186/1471-2180-13-222 (PMC3882888; doi:10.1186/1471-2180-13-222)
Supplement: Additional file 6 — Primers used in this study. [file 1471-2180-13-222-S6.pdf]

Additional file 6 - Primers used in this study

Primer name	Sequence (5′ to 3′)
**pJB-CAT-TetRA-3xFLAG construction**	
TetRA-pJB-F	CAGGAAACAGAATTCTTAAGACCCACTTTCACATTTAAG
TetRA-3xFLAG-R	TCTTTGTAGTCCATCTGCAGCTTTTCTCTATCACTGATAGGGAGTG
**pJB-CAT-TetRA-3xFLAG insert cloning**	
F45pJBTetCFLAG	GTGATAGAGAAAAGCTGCAGATGAACCCGTTATACCGTCA
R45pJBTetCFLAG	TCTTTGTAGTCCATCTGCAGATTCACTTGAATCATTACTC
F0089apJBTetCFLAG	GTGATAGAGAAAAGCTGCAGTTGGCGCAAAATCAAAATGG
R0089apJBTetCFLAG	TCTTTGTAGTCCATCTGCAGCTATCGCTTTAGAGACCTGC
F91pJBTetCFLAG	GTGATAGAGAAAAGCTGCAGATGAACAACTTAGTGAAAAA
R91pJBTetCFLAG	TCTTTGTAGTCCATCTGCAGATAAGCCTCATAAATTAAAT
F110pJBTetCFLAG	GTGATAGAGAAAAGCTGCAGATGACTATCATACGTAATGT
F110-SPpJBTetCFLAG	GTGATAGAGAAAAGCTGCAGATGAGTAGCAATGAAGCAAAGTA
R110pJBTetCFLAG	TCTTTGTAGTCCATCTGCAGTTTTTTTTGATACAAACATT
F307pJBTetCFLAG	GTGATAGAGAAAAGCTGCAGATGTCGGTAAAAAAACTAGT
R307pJBTetCFLAG	TCTTTGTAGTCCATCTGCAGCATTAAGAATTTGTAGCCAA
F311pJBTetCFLAG	GTGATAGAGAAAAGCTGCAGATGGAAACAACTACAAAACT
R311pJBTetCFLAG	TCTTTGTAGTCCATCTGCAGGATATTGAACAGGTAATTAA
F370pJBTetCFLAG	GTGATAGAGAAAAGCTGCAGATGACTACTTTATTCAAAAA
R370pJBTetCFLAG	TCTTTGTAGTCCATCTGCAGGTGGTAATGGCGATATCGAC
F378pJBTetCFLAG	GTGATAGAGAAAAGCTGCAGATGAGAAATTTCATTAAGAT
R378pJBTetCFLAG	TCTTTGTAGTCCATCTGCAGTTTTCTCAGGGATCGTGGGT
F400pJBTetCFLAG	GTGATAGAGAAAAGCTGCAGATGCGCTTATTAAAATTTTT
R400pJBTetCFLAG	TCTTTGTAGTCCATCTGCAGTTTAGTGGTAGAGAAAAGAC
F469pJBTetCFLAG	GTGATAGAGAAAAGCTGCAGATGATTATCATTGAAACCTA
R469pJBTetCFLAG	TCTTTGTAGTCCATCTGCAGGTGGTTATTTTCGCCCATGG
F482pJBTetCFLAG	GTGATAGAGAAAAGCTGCAGATGAAGAAAAATAATAATAG
R482pJBTetCFLAG	TCTTTGTAGTCCATCTGCAGTTCCTGCCCGAAATATTTCT
F0516apJBTetCFLAG	GTGATAGAGAAAAGCTGCAGATGAATGGAATGTTAAAGGG
R0516apJBTetCFLAG	TCTTTGTAGTCCATCTGCAGTCGATGAGTTAGTGATTTAG
F532pJBTetCFLAG	GTGATAGAGAAAAGCTGCAGATGAAAAAAATAATTCAATT
R532pJBTetCFLAG	TCTTTGTAGTCCATCTGCAGCGGATTAACCACTAAGCGAT
F535pJBTetCFLAG	GTGATAGAGAAAAGCTGCAGATGAAGCTGGTTGAACATTT
R535pJBTetCFLAG	TCTTTGTAGTCCATCTGCAGAACTACCTTCCTTAGCTGAA
F0562apJBTetCFLAG	GTGATAGAGAAAAGCTGCAGATGAAACTTAAGTTAATTTC
R0562apJBTetCFLAG	TCTTTGTAGTCCATCTGCAGTTGATCGGGTGAATCAGAGC
F619pJBTetCFLAG	GTGATAGAGAAAAGCTGCAGATGAAAAAATTACTAGCTGG

R619pJBTetCFLAG	TCTTTGTAGTCCATCTGCAGTTTAAAGTATGTAACTTTTT
F630pJBTetCFLAG	GTGATAGAGAAAAGCTGCAGATGAAACGATTGATTTTACC
R630pJBTetCFLAG	TCTTTGTAGTCCATCTGCAGTTTCTTTTTTACAGAAATTA
F632pJBTetCFLAG	GTGATAGAGAAAAGCTGCAGATGACTAGCGAAAATTACCA
R632pJBTetCFLAG	TCTTTGTAGTCCATCTGCAGTTCACCCGAGTGGTGATCCA
F731pJBTetCFLAG	GTGATAGAGAAAAGCTGCAGATGAAACGGTATTCAAGTGT
R731pJBTetCFLAG	TCTTTGTAGTCCATCTGCAGATTATTTTTAATCTCAAATT
F915pJBTetCFLAG	GTGATAGAGAAAAGCTGCAGATGAATTTTATGAGAGTTTA
F915-SPpJBTetCFLAG	GTGATAGAGAAAAGCTGCAGATGGCTAAAACCGGCGAACAACA
R915pJBTetCFLAG	TCTTTGTAGTCCATCTGCAGCCACCTCAATAAGATACCAT
F936pJBTetCFLAG	GTGATAGAGAAAAGCTGCAGATGAAAATAGTCTTAGCTCT
R936pJBTetCFLAG	TCTTTGTAGTCCATCTGCAGAAAATTGTAACTTAAACCAC
F937pJBTetCFLAG	GTGATAGAGAAAAGCTGCAGATGACGTCCAAGCTGGTCAT
R937pJBTetCFLAG	TCTTTGTAGTCCATCTGCAGAAAATAAAGATCGAACTGTG
F942pJBTetCFLAG	GTGATAGAGAAAAGCTGCAGATGTTAAGACGTCTTTGTTT
R942pJBTetCFLAG	TCTTTGTAGTCCATCTGCAGCATCGGATGACAAGTCTCTG
F987pJBTetCFLAG	GTGATAGAGAAAAGCTGCAGGTGGATCTCACCATGAAAAA
R987pJBTetCFLAG	TCTTTGTAGTCCATCTGCAGCTCTTTCATTGTGATGATTT
F1095pJBTetCFLAG	GTGATAGAGAAAAGCTGCAGATGAATATTCGTTCCTTAGC
R1095pJBTetCFLAG	TCTTTGTAGTCCATCTGCAGGCCTTTGGAAAGGGTGATCT
F1103pJBTetCFLAG	GTGATAGAGAAAAGCTGCAGGTGATGGTGTTCATTAGGGT
R1103pJBTetCFLAG	TCTTTGTAGTCCATCTGCAGACCTGGAGCCGAACTGTAAA
F1135pJBTetCFLAG	GTGATAGAGAAAAGCTGCAGGTGAAAAAAAATAATTTTCT
F1135-SPpJBTetCFLAG	GTGATAGAGAAAAGCTGCAGATGACGTATTGCCCAAAAACTAA
R1135pJBTetCFLAG	TCTTTGTAGTCCATCTGCAGAGAACCAAATGGACAGTCTT
F1137pJBTetCFLAG	GTGATAGAGAAAAGCTGCAGATGGAAAAGGGTTGTAAAAA
R1137pJBTetCFLAG	TCTTTGTAGTCCATCTGCAGTAGCAGCGATCCTTCATTTT
F1138pJBTetCFLAG	GTGATAGAGAAAAGCTGCAGGTGAAGGCCATGGAAAGGGC
R1138pJBTetCFLAG	TCTTTGTAGTCCATCTGCAGCCTCCCACTATTCTCCATGG
F1173pJBTetCFLAG	GTGATAGAGAAAAGCTGCAGATGAAAAAACATTTTCGCTT
F1173-SPpJBTetCFLAG	GTGATAGAGAAAAGCTGCAGATGGACAAATTAACTAAAGAAGT
R1173pJBTetCFLAG	TCTTTGTAGTCCATCTGCAGCTTTTTATTCTTACACTTAT
F1187pJBTetCFLAG	GTGATAGAGAAAAGCTGCAGATGGGCGGTAGTTTAATAGC
R1187pJBTetCFLAG	TCTTTGTAGTCCATCTGCAGGCTAAAGTTCGGGTCGTTCT
F1379apJBTetCFLAG	GTGATAGAGAAAAGCTGCAGATGGAGCGAAGCGAAATACG
R1379apJBTetCFLAG	TCTTTGTAGTCCATCTGCAGTCTGAAGAAATAAGCTAATG
F1394pJBTetCFLAG	GTGATAGAGAAAAGCTGCAGGTGAAAATAACAGTAACTAC
R1394pJBTetCFLAG	TCTTTGTAGTCCATCTGCAGATAAGACTTAACCACCACCT
F1404pJBTetCFLAG	GTGATAGAGAAAAGCTGCAGATGGAGGGCACTATGCGACG
R1404pJBTetCFLAG	TCTTTGTAGTCCATCTGCAGAAAATACGAACAAAACGGTC

F1429apJBTetCFLAG	GTGATAGAGAAAAGCTGCAGATGAGGTTAAGTAAATTAGG
R1429apJBTetCFLAG	TCTTTGTAGTCCATCTGCAGCTTTTGTTTGCAGTGGAAAC
F1538pJBTetCFLAG	GTGATAGAGAAAAGCTGCAGATGAGCCTAAAAAGAAAGAT
R1538pJBTetCFLAG	TCTTTGTAGTCCATCTGCAGATGCTTAACCACCTGAAGAC
F1558pJBTetCFLAG	GTGATAGAGAAAAGCTGCAGATGCCAATTTACGAATATCA
R1558pJBTetCFLAG	TCTTTGTAGTCCATCTGCAGTTCGCTTTCTTGGTTTTTCG
F1576pJBTetCFLAG	GTGATAGAGAAAAGCTGCAGATGCGTCCAGAACATAAAAA
R1576pJBTetCFLAG	TCTTTGTAGTCCATCTGCAGATGTTTCATCGAATACACCA
F1651pJBTetCFLAG	GTGATAGAGAAAAGCTGCAGATGAATAAATATCTTTTAAT
R1651pJBTetCFLAG	TCTTTGTAGTCCATCTGCAGAAACCAGTGAATATTAGATG
F1652pJBTetCFLAG	GTGATAGAGAAAAGCTGCAGATGAAAAATTTTCGTGTTCT
R1652pJBTetCFLAG	TCTTTGTAGTCCATCTGCAGTTTCCCTATTTGGTCAATCA
F1681pJBTetCFLAG	GTGATAGAGAAAAGCTGCAGATGGAGGGGCTAATGAGGGT
R1681pJBTetCFLAG	TCTTTGTAGTCCATCTGCAGATACCTTCCTATCGCTTTGG
F1708pJBTetCFLAG	GTGATAGAGAAAAGCTGCAGATGGCTTTTGAATTACCGGA
R1708pJBTetCFLAG	TCTTTGTAGTCCATCTGCAGGGATTTGAAGTTTTTCATCA
F1764apJBTetCFLAG	GTGATAGAGAAAAGCTGCAGATGAAAAAAATAATCGCATC
R1764apJBTetCFLAG	TCTTTGTAGTCCATCTGCAGAAAGCATATTCTACTGGGAA
F1822pJBTetCFLAG	GTGATAGAGAAAAGCTGCAGATGCTATCAAGAATTAGTCT
R1822pJBTetCFLAG	TCTTTGTAGTCCATCTGCAGTTTAACAATCCCACACGCAA
F1843pJBTetCFLAG	GTGATAGAGAAAAGCTGCAGATGATGAAACGATTACTCTT
R1843pJBTetCFLAG	TCTTTGTAGTCCATCTGCAGGTGGCAGTTTTTATCCAAAA
F1847apJBTetCFLAG	GTGATAGAGAAAAGCTGCAGTTGAAAACGCATAATGCGAA
R1847apJBTetCFLAG	TCTTTGTAGTCCATCTGCAGGAACTTAATCGAATCACGTT
F1869pJBTetCFLAG	GTGATAGAGAAAAGCTGCAGATGTCAAAAATCAAGTGGTT
R1869pJBTetCFLAG	TCTTTGTAGTCCATCTGCAGGATAGTATTTTGCGGACTTA
F1902pJBTetCFLAG	GTGATAGAGAAAAGCTGCAGATGAGAAGTAATAGATACAG
R1902pJBTetCFLAG	TCTTTGTAGTCCATCTGCAGGTGTAACCCCGCCGGTAACG
F1910pJBTetCFLAG	GTGATAGAGAAAAGCTGCAGGTGAAGAACCGTTTGACTGC
R1910pJBTetCFLAG	TCTTTGTAGTCCATCTGCAGCTTTTCTACCCGGTCGATTT
F1930apJBTetCFLAG	GTGATAGAGAAAAGCTGCAGATGAATTTTTTTAAAATTAG
R1930apJBTetCFLAG	TCTTTGTAGTCCATCTGCAGAGACTGAGGAACCATACATT
F1984pJBTetCFLAG	GTGATAGAGAAAAGCTGCAGATGAAAAAATTGATTCTATC
F1984-SPpJBTetCFLAG	GTGATAGAGAAAAGCTGCAGATGAATCCTTTTTCACAATTGGT
R1984pJBTetCFLAG	TCTTTGTAGTCCATCTGCAGTGGTTTATTACATGTTGAAT
F2027pJBTetCFLAG	GTGATAGAGAAAAGCTGCAGATGCGATCTTTTAAATTTAT
R2027pJBTetCFLAG	TCTTTGTAGTCCATCTGCAGTTCAACCTCTGAGCGAATGG
F2029pJBTetCFLAG	GTGATAGAGAAAAGCTGCAGATGCGACGTTTACTTGTTAG
R2029pJBTetCFLAG	TCTTTGTAGTCCATCTGCAGATCTTTCTTTGACTTCGATA
F2072pJBTetCFLAG	GTGATAGAGAAAAGCTGCAGATGCGTTATCCGAAATTCAG

R2072pJBTetCFLAG	TCTTTGTAGTCCATCTGCAGAAATCCAATTTCTTGTTGAC
F2079pJBTetCFLAG	GTGATAGAGAAAAGCTGCAGATGAGCTATATTAAACGAGA
R2079pJBTetCFLAG	TCTTTGTAGTCCATCTGCAGGCCGCAGCCATGACCTCCGC
